# Plk1 promotes renal tubulointerstitial fibrosis by targeting autophagy/lysosome axis

**DOI:** 10.1038/s41419-023-06093-4

**Published:** 2023-08-29

**Authors:** Yang Du, Yaqiong Shang, Yun Qian, Yan Guo, Shuang Chen, Xiuli Lin, Weidong Cao, Xiaomei Tang, Anning Zhou, Songming Huang, Aihua Zhang, Zhanjun Jia, Yue Zhang

**Affiliations:** 1 Department of Nephrology, Children’s Hospital of Nanjing Medical University, Guangzhou Road #72, Gulou District, 210008 Nanjing, China; 2 Jiangsu Key Laboratory of Pediatrics, Nanjing Medical University, Hanzhong Road #140, Gulou District, 210029 Nanjing, China; 3 Nanjing Key Laboratory of Pediatrics, Children’s Hospital of Nanjing Medical University, Gulou District, Guangzhou Road #72, 210008 Nanjing, China

**Keywords:** Chronic kidney disease, Kidney

## Abstract

The prevalence of chronic kidney disease (CKD) has been increasing over the past decades. However, no effective therapies are available for delaying or curing CKD. Progressive fibrosis is the major pathological feature of CKD, which leads to end-stage renal disease (ESRD). The present study showed that Polo-like kinase 1 (Plk1) was upregulated in the kidneys of CKD patients and mice subjected to unilateral ureteral obstruction (UUO) with location in proximal tubules and tubulointerstitial fibroblasts. Pharmacological inhibition, genetic silencing or knockout of Plk1 attenuated obstructive nephropathy due to suppressed fibroblast activation mediated by reduced autophagic flux. We found Plk1 plays a critical role in maintaining intralysosomal pH by regulating ATP6V1A phosphorylation, and inhibition of Plk1 impaired lysosomal function leading to blockade of autophagic flux. In addition, Plk1 also prevented partial epithelial-mesenchymal transition (pEMT) of tubular epithelial cells via autophagy pathway. In conclusion, this study demonstrated that Plk1 plays a pathogenic role in renal tubulointerstitial fibrosis by regulating autophagy/lysosome axis. Thus, targeting Plk1 could be a promising strategy for CKD treatment.

## Introduction

Chronic kidney disease (CKD) is a leading cause of mortality and morbidity worldwide, and its prevalence has been increasing over the past decades. Renal replacement therapy (RRT) is yet the primary treatment for end-stage renal disease (ESRD) patients that does not actually improve kidney function and also creates a heavy economic burden in several countries [1, 2]. Although intensive research has been carried out worldwide, the pathogenesis is still elusive, and effective pharmacological therapies are lacking. The common pathological feature of CKD is the loss of tubular cells and progressive interstitial fibrosis. Myofibroblast activation and partial epithelial-mesenchymal transition (pEMT) are the key steps of fibrosis that synthesize and deposit extracellular matrix and progressively destroy kidney structure [3, 4]. Several studies have determined that the extent of tubulointerstitial involvement is correlated better with renal function deterioration than glomerular changes. Thus, targeting fibroblast cell activation and pEMT are critical strategies for attenuating fibrosis.

Some studies showed that blocking cell cycle progression ameliorated fibrosis. Gli2 deletion-induced myofibroblast-specific cell-cycle arrest limited kidney fibrosis [5]. Silencing of cyclinE1 significantly ameliorated liver fibrosis and inflammation [6]. In CKD patients or obstructive mice model, we observed that Plk1, a critical mitotic regulator, was upregulated in kidneys. Plk1 belongs to a family of conserved serine/threonine kinases with a polo-box domain and plays a critical role in the initiation of mitosis, centrosome maturation, bipolar spindle formation, and cytokinesis. Thus, it actively regulates the G2/M transition, mitosis, mitotic exit, and cytokinesis. Plk1 protein and activity are low in G1, accumulate during S and G2/M phases, and are rapidly reduced in the late stages of mitosis [7]. Due to its key role in the eukaryotic cell cycles, Plk1 has been one of the most validated drug targets for cancer treatment. Multiple Plk1 inhibitors have been in clinical trial for anticancer therapy [8–10]. Moreover, multiple non-mitotic functions of Plk1 have been reported, such as metabolism, cancer cell EMT, mTOR regulation, and vascular homeostasis [11–15]. Recently, Chen et al. reported that inhibition of Plk1 limits liver fibroblast activation and liver fibrosis [16]. Zhang et al. found that Plk1 inhibitor BI2536 attenuates podocyte injury and mesangial cell activation in diabetic nephropathy [17]. However, the role of Plk1 in kidney tubular interstitial fibrosis has not been reported. In this study, we explored the role of Plk1 in the classical kidney fibrosis model, UUO, and cultured fibroblast and renal tubular epithelial cells by using specific Plk1 inhibitors, genetic silencing, and heterozygous global knockout mice. The results supported that inhibition of Plk1 ameliorated kidney fibrosis by suppressing fibroblast activation and partial EMT. This study, for the first time, revealed that inhibition of Plk1 causes dysfunction of the lysosome by dephosphorylating V-ATPase, thus impairing autophagy flux.

## Materials and methods

### Human study

Human kidney tissues were obtained from Children’s Hospital of Nanjing Medical University and proper informed consent was obtained from all human subjects. The study was approved by the Research Ethics Board of Children’s Hospital of Nanjing Medical University.

### Animal study

Male mice on C57BL/6 background aged 8–12 weeks (20–25 g) were purchased from Beijing Vital River Laboratory Animal Technology Co., Ltd. Animals were housed in the animal facility of Nanjing Medical University under controlled conditions (23–25 °C) and a 12/12 h light–dark cycle. Food and water were available ad libitum. All animal study protocols were approved by the Institutional Animal Care and Use Committee of Nanjing Medical University. Mice were randomly divided into four groups, and the researchers were blinded to these groups as much as possible, but due to the requirements of the Plk1 global knockout mice system, it was not possible to blind the mouse groups.

On day 0, mice were anesthetized with pentobarbital sodium (50 mg/kg) intraperitoneally and then subjected to UUO surgery, whereby the left ureter was exposed through a mid-abdominal incision and ligated twice using a 4-0 silk suture. For the sham group, the left ureter was exposed without ureteral ligation. On day 2, mice subjected to Plk1 silencing were injected with 2 mL of Plk1-shRNA plasmid (pPLK/GFP+Puro-mPlk1 shRNA, Public Protein/Plasmid Library, China) through a lateral tail vein in 10 sec according to the hydrodynamic-based gene delivery approach [18]. For the drug treatment group, Plk1 inhibitor, BI6727 (Selleckchem, S2235, USA) (15 mg/kg, dissolved in corn oil) was administered by gavage on day 3 and 5 after UUO surgery. Dosing was based on published studies [19]. Plk1 wild-type and knockout mice were subjected to UUO surgery, and contralateral kidneys were used as the control. All mice were euthanized on day 7 after surgery for tissue analysis.

Plk1 global knockout mice were generated using CRISPR/Cas9 technology. According to the structure of *Plk1* gene, exons 2–3 of Plk1 were selected for deletion (Supplementary Fig. S6A). Cas9 and sgRNA were microinjected into the fertilized eggs of mice with C57BL/6 background. Fertilized eggs were transplanted to obtain positive F0 mice that were confirmed by polymerase chain reaction (PCR) and sequencing. A stable F1 generation mouse model was obtained by mating positive F0 generation mice with C57BL/6 mice. Reportedly, homozygous ablation of Plk1 leads to embryonic lethality. Hence, heterozygous mice were used for the experiments. All procedures of generation for Plk1 knockout mice were conducted in Model Animal Research Center of Nanjing University (GemPharmatechCo., Ltd). The two pairs of primers were used for tail genotyping: 5’-ATGGAAAGGCTTCTAGCGAGGG-3’, 5’-CCCTCAGAAGGATGCAAGTGAC-3’with PCR product 303 bp for mutant and 5’-TAACCTAGCACTGAGCCAGACCC-3’, 5’-CCCTGAACCCTTCCACTGTACTG-3’ with PCR product 446 bp for wild-type (WT). Hence, WT mice show one band (446 bp) and knockout (KO) mice show two bands (303 and 446 bp).

### Cell culture

Rat kidney fibroblast cells NRK49F or mouse primary tubular epithelial cells mPTC were purchased from ATCC and were cultured at 37 °C under 5% CO_2_ in DMEM or DMEM/F12 medium containing 10% FBS supplemented with penicillin-streptomycin (100 IU/mL and 100 mg/mL). At 70–80% confluency, the cells were transferred to 2% FBS media for 24 h. Then, the cells were exposed to different treatments as indicated. Fibroblast activation or tubular cell pEMT were induced by human recombinant TGF-β1 (Peprotech, 100-21, NJ, USA) at 10 ng/mL and 15 ng/mL in vitro, respectively. BI6727 (Selleck, S2235, TX, USA), Chloroquine diphosphate (CQ, Apexbio, A8628, USA), Concanamycin A (MedChemExpress, HY-N1724, USA) and Bafilomycin A1 (Baf1-A1, Selleckchem, S1413, USA) was added at different concentration 24 h before TGF-β1 stimulation. Plk1-shRNA (pLKO.1-mPlk1 shRNA, species of mouse, Public Protein/Plasmid Library, China), Plk1 siRNA (species of rat), ATP6V1A-shRNA (pLKO.1-rAtp6v1a shRNA for NRK49F, pLKO.1-mAtp6v1a shRNA for mPTC, Public Protein/Plasmid Library, China), or CyclinB1-shRNA plasmid (pLKO.1-rCcnb1-shRNA, Public Protein/Plasmid Library, China) was transfected using Lipofectamine 2000 reagent (Invitrogen, 11668019, USA) 24 h before TGF-β1 stimulation.

### Cell proliferation and cell cycle analysis

Cell proliferation was analyzed by CCK8 (Beyotime Biotechnology, C0037, China) and Edu-488 kit (Beyotime Biotechnology, C0071S) according to the manufacturer’s instructions. For cell cycle analysis, cells were treated as needed, trypsinized, and collected by centrifugation. After phosphate-buffered saline (PBS) washes, cells were fixed in 4% paraformaldehyde for 10 min at 4 °C and permeabilized with ice-cold 0.1% Triton X-100 for 5 min. Then, the cells were stained with DAPI (Beyotime Biotechnology, C1005, China) for 30 min at room temperature. Cell cycle distribution was analyzed by flow cytometry (FACS, BD Biosciences, USA).

### Quantitative PCR (qPCR) analysis

Total RNA was isolated from kidney tissue or cells using TRIzol reagent (Takara Bio Inc., 9109, Japan). Reverse transcription was performed using HiScript II Q RT SuperMix (Vazyme Biotech Co., R222-01, China). qRT-PCR was carried out on the ABI7500 instrument (Applied Biosystem, USA) with SYBR green master mix (Vazyme Biotech Co., Q131-02). The primer sequences are listed in Table 1. Melting curves were utilized to ensure the specificity of the PCR product. The data were calculated using the 2(-Delta Delta C(T)) method, and the relative expression of the target gene was normalized to that of *GAPDH*.

**Table 1:** Primer sequences for qRT-PCR.

Gene	Assession No.	Primer sequence (5ʹ–3ʹ)
m-*Plk1*-F	NM_011121.4	ACAAGAGGAGGAAGGCACTG
m-*Plk1*-R		ATTCCACTTTGGTTGCCAAG
m-*Acta2*-F	NM_007392.3	CCCAGACATCAGGGAGTAATGG
m-*Acta2*-R		TCTATCGGATACTTCAGCGTCA
m-*Col1a1*-F	NM_007742.4	TAAGGGTCCCCAATGGTGAGA
m-*Col1a1*-R		GGGTCCCTCGACTCCTACAT
m-*Col3a1*-F	NM_009930.2	CAGGACCTAAGGGCGAAGATG
m-*Col3a1*-R		TCCGGGCATACCCCGTATC
m-*Col4a1*-F	NM_009931.2	AACAACGTCTGCAACTTCGC
m-*Col4a1*-R		CTTCACAAACCGCACACCTG
m-*FN1*-F	NM_001276413.1	ATGTGGACCCCTCCTGATAGT
m-*FN1*-R		GCCCAGTGATTTCAGCAAAGG
m-*IL1β*-F	NM_008361.4	GCAACTGTTCCTGAACTCAACT
m-*IL1β*-R		ATCTTTTGGGGTCCGTCAACT
m-*IL6*-F	NM_031168.2	TAGTCCTTCCTACCCCAATTTCC
m-*IL6*-R		TTGGTCCTTAGCCACTCCTTC
m-*Ccl2*-F	NM_011333.3	TTAAAAACCTGGATCGGAACCAA
m-*Ccl2*-R		GCATTAGCTTCAGATTTACGGGT
m-*GAPDH*-F	NM_001289726.2	AGGTCGGTGTGAACGGATTTG
m-*GAPDH*-R		TGTAGACCATGTAGTTGAGGTCA
r-*Actb*-F	NM_031144.3	GTCCACCCGCGAGTACAAC
r-*Actb*-R		GGATGCCTCTCTTGCTCTGG
r-*Plk1*-F	NM_017100.2	AGTACCTGCACCGCAATCAA
r-*Plk1*-R		CAGGGTCTTCTTCCGTTCCC

### Western blotting

Kidney or cell samples were homogenized in RIPA lysis buffer (Beyotime Biotechnology, P0013K, China) containing a protease inhibitor cocktail (Roche, 04693132001, Switzerland). The supernatants were obtained by centrifugation of the homogenates at 12,000 rpm, 4 °C for 15 min), and the protein concentration was measured using the BCA protein assay kit (Thermo Scientific, 23227, USA). Total protein was separated by SDS-PAGE and blotted onto PVDF membranes. Then, the blots were probed at 4 °C overnight in the primary antibody before exposing to horseradish peroxidase-conjugated secondary antibody and chemiluminescent substrate (Tanon, 180-501, China). Densitometry analysis was carried out using Image Lab software (Bio-Rad, USA) using GAPDH (Abclonal, AC033, China) as a reference. The primary antibodies were as follows: anti-Collagen III (Bioss antibodies, bs-0549R, China), anti-α-SMA (Abcam, ab7817, UK), anti-pH3 (Cell Signaling Technology, 53348T, USA), anti-fibronectin (Abcam, ab2413, UK), anti-cyclin B1 (Cell Signaling Technology, 4138T, USA), anti-Plk1 (Abcam, ab17057, UK), anti-LC3 (Novus Biological, NB100-2220, USA), anti-P62 (Cell Signaling Technology, 5114S, USA for cell Western blotting; Abcam, ab91526, UK for mice Western blotting), anti-Smad2 (Cell Signaling Technology, 5339, USA), anti-p-Smad2 (Cell Signaling Technology, 3108T, USA), anti-ATP6V1A (Abcam, ab199326, UK), anti-p-p65-NF-κB (Cell Signaling Technology, 3033S, USA). The original data is available in Supplementary Material.

### Histology and immunostaining

Kidney samples were fixed in 10% formalin and embedded in paraffin. In all, 4-μm-thick sections were used for Masson and hematoxylin-eosin (HE) staining. For immunohistochemical staining, paraffin-embedded kidney sections were deparaffinized and hydrated, followed by antigen retrieval. Endogenous peroxidase activity was quenched by 3% H_2_O_2_. Then, the sections were blocked with 10% normal donkey serum, followed by incubation with anti-Collagen III (Bioss antibodies, bs-0549R, China), anti-fibronectin (FN, Abcam, ab2413, UK), anti-F4/80 (Cell Signaling Technology, 70076T, USA), anti-Plk1 (Abclonal, A2548, China). Images were analyzed and quantified using Image-ProPlus Software. Immunofluorescence staining was performed using cryosections and primary antibody anti-α-SMA (Cell Signaling Technology, 19245S, USA), anti-FSP1 (Proteintech, 66489-1-Ig, USA), and anti-Plk1 (Abcam, ab17057, UK). The staining was detected by Alexa Fluor 488-conjugated (green, Molecular Probes, Invitrogen, A21202, USA) or Alexa Fluor 555-conjugated (red, Molecular Probes, Invitrogen, A31572, USA) anti-IgG as secondary antibody. Nuclei were stained with DAPI (Beyotime Biotechnology, C1005, China). Fluorescein-labeled Lotus tetragonolobus lectin (LTL) (Vector Laboratories, FL-1321, USA) was stained for proximal tubule location. Fluorescence images were captured under a fluorescence microscope (Olympus, USA).

For F-actin staining, 48 h after Plk1-siRNA transfection, NRK49F cells were fixed with 4% paraformaldehyde at room temperature for 20 min, followed by treatment with 0.1% triton X-100 for 10 min at room temperature. Subsequently, phalloidin (Yeasen, 40737es75, China) and anti-α-SMA (Abcam, ab7817, UK) antibodies were applied.

### Autophagic flux assay

RFP-GFP-LC3 plasmid (Invitrogen, P36239, USA) was transfected with Lipofectamine 2000 (Invitrogen, 11668-019, USA) into cells according to the manufacturer’s instructions. After 4 h, cells were pretreated with BI6727 or transfected with Plk1-siRNA in fresh medium for another 4–6 h, followed by TGF-β1 stimulation for 24–48 h.

### Lysosensor staining

LysoSensor DND-189 (Invitrogen, L7535, USA) was used to measure the lysosomal pH. After treatment with BI6727 for 24 h, NRK49F or mPTC cells were incubated in prewarmed (37 °C) probe-containing medium for 40 min. Then, the probe-containing medium was replaced with loading buffer, and the cells were observed under a fluorescence microscope.

### Immunoprecipitation

NRK49F cells were treated with BI6727 for 24 h and homogenized. Cell extracts were immunoprecipitated with rat anti-ATP6V1A antibody (Abcam, ab199326, UK) and protein A/G agarose beads (Bimake, TX, B23201, USA). Then, the precipitates were analyzed by Western blotting with rabbit p-Serine (p-Ser) (Santa Cruz Biotechnology, sc-81514, CA, USA) and p-Threonine (p-Thr) antibodies (Santa Cruz Biotechnology, sc-5267, CA, USA).

### Electron microscopy

NRK49F cells were treated with BI6727 or Plk1 siRNA for 24–48 h. The number of cells was more than 10^6^ per well. Cell culture medium was discarded, and cells were digested with trypsin (appropriate amount of FBS was added to terminate digestion), Then equal volume electron microscope fixative was added to cells for 5 min. Cell suspension was transferred into 1.5 ml sharp bottom EP tube and centrifuged at 800–1200 rpm for 3 min to precipitate cells into clusters. Fresh electron microscope fixative were added and cells were kept at room temperature for more than 30 min. Cell sections were finally analyzed using transmission electron microscope.

### Statistical analysis

Data depicted in the graphs were represented as means±standard error of mean (SEM) for each group. Multiple group comparison was made using one-way analysis of variance (ANOVA). The differences between the two groups were determined by Student’s *t* test. Statistically significant differences were detected between mean values in each graph. *P* < 0.05 showed significant difference. The statistical analyses were conducted by using GraphPad (Prism 7).

## Results

### Plk1 is upregulated in CKD patients and mice

**Fig. 1:**
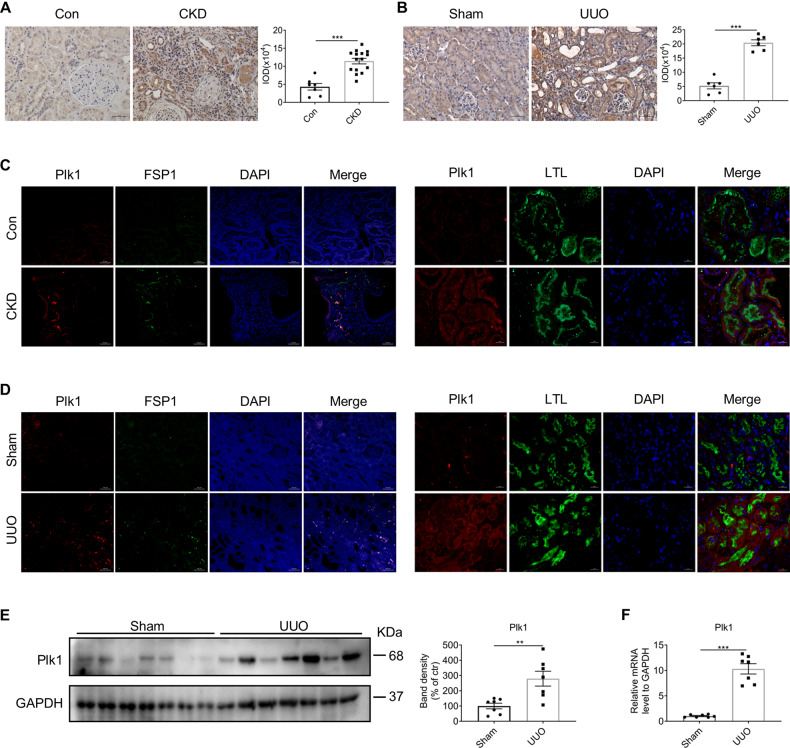
Expression of Plk1 in patients and mice with kidney fibrosis. **A** Representative image of immunohistochemistry staining of Plk1 in kidneys of control and CKD patients. scale bar = 50 µm. *n* = 7–15. **B** Representative image of immunostaining of Plk1 in kidneys of sham and UUO mice. scale bar = 50 µm. *n* = 6. **C** Representative image of double immunofluorescent staining of Plk1/FSP1 (left) or Plk1/LTL (right) in control and CKD patient kidneys. **D** Representative image of double immunofluorescent staining of Plk1/FSP1 (left) or Plk1/LTL (right) in sham and UUO mice kidneys. scale bar = 50 µm. **E**, **F** Western blot and qRT-PCR analysis of Plk1 in sham and UUO mice. *n* = 7. *P < 0.05, **P < 0.01, ***P < 0.001.

Initially, we observed increased expression of Plk1 in kidneys of CKD patients (Fig. 1A). Biopsy kidney samples from CKD patients with up to 65% fibrosis area were selected and stained for Plk1. Peri-tumor kidney sections were used as normal control. Double immunofluorescence staining with the proximal tubule marker LTL or fibroblast marker FSP1 exhibited Plk1 expression in both kidney proximal tubular epithelial cells and fibroblasts in CKD patients (Fig. 1C).

Since UUO is an established animal model causing substantial kidney interstitial fibrosis, we examined Plk1 expression in mice subjected to UUO surgery. Compared to the sham group, the expression of Plk1 in the kidneys of UUO mice was increased significantly at both protein and mRNA levels (Fig. 1B, E, F). Double immunofluorescence staining with LTL and FSP1 also located Plk1 in both proximal tubular epithelial cells and interstitial fibroblast cells (Fig. 1D).

Furthermore, the Nephroseq database (https://nephroseq.com/resource/login.html) was used to explore the correlation between Plk1 and CKD. The results showed that Plk1 expression was significantly increased in renal biopsies of CKD patients (Supplementary Fig. S4A). For animal model, the platform of Gene Atlas of Reversible Unilateral Ureteric Obstruction Model (rUUO) (http://www.ruuo-kidney-gene-atlas.com/) was used and the analysis showed that Plk1 expression in kidney of UUO mice was significantly higher than sham group (Supplementary Fig. S4B). These data further indicated the association of Plk1 with CKD.

### Systemic knockdown of Plk1 attenuates renal interstitial fibrosis in UUO mice

**Fig. 2:**
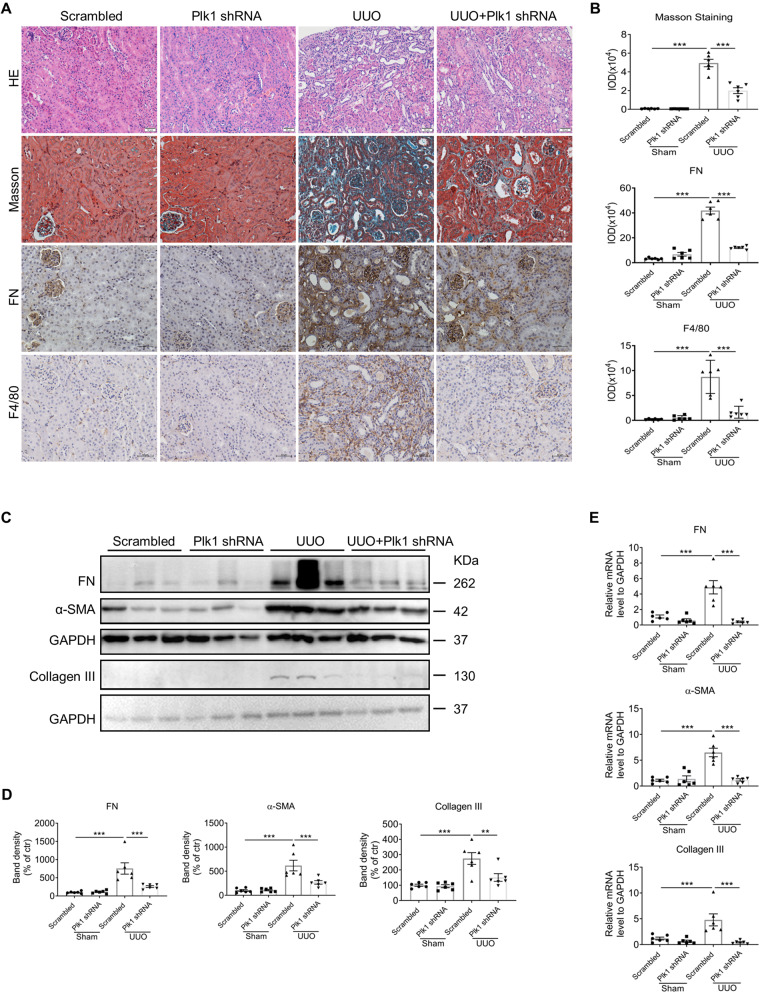
Silencing of Plk1 protects against kidney fibrosis in UUO mice. **A**, **B** Representative images of HE and Masson staining and immunohistochemistry detection of FN, F4/80 in kidneys of sham and UUO mice with or without Plk1 silencing. Scale bar = 50 µm. **C**, **D** Western blot analysis of FN, α-SMA, and Collagen III in kidneys of different groups of mice. **E** qRT-PCR analysis of FN, α-SMA, and Collagen III in different groups of mice. *n* = 6. *P < 0.05, **P < 0.01, ***P < 0.001.

To investigate the role of Plk1 in the pathogenesis of kidney fibrosis, mice were injected intravenously with the expression vector encoding Plk1-shRNA through a hydrodynamic-based gene delivery approach, as reported previously [18, 20]. In sham mice, Plk1 protein was significantly reduced by about 30% by Plk1-shRNA transfection (Supplementary Fig. S1A). GFP fluorescence and qPCR results also confirmed successful transfection of Plk1-shRNA (Supplementary Fig. S3A, B). In UUO mice, HE and Masson staining revealed the presence of tubular dilation, atrophy or loss, interstitial matrix accumulation, and infiltration of inflammatory cells and these were significantly alleviated by Plk1 knockdown (Fig. 2A). Protein and mRNA level of FN, α-SMA and Collagen III induced by UUO were evidently lower in knockdown mice (Fig. 2C–E). Immunohistochemistry showed reduced FN staining (Fig. 2A). Additionally, inflammation is ameliorated by less F4/80-positive macrophage infiltration (Fig. 2A, B) and the transcript level of MCP1, IL6, and IL1β is reduced (Supplementary Fig. S1B). Taken together, this study suggested that Plk1 participates in the pathogenesis of kidney fibrosis in CKD.

### Pharmacological inhibition of Plk1 alleviates renal interstitial fibrosis in UUO mice

**Fig. 3:**
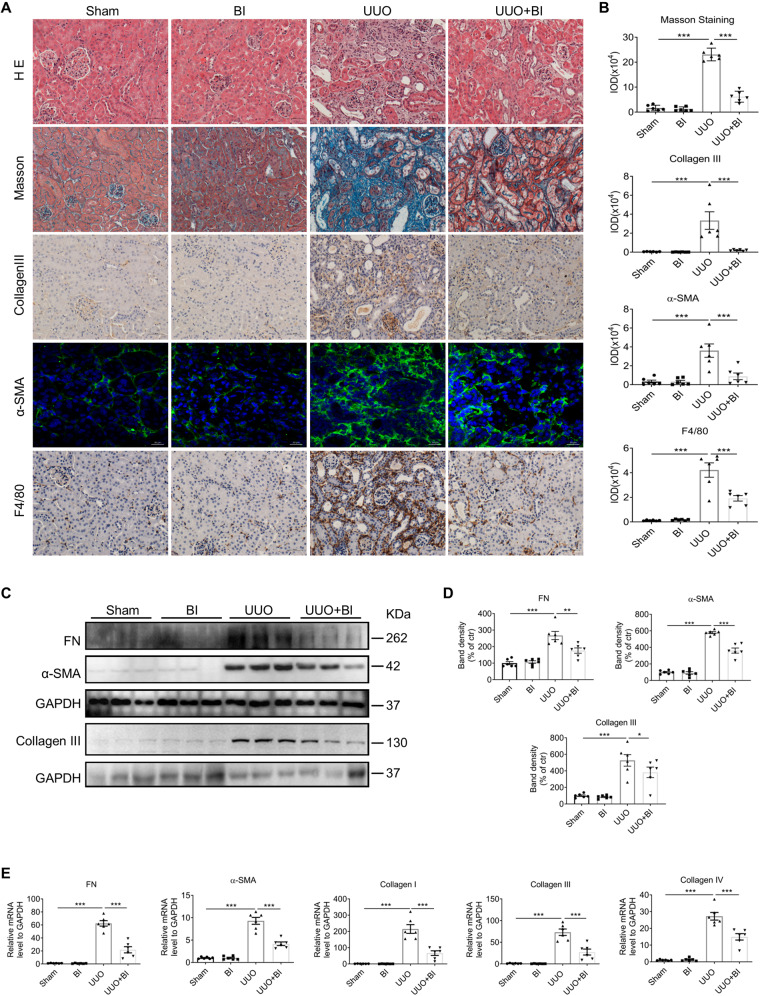
BI6727 attenuates kidney fibrosis in mice subjected to UUO. **A**, **B** Representative images of HE and Masson staining, and immunohistochemistry detection of Collagen III, α-SMA, and F4/80 in sham and UUO mice treated with or without BI6727. Scale bar = 50 µm except scale bar (α-SMA) = 20 µm. **C**, **D** Western blot analysis of FN, α-SMA, and Collagen III in kidneys of all groups of mice. The GAPDH for FN and α-SMA in this blot were from same experiment, same samples and processed in parallel. **E** qRT-PCR analysis of FN, α-SMA, Collagen I, Collagen III, and Collagen IV in kidneys of all groups of mice. *n* = 6. *P < 0.05, **P < 0.01, ***P < 0.001.

To affirm the role of Plk1 in kidney fibrosis, mice subjected to UUO were treated with Plk1 inhibitor, BI6727. HE and Masson staining showed that the obstructive tubular damage was remarkably attenuated and the fibrotic area is largely reduced (Fig. 3A, B) in treated mice. Immunohistochemistry or immunofluorescence staining revealed less staining of Collagen III and α-SMA (Fig. 3A, B). Western blot analysis confirmed decreased expression of FN, Collagen III, and α-SMA in the obstructed kidney of BI6727-treated mice (Fig. 3C, D). Consistent with protein, the mRNA levels of FN, Collagen I, Collagen III, Collagen IV and α-SMA in BI6727-treated UUO mice were reduced compared to untreated UUO mice (Fig. 3E). Furthermore, the number of F4/80-positive macrophages (Fig. 3A, B), p-p65 NF-κB protein and MCP1, IL1β mRNA levels (Supplementary Fig. S1C, D) were decreased in BI6727-treated UUO mice. These results demonstrated that BI6727-mediated pharmacological inhibition of Plk1 achieved a pronounced protective effect against kidney fibrosis in UUO mice model.

### Fibrosis is ameliorated in Plk1+/− mice

**Fig. 4:**
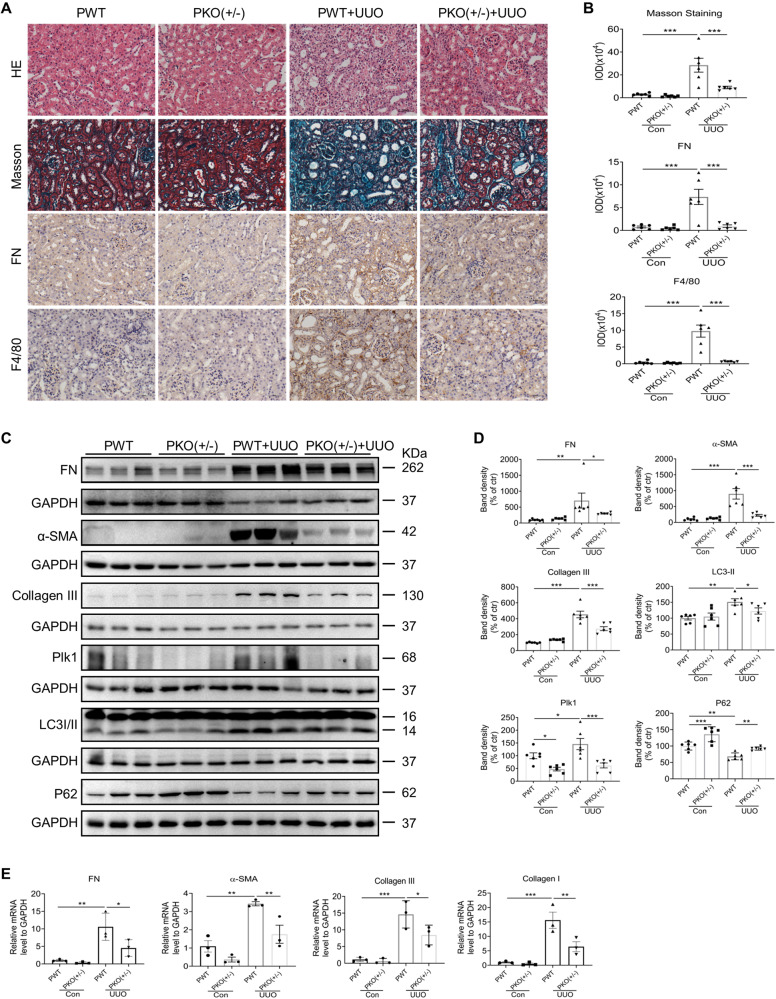
UUO-induced kidney fibrosis is ameliorated in heterozygous Plk1 knockout mice. **A**, **B** Representative images of HE and Masson staining and immunohistochemistry detection of FN, F4/80 in obstructive or contralateral kidneys of PWT and PKO + /− mice and quantification. Scale bar = 50 µm. **C**, **D** Western blot analysis of FN, α-SMA, Collagen III, LC3, Plk1 and P62 in obstructed or contralateral kidneys of PWT and PKO + /− mice, and densitometry analysis of the bands. The GAPDH for Collagen III in this blot was from same experiment, same samples and processed in parallel. **E** qRT-PCR analysis of FN, α-SMA, Collagen I and Collagen III in obstructed or contralateral kidneys of WT and PKO + /− mice. *n* = 3–6. *P < 0.05, **P < 0.01, ***P < 0.001.

Next, we validated our findings in Plk1 transgenic mice. Because homozygous genetic ablation of Plk1 in the germline (Plk1−/−) results in early embryonic lethality, we used heterozygous (Plk1 + /−) mice in this study. The genotyping results are shown in Supplementary Fig. S6B. Plk1 + /− mice are viable and did not show any abnormality. Wild-type (PWT) and Plk1 + /− (PKO + /−) male mice (*n* = 6 each group), 5–12-months-old were selected and subjected to UUO surgery. Contralateral kidneys were used as control. All mice were sacrificed on day 7. Protein analysis confirmed that Plk1 of intact kidney in PKO + /− mice was about 40% less than that in WT mice. Compared to contralateral kidney, the enhanced expression of Plk1 protein in obstructed kidney was suppressed in PKO + /− mice (Fig. 4C, D). Suppressed Plk1 expression significantly ameliorated tubular damage and interstitial fibrosis in obstructed kidney of PKO + /− mice (Fig. 4A). In line with the morphological change, protein and mRNA expression of FN, Collagen I, Collagen III and ɑ-SMA and immunostaining of FN in obstructed kidney of PKO + /− mice was lower than that of PWT mice (Fig. 4A–E). Moreover, in PWT mice, LC3II was induced accompanied with P62 downregulation in obstructed kidneys, which was attenuated in PKO + /− mice (Fig. 4C, D). In addition, we tested the level of p-mTOR in PKO + /− mice and found it was increased compared to PWT mice (Supplementary Fig. S5). These results from transgenic mice confirmed that Plk1 promotes kidney interstitial fibrosis via autophagy regulation.

### Plk1 promotes myofibroblast activation in vitro

**Fig. 5:**
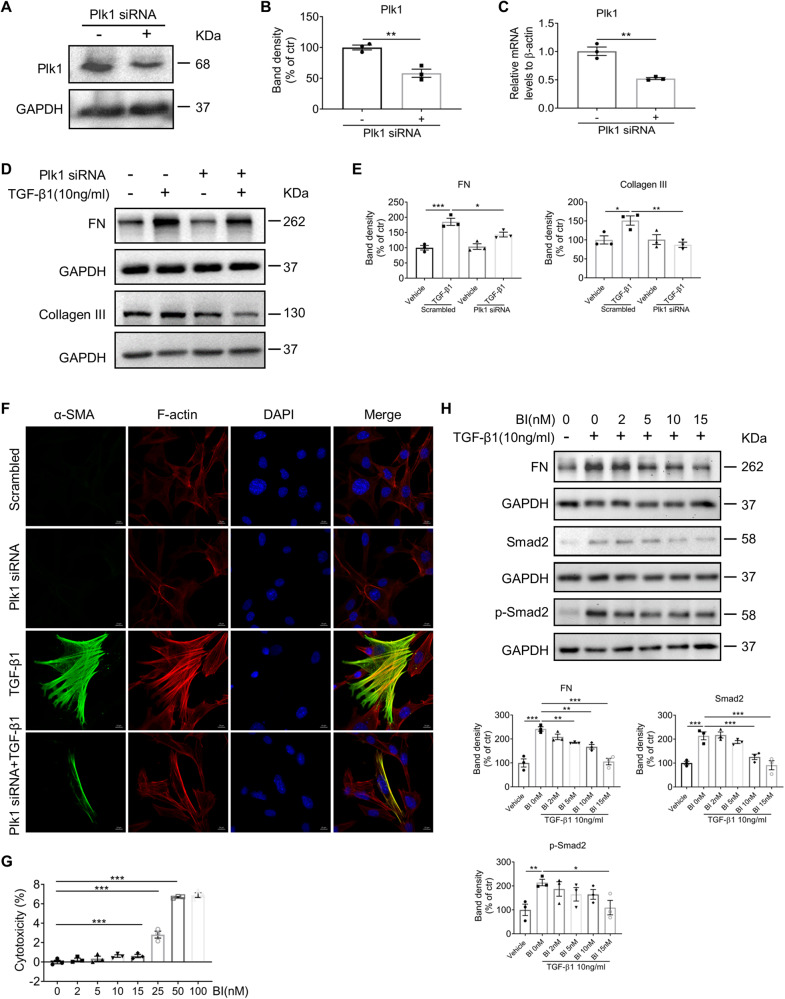
Inhibition of Plk1 prevented TGF-β1-induced fibroblast activation. In NRK49F cells, Plk1 was knocked down by Plk1-siRNA transfection and **A–C** Protein and mRNA level of Plk1 in control and Plk1 knockdown cells. **D**, **E** Western blot analysis of TGF-β1-induced FN and Collagen III expression. **F** Double-staining of α-SMA and phalloidin (F-actin) in response to TGF-β1 in different groups. Scale bar = 10 µm. For pharmacological inhibition of Plk1, NRK49F cells were treated with BI6727. **G** To determine cytotoxicity, LDH release in culture medium was analyzed at different concentrations of BI6727. **H** Western blot analysis of FN, Smad2 and p-Smad2 in cells treated with BI6727 in the presence of TGF-β1. All cell experiments were performed in triplicates. *P < 0.05, **P < 0.01, ***P < 0.001.

Myofibroblast activation is the key step driving the development of fibrosis. The feature of myofibroblast activation includes acquiring of contraction ability by expressing α-SMA and excreting extracellular matrix upon mechanical stretching, autocrine/paracrine cytokines, or other stress. To investigate the role of Plk1 in myofibroblast activation, NRK49F cells were transfected with Plk1-siRNA, followed by TGF-β1 stimulation. As shown in Fig. 5A–C, both Plk1 protein and mRNA levels are reduced by about 50% upon Plk1-siRNA transfection. Protein expression of FN, Collagen III (Fig. 5D, E) and α-SMA positive F-actin stress fiber formation (Fig. 5F) induced by TGF-β1 were significantly reduced by Plk1 knockdown. Next, we tested the effect of BI6727 on fibroblast activation in NRK49F cells. First, we determined the cytotoxicity of BI6727 by LDH release in the culture medium, and results showed that BI6727 concentration <25 nM had no significant cytotoxic effect (Fig. 5G). Thus, cells were treated with BI6727 at 2–15 nM. Western blot analysis revealed that FN accumulation and total and phosphorylated Smad2 induced by TGF-β1 were markedly reduced by BI6727 in a dose-dependent manner (Fig. 5H). These data supported that Plk1 is involved in myofibroblast activation by regulating TGF-β1 signaling pathway.

### Plk1 promotes autophagic flux by affecting lysosomal acidification

**Fig. 6:**
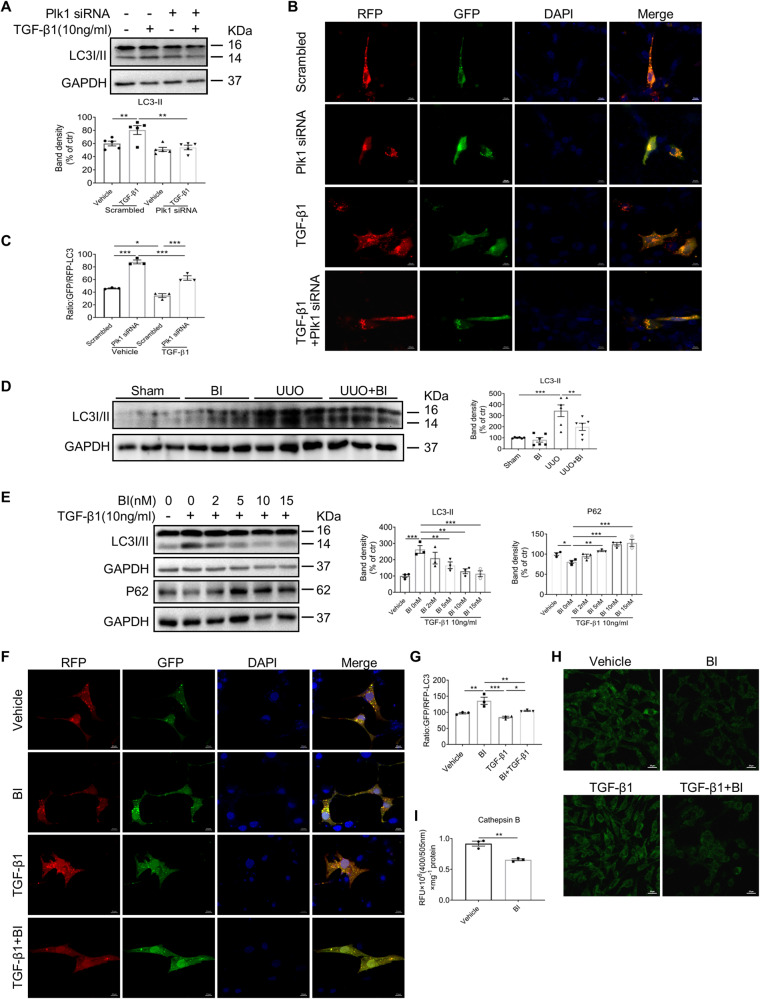
Inhibition of Plk1 suppressed autophagy activity by affecting lysosomal acidification. **A** Western blot analysis of LC3 in NRK49F cells transfected with Plk1 siRNA. *n* = 5. **B**, **C** RFP-GFP-LC3 distribution in NRK49F cells transfected with Plk1 siRNA and stimulated by TGF-β1. The puncta of LC3 was detected with fluorescence microscopy and GFP/RFP ratio was quantified. Scale bar = 10 µm. *n* = 3. **D** Western blot analysis of LC3 in kidneys of UUO mice treated or not with BI6727. *n* = 6. **E** Western blot analysis of LC3 and P62 in NRK49F cells stimulated by TGF-β1 with different concentrations of BI6727. *n* = 3. **F**, **G** RFP-GFP-LC3 distribution in NRK49F cells treated with BI6727 and stimulated by TGF-β1. GFP/RFP ratio was quantified. Scale bar = 10 µm. *n* = 3. **H** Intralysosomal pH detection by lysosensor dye after treatment with TGF-β1 in the presence or absence of BI6727. Scale bar = 20 µm. **I** Cathepsin B enzyme activity in NRK49F in the presence or absence of BI6727. *n* = 3. *P < 0.05, **P < 0.01, ***P < 0.001.

Next, we explored the mechanism underlying the promoting effect of Plk1 on fibroblast activation. Previous studies have shown that Plk1 inhibits or promotes autophagy in tumor cells [15, 21]. Accumulating evidence demonstrated that autophagy plays a key role in the pathogenesis of fibrosis in multiple organs. Hence, we hypothesized that autophagy mediates the effect of Plk1 on fibroblast activation. First, we examined autophagy activity in TGF-β1 stimulation of NRK49F cells. Consistent with the published studies [22, 23], we observed markedly increased expression of LC3II indicating increased autophagy activity. When cells were transfected with Plk1-siRNA, LC3II was significantly reduced (Fig. 6A). To further analyze the dynamic change in autophagic flux, cells were transfected with RFP-GFP-LC3 plasmid to visualize the punctate autophagosomes and autolysosomes. Autophagosomes displayed both green and red (GFP and RFP, respectively) fluorescence. Because the low pH of lysosome quenches the acid-sensitive GFP, autolysosomes appear red (RFP) [24]. TGF-β1 treatment reduced the GFP/RFP ratio slightly but significantly, indicating increased autophagic flux. When Plk1 is knocked down, GFP/RFP ratio was markedly increased either in the presence or absence of TGF-β1, suggesting reduced quench of GFP (Fig. 6B, C). Then, we examined the effect of Plk1 on autophagy in BI6727-treated NRK49F cells. TGF-β1-induced LC3-II upregulation and P62 downregulation were suppressed by BI6727 in a dose-dependent manner (Fig. 6E). GFP/RFP ratio increased in cells treated with BI6727 at 15 nM compared to untreated cells in the presence or absence of TGF-β1 (Fig. 6F, G). Ultrastructural analysis by EM revealed that autolysosome tends to be enlarged with more undigested contents (Supplementary Fig. S2) when Plk1 was knocked down or inhibited by BI6727. In addition, in vivo study showed that LC3II was induced in obstructed kidneys and reversed by BI6727 treatment (Fig. 6D). These results are in agreement with the findings by Stefanie [15] supporting that Plk1 promotes autophagy.

But our data indicated that Plk1 might not only affect the initiation of autophagy, it may also have a role in the downstream autophagic flux. Autophagosomes fuse with lysosomes to form autolysosomes, whose acidic environment activates the enzymes essential for the degradation of biological material. We examined lysosome acidification by LysoSensor dyes, acidotropic probes that accumulate in acidic organelles and exhibit a pH-dependent increase in green fluorescence intensity [25]. In NRK49F cells stimulated by TGF-β1, green fluorescence intensity was significantly increased, suggesting that TGF-β1 enhances lysosomal acidification. When BI6727 was applied, fluorescence intensity was dramatically reduced. BI6727 alone also reduced the fluorescence (Fig. 6H). Meanwhile, Cathepsin B activity was suppressed in BI6727-treated cells suggesting enhanced pH level (Fig. 6I).

**Fig. 7:**
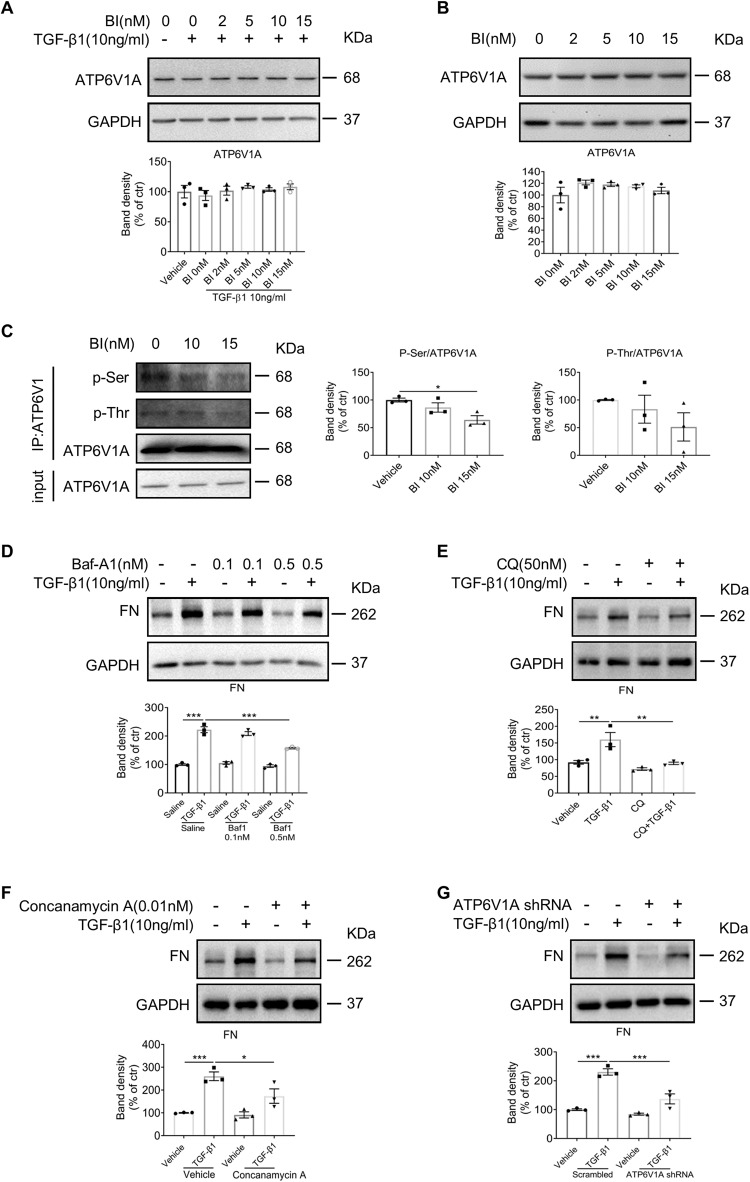
The impact of Plk1 on ATP6V phosphorylation and the role of autophagy on fibroblast activation. **A**, **B** Western blot analysis of ATP6V1A in NRK49F cells treated with BI6727 in the presence or absence of TGF-β1. *n* = 3. **C** Immunoprecipitated ATP6V1A was analyzed by immunoblotting (IB) with antibodies against p-Ser and p-Thr after treatment with different concentrations of BI6727. *n* = 3. **D**, **E** Western blot analysis of FN in NRK49F cells treated with or without Baf-A1/CQ in the presence of TGF-β1. *n* = 3. **F**, **G** Western blot analysis of FN in NRK49F cells treated with Concanamycin A or ATP6V1A-shRNA in the presence of TGF-β1. *n* = 3. *P < 0.05, **P < 0.01, ***P < 0.001.

Since low intralysosomal pH is established and maintained by ATP-dependent proton pump V-ATPase on lysosomal membrane by pumping protons into the lumen, we examined the protein expression of ATP6V1A subunit, but no significant change was observed (Fig. 7A, B). Reportedly, PKA phosphorylates V-ATPase and increases its assembly on membrane in multiple tissues [26]. We examined the phosphorylation state of precipitated ATP6V1A from NRK49F cells. Immunoblotting showed that total phosphorylated serine and threonine residues on ATP6V1A were reduced by BI6727 treatment (Fig. 7C) without affecting the amount of protein. This result indicated Plk1 might regulate V-ATPase function by phosphorylating its subunit ATP6V1A.

Because contradictory results are recorded about the role of autophagy in fibrosis, we further validated the effect of autophagy inhibitors, Baf-A1 and CQ, on fibroblast activation. As shown in Fig. 7D, E, FN expression in response to TGF-β1 in fibroblasts were suppressed by both Baf-A1 (0.1 nM and 0.5 nM) and CQ (50 nM). These results indicated that autophagy is positively correlated with fibroblast activation in current setting.

To understand whether V-ATPase plays a role in fibroblast activation, we applied ATP6V1A-shRNA and another V-ATPase inhibitor (Concanamycin A, 0.01 nM) in NRK49F cells. The results showed that after knock-down of ATP6V1A or inhibition of V-ATPase, the FN level induced by TGF-β1 was significantly reduced (Fig. 7F, G). These data supported that V-ATPase could contribute to the effect of Plk1 on fibroblast activation.

### Plk1 regulates fibroblast cell proliferation and G2/M cell cycle progression

**Fig. 8:**
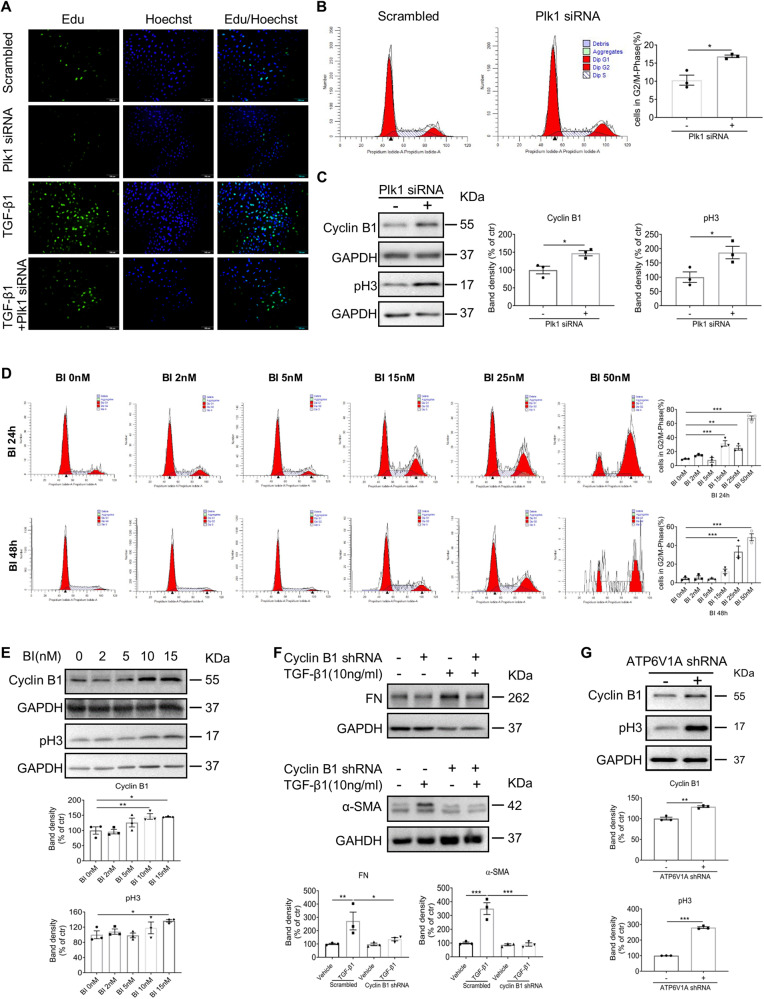
Inhibition of Plk1 prevents cell proliferation and induces cell cycle arrest at G2/M stage in kidney fibroblast cells. **A** EdU staining of cells after Plk1 siRNA transfection. Scale bar=100 µm. **B** Cell cycle analysis by flow cytometry with PI staining. **C** Western blot analysis of CyclinB1 and pH3. Then, NRK49F cells were treated with different concentrations of BI6727. **D** Cell cycle analysis by flow cytometry with PI staining 24 or 48 h after BI6727 treatment. **E** Western blot analysis of CyclinB1 and pH3. **F** Western blot analysis of FN and ɑ-SMA of NRK49F cells stimulated with TGF-β1 when CyclinB1 was knocked down. The GAPDH for FN and ɑ-SMA were from same experiment, same samples and processed in parallel. **G** Western blot analysis of CyclinB1 and pH3 when ATP6V1A was knocked down. All cell experiments were performed in triplicates. *P < 0.05, **P < 0.01, ***P < 0.001.

Moreover, we examined cell proliferation and cell cycle distribution upon Plk1 inhibition. As expected, Plk1 knockdown by siRNA significantly inhibited NRK49F proliferation (Supplementary Fig. S7A) accompanied by morphological changes. Cells with Plk1 knockdown displayed a round morphology, indicating mitotic arrest (Supplementary Fig. S7B). EdU assay and flow cytometry analysis demonstrated reduced DNA synthesis and G2/M arrest in Plk1 knockdown cells (Fig. 8A, B). Both CyclinB1 and pH3 protein accumulates (Fig. 8C). CyclinB1 could not be degraded without Plk1 [27]. BI6727 showed similar effect in dose-dependent manner (Supplementary Fig. S7C, D and Fig. 8D, E). Then, we asked whether other G2/M regulator has a similar effect on fibroblast activation. Therefore, we knocked down CyclinB1 in NRK49F cells and found that TGF-β1-induced FN and ɑ-SMA expression were decreased (Fig. 8F). These data suggested that the antifibrotic effect of Plk1 inhibition is related to its effect on cell cycle arrest.

On the other hand, we knocked down ATP6V1A in NRK49F cells to examine its effect on cell cycle. The result demonstrated that reduced ATP6V1A expression caused Cyclin B1 and pH3 accumulation indicating cell cycle arrest (Fig. 8G). Thus, it seems that autophagy and cell cycle coordinate during fibroblast activation.

### Plk1 promotes partial EMT in tubular epithelial cells

**Fig. 9:**
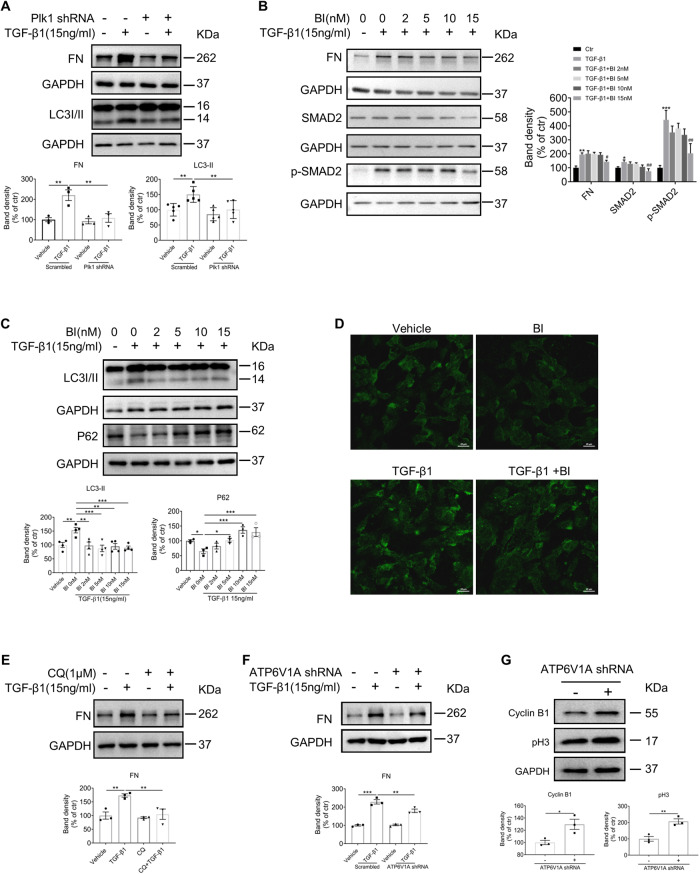
Inhibition of Plk1 prevents pEMT of tubular epithelial cells. **A** mPTC cells were transfected with Plk1 shRNA and stimulated with TGF-β1. FN and LC3 were analyzed by western blot. *n* = 3-5. **B**, **C** mPTC cells were treated with different concentrations of BI6727 and stimulated with TGF-β1. FN, Smad2, p-Smad2, LC3 and P62 were analyzed by western blot. *n* = 3-4. *compared to ctr group, ^#^ compared to TGF-β1 group. **D** Intralysosomal pH detection by lysosensor dye in mPTC cells treated with BI6727 in the presence or absence of TGF-β1. Scale bar=20 µm. *n* = 3. **E** Western blot analysis of FN in mPTC cells treated with CQ in response to TGF-β1. *n* = 3. **F** Western blot analysis of FN in mPTC cells when ATP6V1A is knocked down in response to TGF-β1. *n* = 3. **G** Western blot analysis of CyclinB1 and pH3 when ATP6V1A was knocked down. *n* = 3. *P < 0.05, **P < 0.01, ***P < 0.001. ^#^*P* < 0.05,^##^*P* < 0.01, ^###^*P* < 0.001.

Recent studies have shown that tubular epithelial cells may not experience a complete transition to myofibroblast cells. Instead, they acquired phenotype change, termed partial EMT and produce some profibrotic cytokines/growth factors to induce renal fibroblast activation, which plays a critical role in kidney fibrosis [28]. Therefore, we transfected mPTC cells with Plk1-siRNA. Typically, reduced Plk1 expression suppressed TGF-β1-induced FN accumulation and LC3II upregulation (Fig. 9A). When BI6727 was applied, TGF-β1-induced FN expression was inhibited in a dose-dependent manner, accompanied by reduced total Smad2 and Smad2 phosphorylation (Fig. 9B). The induction of LC3II by TGF-β1 was also prevented by BI6727 as well as P62 accumulation (Fig. 9C). Intralysosomal pH measurement by lysosensor showed that TGF-β1 induced green fluorescence was reduced in BI6727-treated cells (Fig. 9D).

Then, to examine the effect of autophagy on pEMT, we used autophagy inhibitors, CQ, and found FN expression in response to TGF-β1 in mPTC cells was suppressed by CQ (Fig. 9E). Moreover, knocking down ATP6V1A with shRNA also reduced FN level stimulated by TGF-β1 (Fig. 9F). Knocking down of ATP6V1A resulted in Cyclin B1 and pH3 accumulation (Fig. 9G). These data demonstrated that Plk1 is involved in partial EMT of renal tubular cells by regulating autophagy/lysosome axis.

## Discussion

Tubular kidney injury repair and remodeling involves epithelial cells, fibroblasts, and inflammatory cells. Irrespective of cell composition or phase, cell cycle progression is a continual event. However, the role of cell cycle regulator in CKD development is not thoroughly understood. Plk1 is a critical G2/M regulator and is upregulated in both CKD patients and mice. In the present study, we provided substantial evidence supporting that Plk1 plays a critical role in kidney tubular interstitial fibrosis via autophagy/lysosome axis.

UUO mimics various stages of CKD in an accelerated way. In the obstructed kidney, epithelial tubular cells underwent injury, inflammation and death due to hemodynamic change caused by obstructed urine flow, which effectuated maladaptive repair of tubular cells, progressive myofibroblasts activation and delayed resolution of inflammation, termed tubulointerstitial fibrosis [29]. Therefore, we first knocked down Plk1 in UUO mice by transfecting Plk1-shRNA through hydrodynamic tail vein (HTV) injection. Reduced Plk1 expression ameliorated kidney fibrosis, preserved tubular structure, and attenuated inflammation. Then, we selected BI6727, a highly potent and ATP-competitive Plk1 inhibitor, which has entered phase III clinical trial, to treat UUO mice. As expected, BI6727 also has a protective effect. Thus, the results using RNA silencing and small molecule drug supported that Plk1 promotes CKD fibrosis progression.

Immunostaining showed that Plk1 in CKD patients or UUO mice located in both fibroblast and tubular epithelial cells. Since fibroblast activation is the key for fibrosis progression, we examined the impact of Plk1 on fibroblast activation. Activated fibroblasts gain contractile properties that allow their migration and invasion of the wound site by upregulating the stress fiber α-SMA and synthesis of extracellular matrix to maintain tissue integrity. Interestingly, BI6727 and Plk1 knockdown reduced myofibroblast activation, following which TGF-β1/Smad2 pathway is suppressed. The IC50 of BI6727 in vitro is 0.87 nM. Our cellular toxicity assay showed that BI6727 > 25 nM caused serious LDH leakage and induced cell death. So, we treated NRK49F cells with BI6727 at 2–15 nM and observed antifibrotic effect in a dose-dependent manner.

Furthermore, we used heterozygous Plk1 knockout mice to validate the role of Plk1 in kidney fibrosis. No abnormality was observed in PKO + /− mice. UUO-induced kidney fibrosis and enhanced autophagic activity were significantly attenuated in PKO + /− mice. These results are consistent with what we found using BI6727 or Plk1 silencing.

Recently, Plk1 has been reported to regulate autophagy in various cancer cells. To elucidate the mechanism of Plk1-regulated TGF-β1-induced myofibroblast activation, we examined the autophagic activity in vivo and in vitro. In line with previous studies, autophagic activity was enhanced in obstructed kidney and TGF-β1-treated fibroblast cells, but they were prevented by BI6727 or Plk1 knockdown indicating that Plk1 promotes autophagy. In the UUO mice kidney, Plk1 is upregulated suggesting that profibrotic factor induces Plk1 expression. Additionally, it is well known that TGF-β1 promotes fibroblast proliferation and fibrotic response. As a critical cell cycle regulator, Plk1 is probably the downstream of TGF-β1 which has also been demonstrated in cancer cells [30]. In our study, LC3II was upregulated by both TGF-β1 stimulation and high Plk1 expression. Therefore, it is possible that TGF-β1 and Plk1 were upstream regulatory factors of LC3 and autophagy.

Autophagy has been associated with myofibroblast activation by regulating matrix protein synthesis and degradation, intracellular component recycling, extracellular vesicles secretion, and metabolism. However, the findings of various studies are controversial, which could be attributed to various stress conditions, different phases of the stress response, and how autophagy is manipulated [31–38]. In current study, we found blocking autophagy attenuated TGF-β1-induced fibroblast activation, agreeing with Dong and his colleagues who showed that persistent activation of autophagy promotes kidney fibrosis [34]. The inhibitory effect of autophagy on ECM expression indicated the important role of autophagy in this scenario via material recycling, which could affect the fuel supply for ECM synthesis and fibroblast proliferation.

There are studies showing that Plk1 affects the initiation of autophagy through mTOR complex1 or ULK1 [15, 39]. The increased GFP/RFP-LC3 ratio induced by BI6727 or Plk1 silencing indicated autolysosome function might be impaired. We found cells treated with BI6727 have less acidic intralysosomal environment and reduced Cathepsin B activity indicating impaired lysosomal acidification. The pH gradient of the lysosomes is maintained by the proton-pumping V-type ATPase [40, 41], which consumes ATP to pump protons into the lysosome lumen. The V-ATPases are composed of a peripheral V1 domain that carries out ATP hydrolysis and an integral V0 domain responsible for proton transport. V1 has eight different subunits (A-H) present in a stoichiometry of A_3_B_3_CDE_3_FG_3_H, and ATP hydrolysis occurs at three catalytic sites located primarily in subunit A [41]. Moreover, V-ATPase A subunit could be regulated by phosphorylation, leading to enhanced V-ATPase activity [42]; thus, we examined the total level of phosphorylation on serine and threonine residues in precipitated V-ATPase A subunit (ATP6V1A) protein. Although the total protein level of ATP6V1A did not change upon BI6727 treatment, the phosphorylation level on serine and threonine residues of ATP6V1A were reduced by BI6727. While blocking ATP6V1A by shRNA or inhibitor both could ameliorate FN accumulation in response to TGF-β1. Similar results were observed in mPTC cells. Our study provides evidence that Plk1 regulates autophagy not only by autophagosome formation, but also by lysosomal acidification. Published studies have shown that lysosome depletion-triggered autophagy impairment is associated with AKI, podocyte injury, and some other kidney injury [43]. Lysosomal cathepsins are implicated in liver, lung, and kidney fibrosis [44]. In addition, the primary mechanism of Baf-A1 disrupting autophagic flux is also the inhibition of V-ATPase-dependent acidification [45]. Taken together, lysosomal dysfunction, at least partially, mediated the preventive effect of Plk1 inhibition on fibroblast activation. In line with other study, we found p-mTOR was increased in PKO + /− mice indicating negative effect of Plk1 on mTOR pathway. Although mTOR is reported to regulate lysosome function [46], whether mTOR mediated the effect of Plk1 on ATP6V1A phosphorylation needs further investigation.

Fibroblast activation manifested as both phenotype conversion (extracellular matrix synthesis and mobility) and cell cycle entry. We confirmed that inhibition of Plk1 slows down kidney fibroblast cell proliferation and causes G2/M arrest. Taken together, our results indicated that Plk1 is a connector of cell cycle progression and fibrotic phenotype conversion. Thus, we speculated other G2/M regulators might have similar effect. We silenced CyclinB1 in NRK49F cells and observed reduced expression of ɑ-SMA and FN in response to TGF-β1. Another study by Zhang et al. reported that paclitaxel, a mitotic inhibitor, protects against kidney fibrosis [47]. It could be explained that phenotype change and proliferation are two sides of one thing, so interfering with cell cycle progression will also influence fibrotic conversion. Notably, there might be specific mechanism for each cell cycle regulator. That needs to be thoroughly investigated. Taken together, these studies suggested that blocking of G2/M progression might be a potential target for kidney fibrotic disease.

Immunostaining revealed that Plk1 was also located in tubular epithelial cells. In early phase of injury, it could be an indicator of proliferation for repair. However, under persistent hypoxia or other stress conditions in UUO model, epithelial cells arrest at G2/M [48, 49] and produce a variety of profibrotic factors and cytokines [28]. These cells express both epithelial and mesenchymal markers but remain inside tubules, which is termed pEMT, a critical contributor of fibrosis progression. Current study revealed that although tubular cells may not be able to proliferate actively in obstructive conditions, Plk1 still plays a role in promoting pEMT. Reduced acidification of lysosome and attenuated autophagic flux might mediate such an effect. In cancer cells, autophagy activation provides energy and basic nutrients for EMT during metastatic spread [50]. Under UUO condition, autophagy might support pEMT the same way by providing nutrients for phenotype transition.

There are some limitations of our work. We used global knockout mice in this study, while cell-specific conditional knockout mice would be favorable in further understanding the cell-specific role of Plk1 in CKD. Additionally, to better reveal the correlation of Plk1 and CKD in clinical patients, more clinical samples should be collected and analyzed. And it will be our goal in the future studies.

In summary, we discovered a fundamental role of Plk1 in kidney interstitial fibrosis. Upregulation of Plk1 in CKD participates in disease progression by promoting fibroblast activation and pEMT of tubular cells. Plk1 might represent a potential target for kidney interstitial fibrosis.

## Supplementary information

Supplementary Figure

Supplementary Figure legend

aj-checklist

western blot original data

## Data Availability

All data in this study are available upon reasonable request from the corresponding authors.

## References

[1] Ng JK, Li PK (2018). Chronic kidney disease epidemic: how do we deal with it?. Nephrology.

[2] Panizo S, Martinez-Arias L, Alonso-Montes C, Cannata P, Martin-Carro B, Fernandez-Martin JL (2021). Fibrosis in chronic kidney disease: pathogenesis and consequences. Int J Mol Sci.

[3] Humphreys BD (2018). Mechanisms of renal fibrosis. Annu Rev Physiol.

[4] Strutz F, Zeisberg M (2006). Renal fibroblasts and myofibroblasts in chronic kidney disease. J Am Soc Nephrol.

[5] Kramann R, Fleig SV, Schneider RK, Fabian SL, Dirocco DP, Maarouf O (2015). Pharmacological GLI2 inhibition prevents myofibroblast cell-cycle progression and reduces kidney fibrosis. J Clin Invest.

[6] Bangen JM, Hammerich L, Sonntag R, Baues M, Haas U, Lambertz D (2017). Targeting CCL_4_-induced liver fibrosis by RNA interference-mediated inhibition of cyclin E1 in mice. Hepatology..

[7] Kumar S, Sharma AR, Sharma G, Chakraborty C, Kim J (2016). PLK-1: angel or devil for cell cycle progression. Biochim Biophys Acta.

[8] Elsayed I, Wang X (2019). PLK1 inhibition in cancer therapy: potentials and challenges. Future Med Chem.

[9] de Carcer G (2019). The mitotic cancer target polo-like kinase 1: oncogene or tumor suppressor?. Genes.

[10] Park JE, Hymel D, Burke TJ, Lee KS (2017). Current progress and future perspectives in the development of anti-polo-like kinase 1 therapeutic agents. F1000Res.

[11] Raab CA, Raab M, Becker S, Strebhardt K (2021). Non-mitotic functions of polo-like kinases in cancer cells. Biochim Biophys Acta Rev Cancer.

[12] de Carcer G, Wachowicz P, Martinez-Martinez S, Oller J, Mendez-Barbero N, Escobar B (2017). Plk1 regulates contraction of postmitotic smooth muscle cells and is required for vascular homeostasis. Nat Med.

[13] Ma X, Wang L, Huang, Li Y, Yang D, Li T (2017). Polo-like kinase 1 coordinates biosynthesis during cell cycle progression by directly activating pentose phosphate pathway. Nat Commun.

[14] Fu Z, Wen D (2017). The emerging role of polo-like kinase 1 in epithelial-mesenchymal transition and tumor metastasis. Cancers.

[15] Ruf S, Heberle AM, Langelaar-Makkinje M, Gelino S, Wilkinson D, Gerbeth C (2017). PLK1 (polo like kinase 1) inhibits MTOR complex 1 and promotes autophagy. Autophagy..

[16] Chen Y, Chen X, Ji YR, Zhu S, Bu FT, Du XS (2020). Plk1 regulates hepatic stellate cell activation and liver fibrosis through Wnt/beta-catenin signalling pathway. J Cell Mol Med.

[17] Zhang L, Wang Z, Liu R, Li Z, Lin J, Wojciechowicz ML (2021). Connectivity mapping identifies BI-2536 as a potential drug to treat diabetic kidney disease. Diabetes..

[18] Liu F, Song Y, Liu D (1999). Hydrodynamics-based transfection in animals by systemic administration of plasmid DNA. Gene Ther.

[19] Rudolph D, Steegmaier M, Hoffmann M, Grauert M, Baum A, Quant J (2009). BI 6727, a polo-like kinase inhibitor with improved pharmacokinetic profile and broad antitumor activity. Clin Cancer Res.

[20] Suda T, Liu D (2007). Hydrodynamic gene delivery: its principles and applications. Mol Ther.

[21] Tao YF, Li ZH, Du WW, Xu LX, Ren JL, Li XL (2017). Inhibiting PLK1 induces autophagy of acute myeloid leukemia cells via mammalian target of rapamycin pathway dephosphorylation. Oncol Rep.

[22] Zhang J, Jiang N, Ping J, Xu L (2021). TGF-beta1-induced autophagy activates hepatic stellate cells via the ERK and JNK signaling pathways. Int J Mol Med.

[23] Zhang C, Zhang X, Xu R, Huang B, Chen AJ, Li C (2017). TGF-beta2 initiates autophagy via Smad and non-Smad pathway to promote glioma cells’ invasion. J Exp Clin Cancer Res.

[24] Kimura S, Noda T, Yoshimori T (2007). Dissection of the autophagosome maturation process by a novel reporter protein, tandem fluorescent-tagged LC3. Autophagy.

[25] Breton S, Brown D (2013). Regulation of luminal acidification by the V-ATPase. Physiology.

[26] Mcguire C, Stransky L, Cotter K, Forgac M (2017). Regulation of V-ATPase activity. Front Biosci.

[27] Liu X, Erikson RL (2002). Activation of Cdc2/cyclin B and inhibition of centrosome amplification in cells depleted of Plk1 by siRNA. Proc Natl Acad Sci USA.

[28] Sheng L, Zhuang S (2020). New insights into the role and mechanism of partial epithelial-mesenchymal transition in kidney fibrosis. Front Physiol.

[29] Martinez-Klimova E, Aparicio-Trejo OE, Tapia E, Pedraza-Chaverri J (2019). Unilateral ureteral obstruction as a model to investigate fibrosis-attenuating treatments. Biomolecules..

[30] Shin SB, Jang HR, Xu R, Won JY, Yim H (2020). Active PLK1-driven metastasis is amplified by TGF-beta signaling that forms a positive feedback loop in non-small cell lung cancer. Oncogene..

[31] Del PD, Lista P, Malorni W, Giammarioli AM (2013). Fibroblast autophagy in fibrotic disorders. J Pathol.

[32] Gupta SS, Zeglinski MR, Rattan SG, Landry NM, Ghavami S, Wigle JT (2016). Inhibition of autophagy inhibits the conversion of cardiac fibroblasts to cardiac myofibroblasts. Oncotarget..

[33] Ghavami S, Cunnington RH, Gupta S, Yeganeh B, Filomeno KL, Freed DH (2015). Autophagy is a regulator of TGF-beta1-induced fibrogenesis in primary human atrial myofibroblasts. Cell Death Dis.

[34] Livingston MJ, Ding HF, Huang S, Hill JA, Yin XM, Dong Z (2016). Persistent activation of autophagy in kidney tubular cells promotes renal interstitial fibrosis during unilateral ureteral obstruction. Autophagy..

[35] Kim SI, Na HJ, Ding Y, Wang Z, Lee SJ, Choi ME (2012). Autophagy promotes intracellular degradation of type I collagen induced by transforming growth factor (TGF)-beta1. J Biol Chem.

[36] Migneault F, Hebert MJ (2021). Autophagy, tissue repair, and fibrosis: a delicate balance. Matrix Biol.

[37] Shi Y, Hu Y, Wang Y, Ma X, Tang L, Tao M (2021). Blockade of autophagy prevents the development and progression of peritoneal fibrosis. Front Pharmacol.

[38] Tang C, Livingston MJ, Liu Z, Dong Z (2020). Autophagy in kidney homeostasis and disease. Nat Rev Nephrol.

[39] Valianou M, Cox AM, Pichette B, Hartley S, Paladhi UR, Astrinidis A (2015). Pharmacological inhibition of polo-like kinase 1 (PLK1) by BI-2536 decreases the viability and survival of hamartin and tuberin deficient cells via induction of apoptosis and attenuation of autophagy. Cell Cycle.

[40] Mindell JA (2012). Lysosomal acidification mechanisms. Annu Rev Physiol.

[41] Stransky L, Cotter K, Forgac M (2016). The function of V-ATPases in cancer. Physiol Rev.

[42] Alzamora R, Thali RF, Gong F, Smolak C, Li H, Baty CJ (2010). PKA regulates vacuolar H+-ATPase localization and activity via direct phosphorylation of the a subunit in kidney cells. J Biol Chem.

[43] Chen XC, Li ZH, Yang C, Tang JX, Lan HY, Liu HF (2021). Lysosome depletion-triggered autophagy impairment in progressive kidney injury. Kidney Dis.

[44] Fox C, Cocchiaro P, Oakley F, Howarth R, Callaghan K, Leslie J (2016). Inhibition of lysosomal protease cathepsin D reduces renal fibrosis in murine chronic kidney disease. Sci Rep.

[45] Mauvezin C, Neufeld TP (2015). Bafilomycin A1 disrupts autophagic flux by inhibiting both V-ATPase-dependent acidification and Ca-P60A/SERCA-dependent autophagosome-lysosome fusion. Autophagy..

[46] Puertollano R (2014). mTOR and lysosome regulation. F1000Prime Rep.

[47] Zhang L, Xu X, Yang R, Chen J, Wang S, Yang J (2015). Paclitaxel attenuates renal interstitial fibroblast activation and interstitial fibrosis by inhibiting STAT3 signaling. Drug Des Devel Ther.

[48] Lovisa S, Lebleu VS, Tampe B, Sugimoto H, Vadnagara K, Carstens JL (2015). Epithelial-to-mesenchymal transition induces cell cycle arrest and parenchymal damage in renal fibrosis. Nat Med.

[49] Yang L, Besschetnova TY, Brooks CR, Shah JV, Bonventre JV (2010). Epithelial cell cycle arrest in G2/M mediates kidney fibrosis after injury. Nat Med.

[50] Chen HT, Liu H, Mao MJ, Tan Y, Mo XQ, Meng XJ (2019). Crosstalk between autophagy and epithelial-mesenchymal transition and its application in cancer therapy. Mol Cancer.

