# Supplementary material for: Plk1 promotes renal tubulointerstitial fibrosis by targeting autophagy/lysosome axis
Source: Cell Death Dis. 2023 Aug 29;14(8):571. doi: 10.1038/s41419-023-06093-4 (PMC10462727; doi:10.1038/s41419-023-06093-4)

**Supplementary Figure 1**

**Fig. S1**


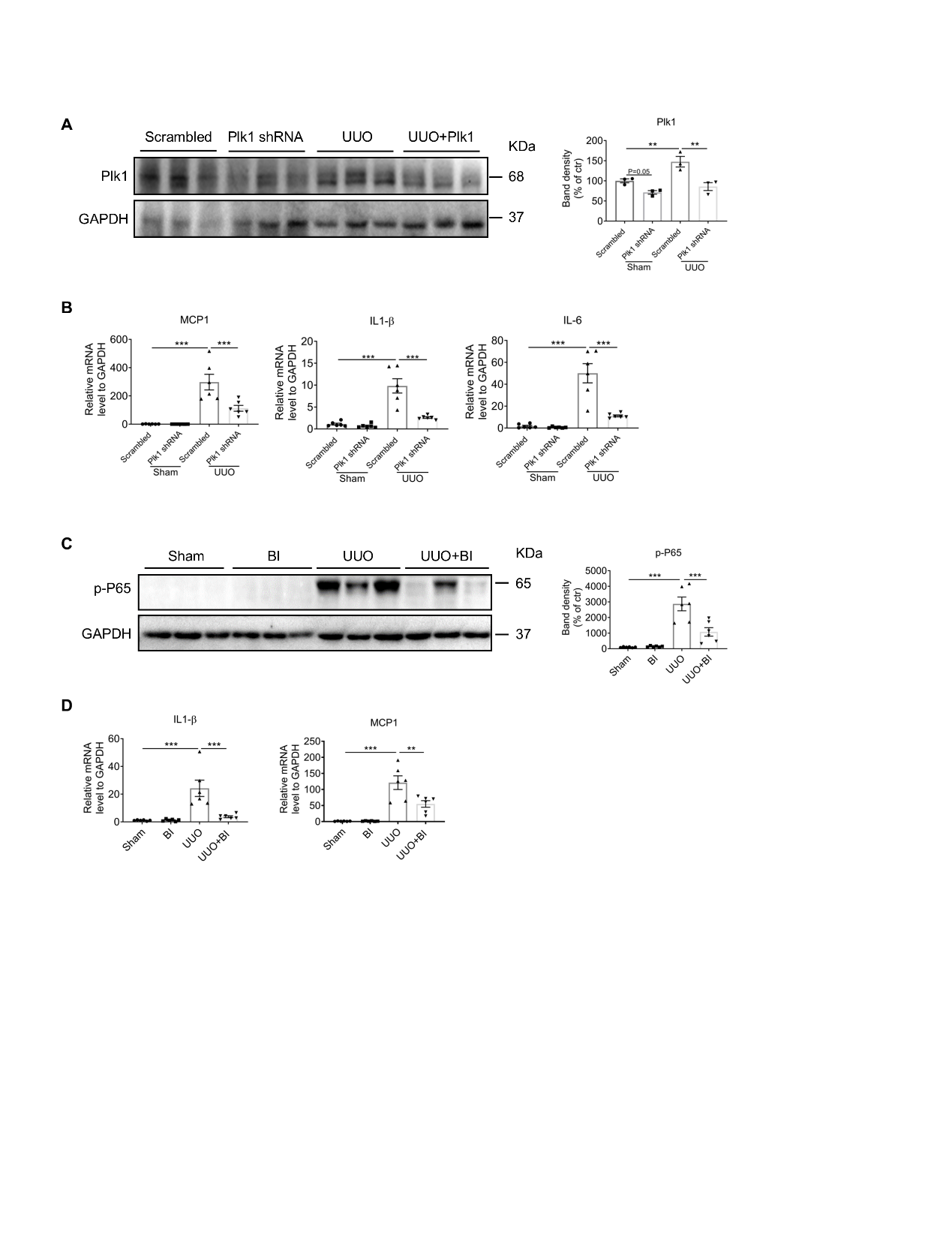


**Supplementary Figure 2**

**Fig. S2**


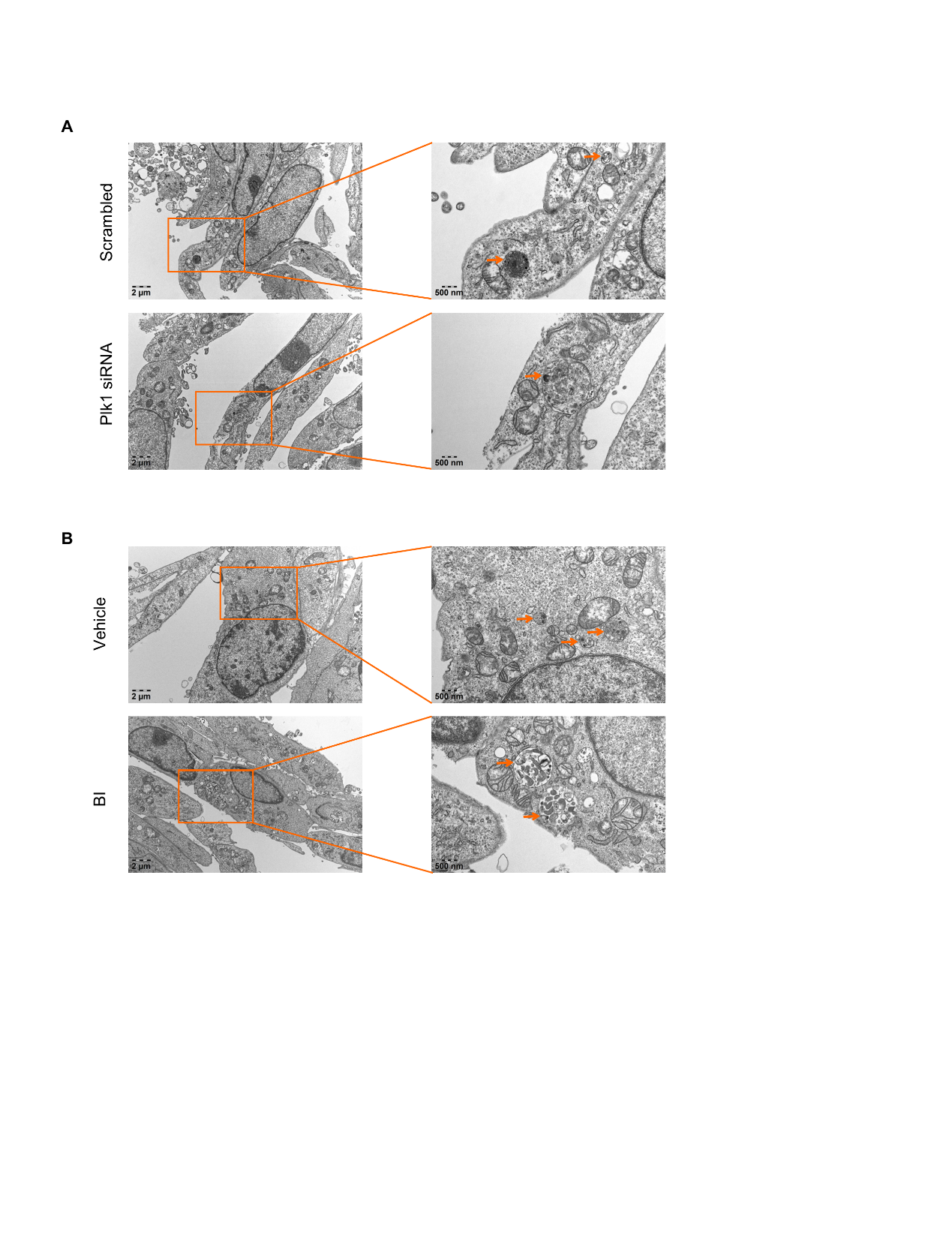


**Supplementary Figure 3**

**Fig. S3**


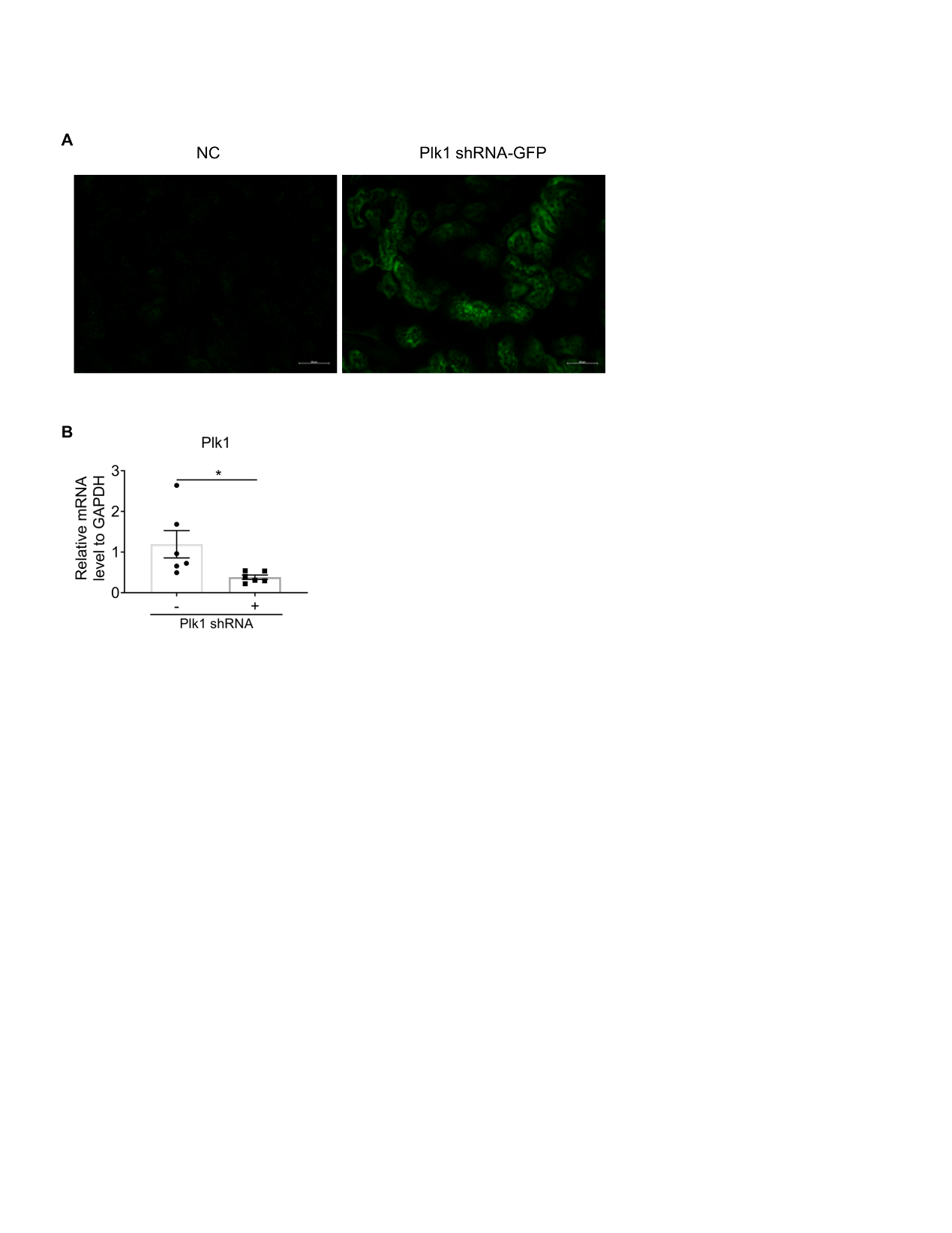


**Supplementary Figure 4**

**Fig. S4**


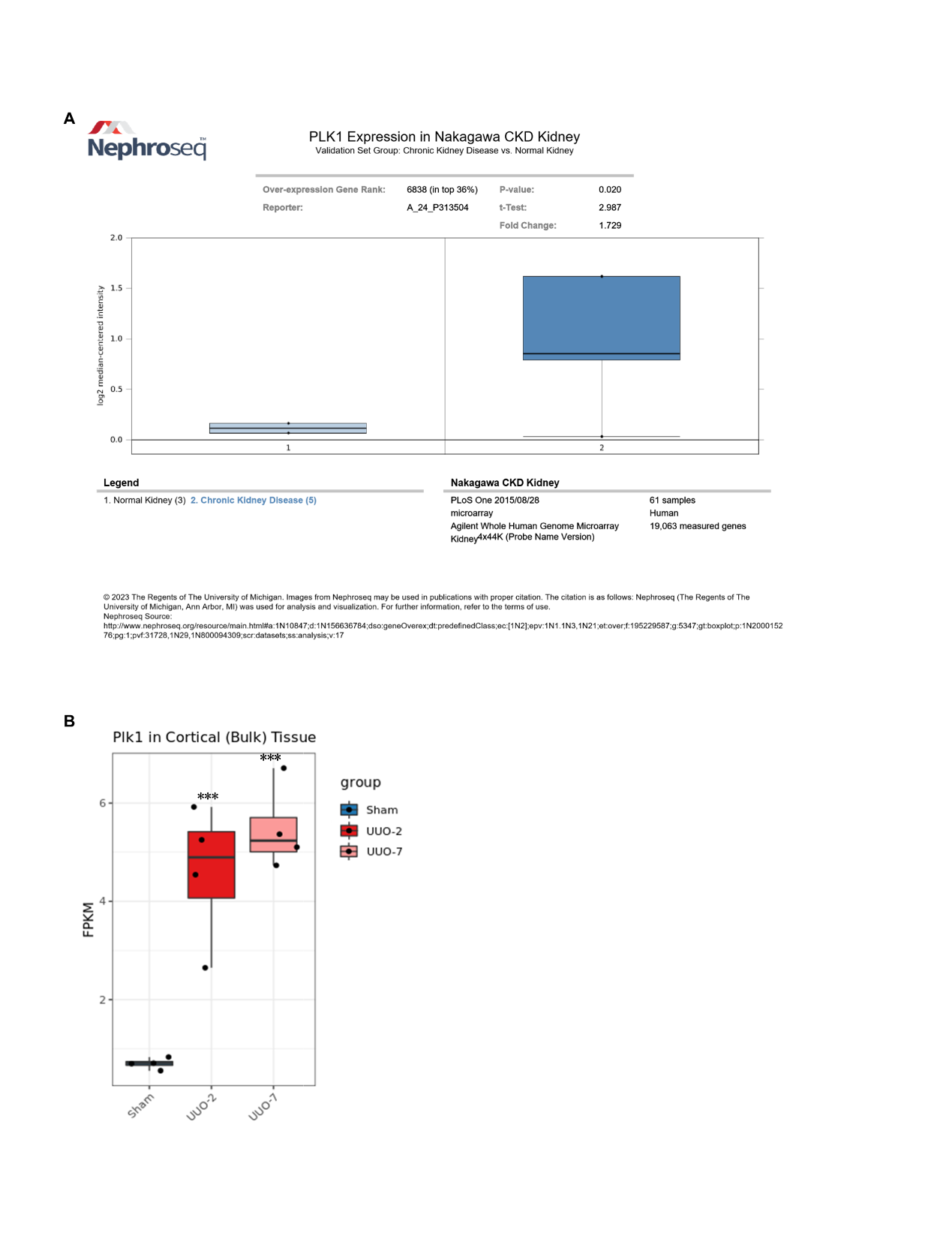


**Supplementary Figure 5**

**Fig. S5**


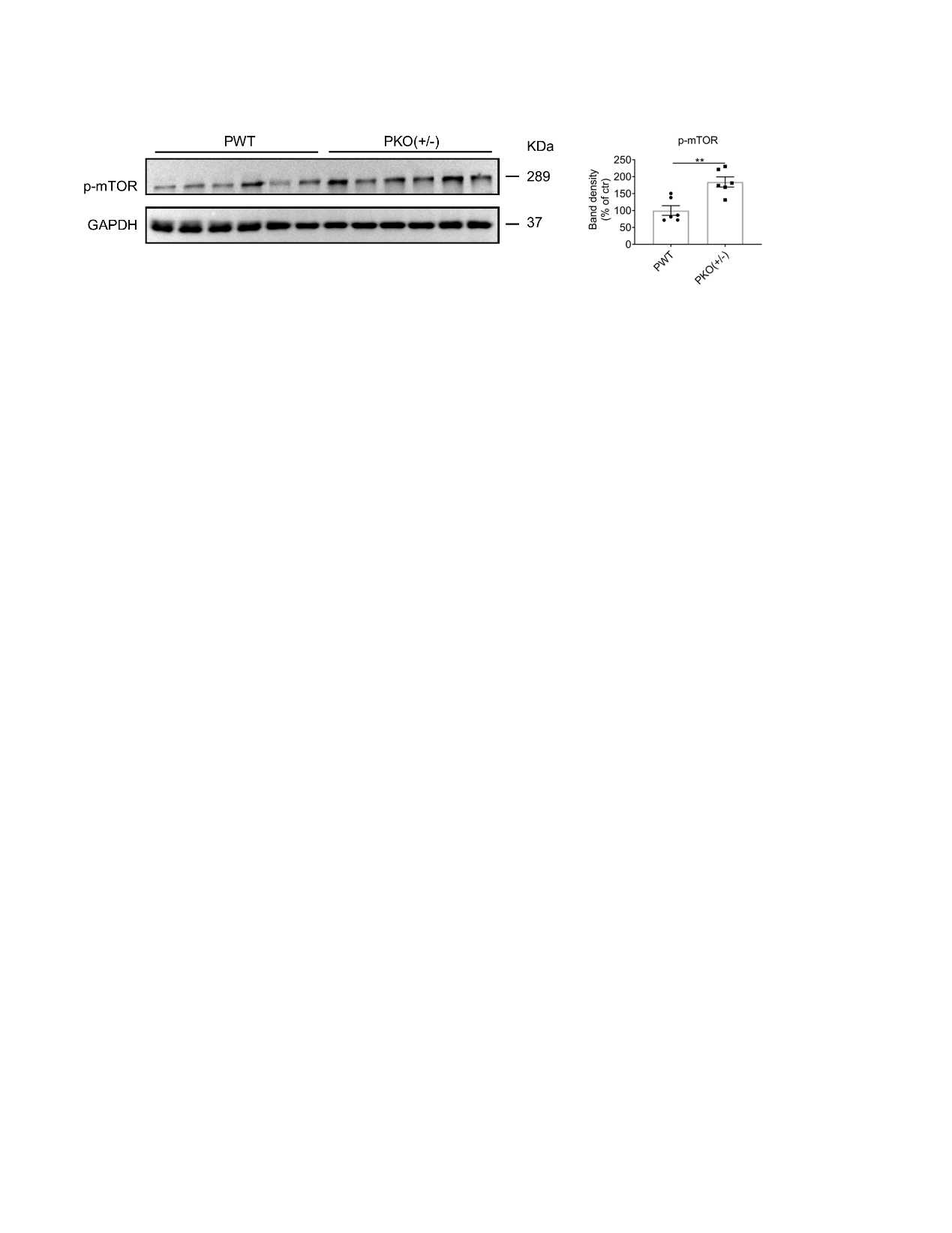


**Supplementary Figure 6**

**Fig. S6**


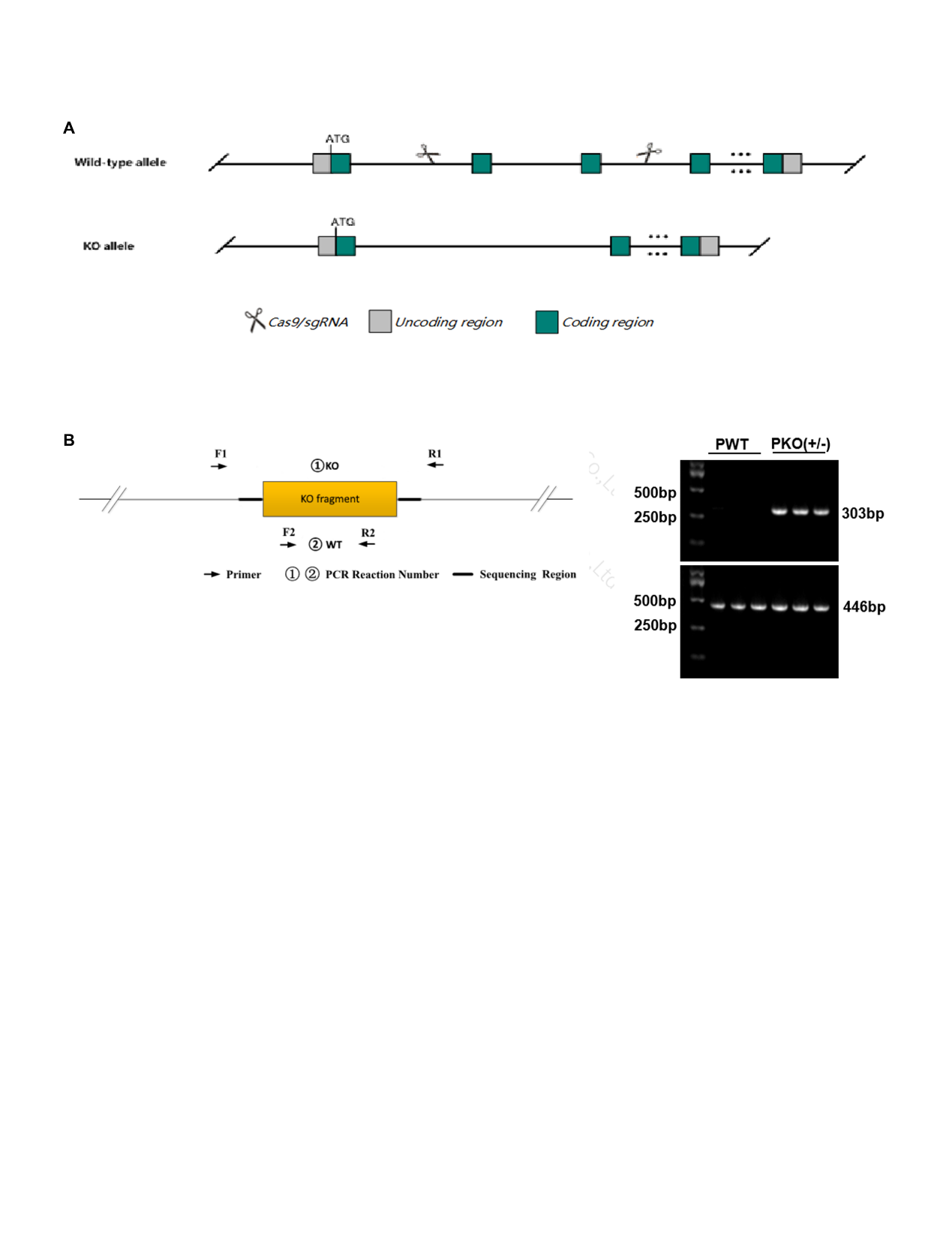


**Supplementary Figure 7**

**Fig. S7**


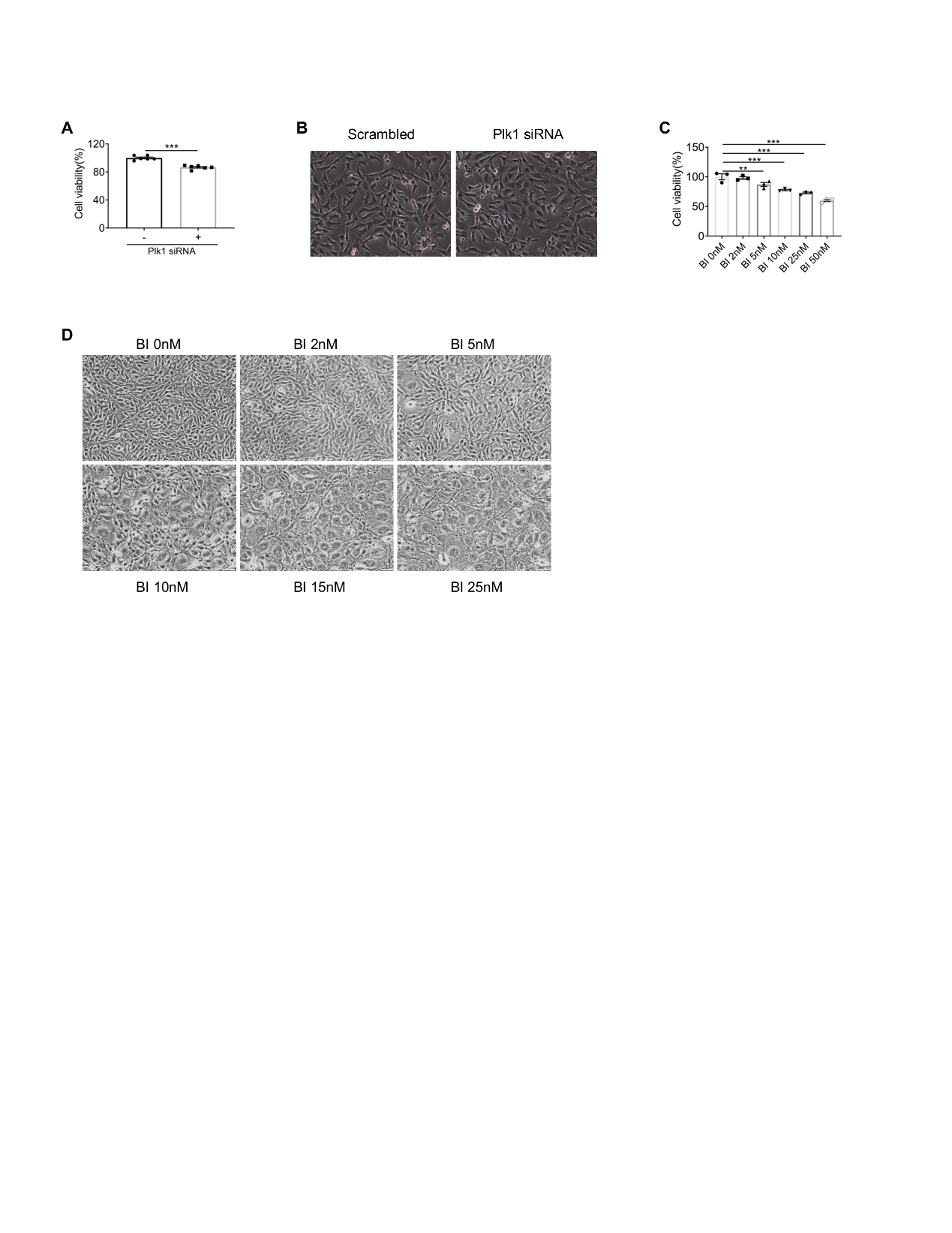

Supplement: Supplementary file 1 — Supplementary Figure [file 41419_2023_6093_MOESM1_ESM.docx]
