# Supplementary material for: Plk1 promotes renal tubulointerstitial fibrosis by targeting autophagy/lysosome axis
Source: Cell Death Dis. 2023 Aug 29;14(8):571. doi: 10.1038/s41419-023-06093-4 (PMC10462727; doi:10.1038/s41419-023-06093-4)
Supplement: Supplementary file 2 — Supplementary Figure legend [file 41419_2023_6093_MOESM2_ESM.docx]

**Supplemental Figure legend**

**Figure S1 Inhibition of Plk1 alleviates kidney inflammation in UUO mice.** (**A**) Western blot analysis of Plk1 expression in kidneys of sham and UUO mice with or without Plk1 silencing. n=3. The GAPDH for Plk1 was from same experiment, same samples and processed in parallel. (**B**) qRT-PCR analysis of MCP1, IL-1β, and IL-6 in kidneys of sham and UUO mice with or without Plk1 silencing. n=6. (**C**) Western blot analysis of p-NF-κB p65 expression and (**D**) qRT-PCR analysis of MCP1 and IL-1β in kidneys of sham and UUO mice treated or not with BI6727. n=6. **P* < 0.05, ***P* < 0.01, ****P* < 0.001.

**Figure S2 Transmission electron microscope images of autolysosomes in NRK49F cells.** (**A**) Representative image of autolysosomes in control and Plk1 knockdown cells. (**B**) Representative image of autolysosomes in control and BI6727-treated cells(15nM). Scale bar=2µm, 500nm. n=3.

**Figure S3 The efficiency of Plk1 knockdown in vivo by hydrodynamic-based gene delivery approach.** (**A**) The GFP fluorescence confirmed the transfection efficiency. Scale bar=50µm. (**B**) qRT-PCR analysis of kidney Plk1 in mice with or without Plk1 knockdown. n=6. **P* < 0.05, ***P* < 0.01, ****P* < 0.001.

**Figure S4 Transcriptomic database–based analysis of Plk1 expression in kidneys of CKD patients and animal model.** (**A**) Nephroseq (<https://nephroseq.com/resource/login.html>) database analysis of Plk1 expression in CKD patients kidney compared to normal kidney. (**B**) Gene Atlas of Reversible Unilateral Ureteric Obstruction Model (rUUO) (<http://www.ruuo-kidney-gene-atlas.com/>) database analysis showed Plk1 expression in kidneys of UUO mice was significantly higher compared to sham kidney.

**Figure S5 p-mTOR expression in hyterozygous Plk1 knockout mice.** Western blot analysis of p-mTOR in contralateral kidneys of PWT and PKO**+/-** mice with UUO, and densitometry analysis of the bands. n=6. **P* < 0.05, ***P* < 0.01, ****P* < 0.001.

**Figure S6 Gene editing and genotyping strategies for Plk1 knockout mice.** (**A**) Gene editing strategy for generation of Plk1 knockout mice. (**B**) Genotyping strategy and results for PWT and PKO**+/-** mice.

**Figure S7 The impact of Plk1 inhibition on Cell viability and cell morphology change in NRK49F cells.** NRK49F cells were transfected with Plk1 siRNA for 24–48 h. (**A**) Cell viability was detected by CCK8 and (**B**) Cell morphology was observed under light microscope. Scale bar=50 µm. Then, NRK49F cells were treated with BI6727, (**C**) Cell viability was analysed by CCK8 and (**D**) Cell morphology was observed under the light microscope. Scale bar=50 µm. n=3. **P* < 0.05, ***P* < 0.01, ****P* < 0.001.
