# Supplementary material for: Plk1 promotes renal tubulointerstitial fibrosis by targeting autophagy/lysosome axis
Source: Cell Death Dis. 2023 Aug 29;14(8):571. doi: 10.1038/s41419-023-06093-4 (PMC10462727; doi:10.1038/s41419-023-06093-4)
Supplement: Supplementary file 4 — western blot original data [file 41419_2023_6093_MOESM4_ESM.pdf]

Figure1:

**E**

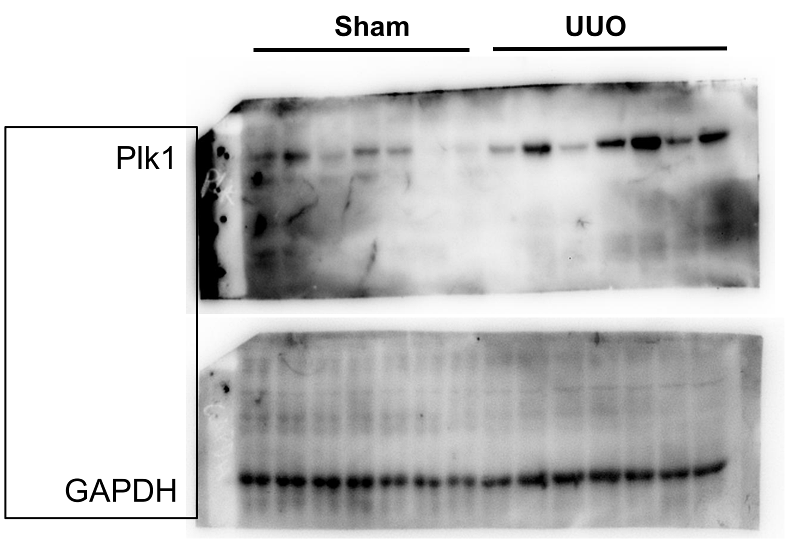


Figure2:

C

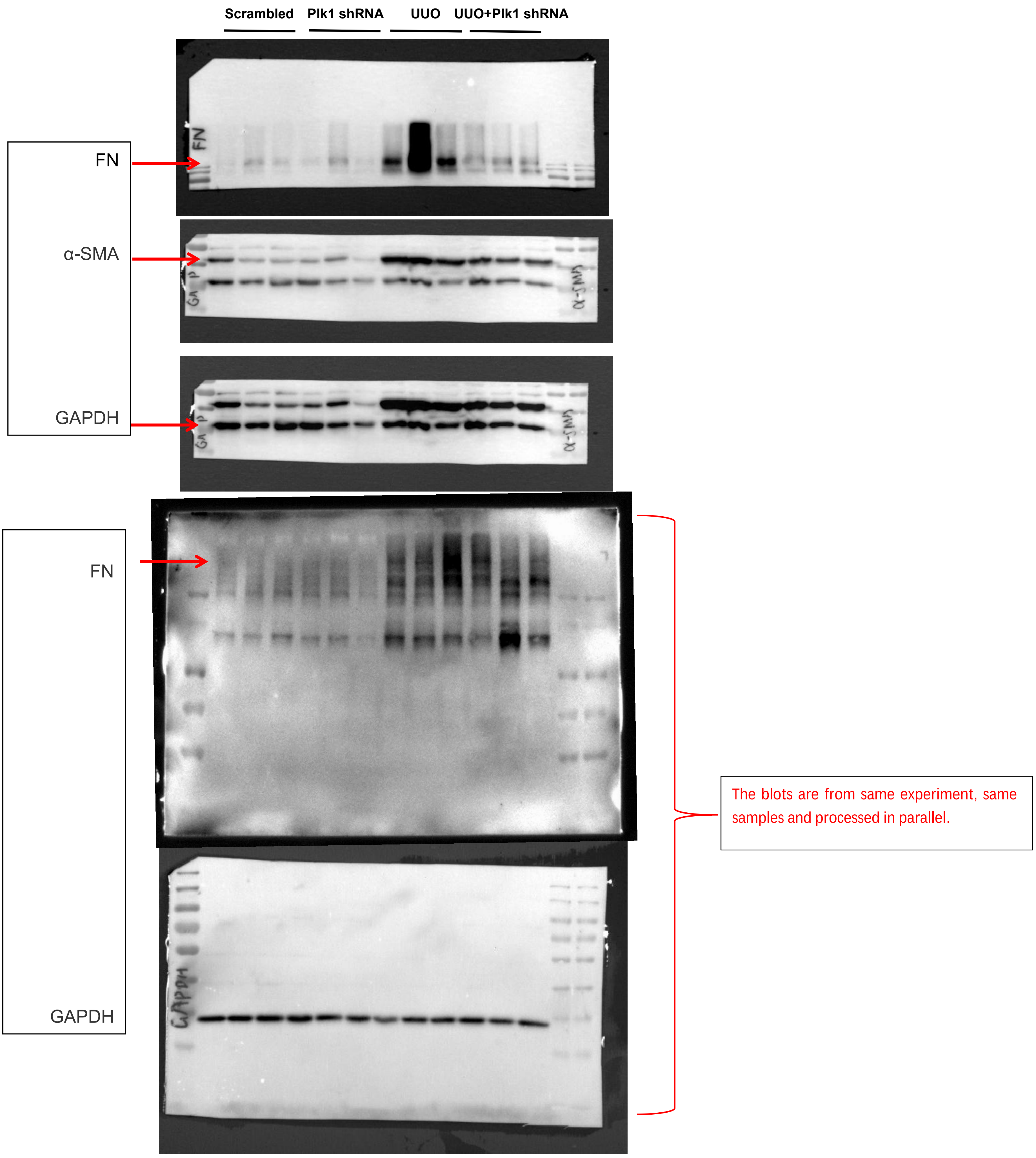

α-SMA

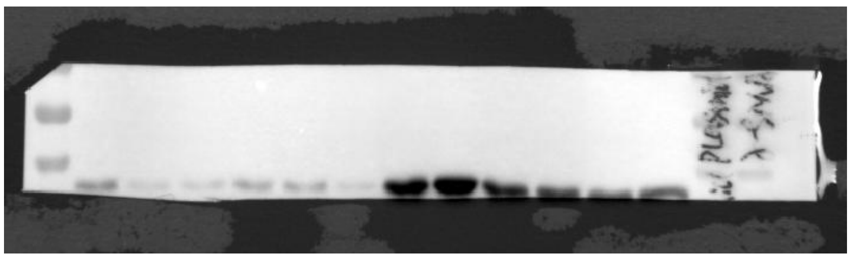

GAPDH

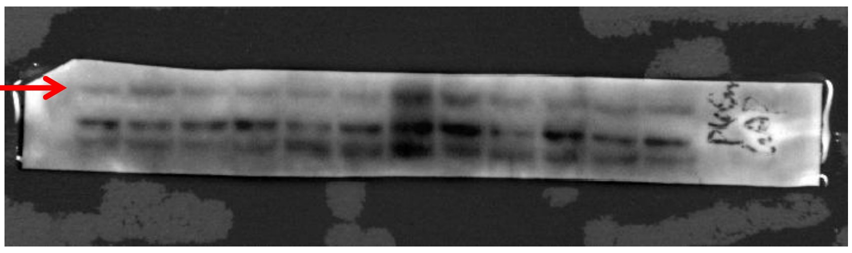

CollagenIII

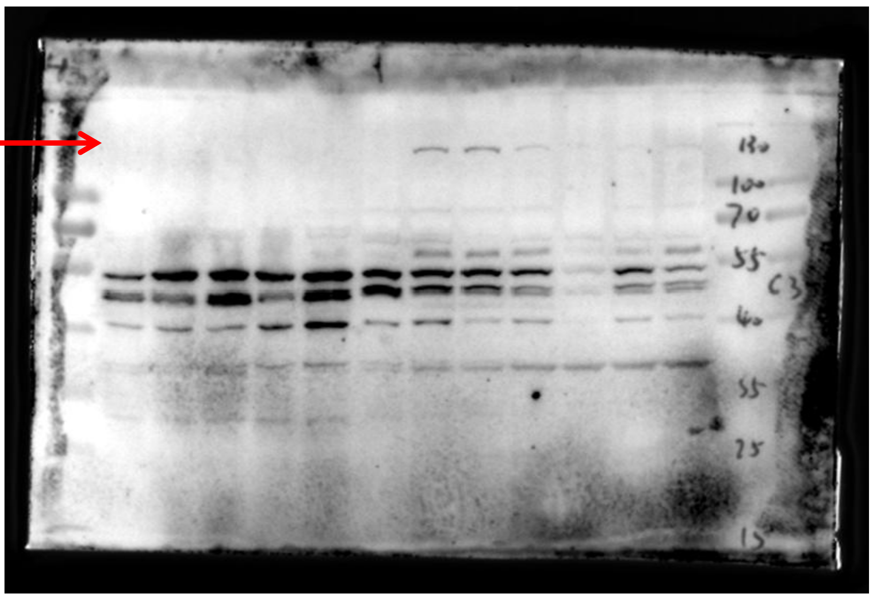

GAPDH

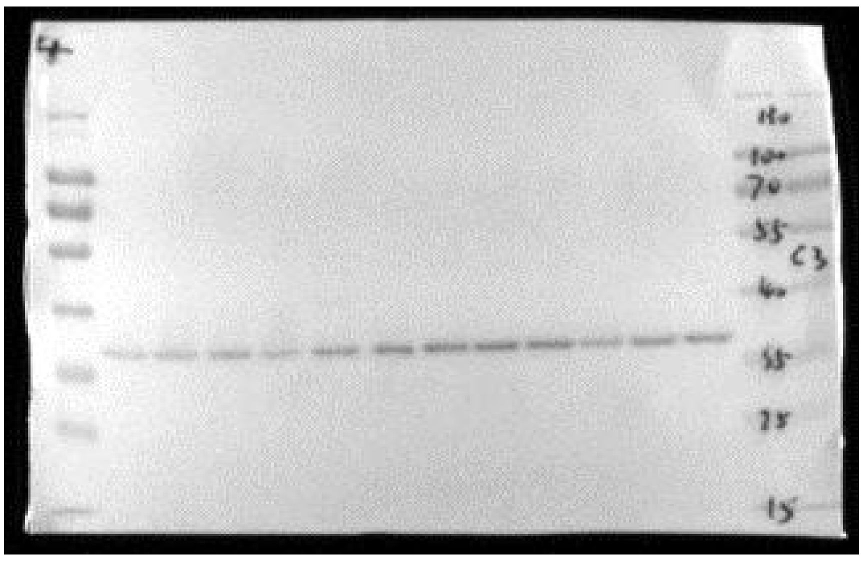

CollagenIII

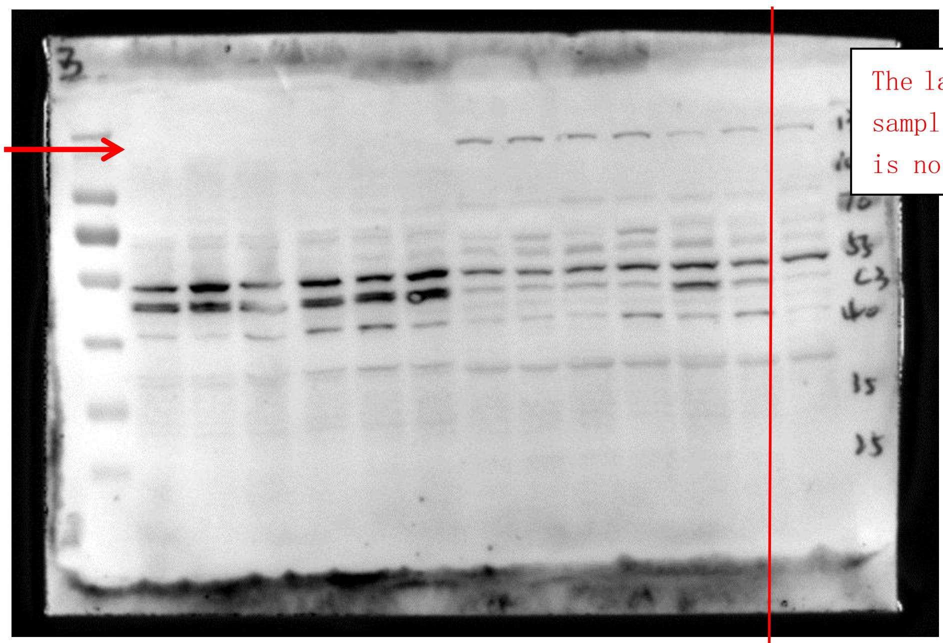

The last well is loaded with other kidney sample just for balancing of the gel. It is not included for analysis.

GAPDH

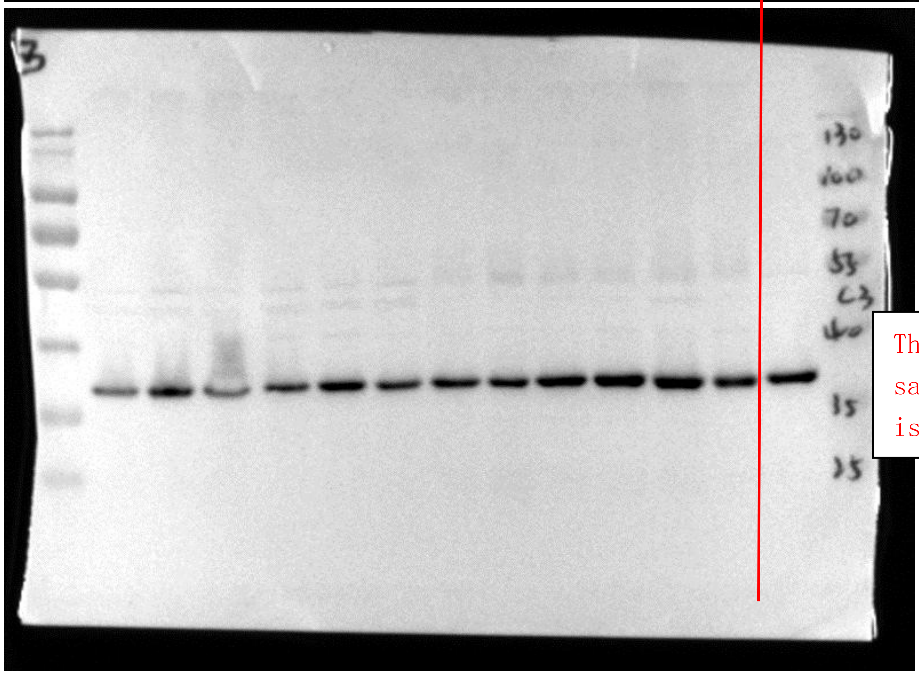

The last well is loaded with other kidney sample just for balancing of the gel. It is not included for analysis.

Figure3: C

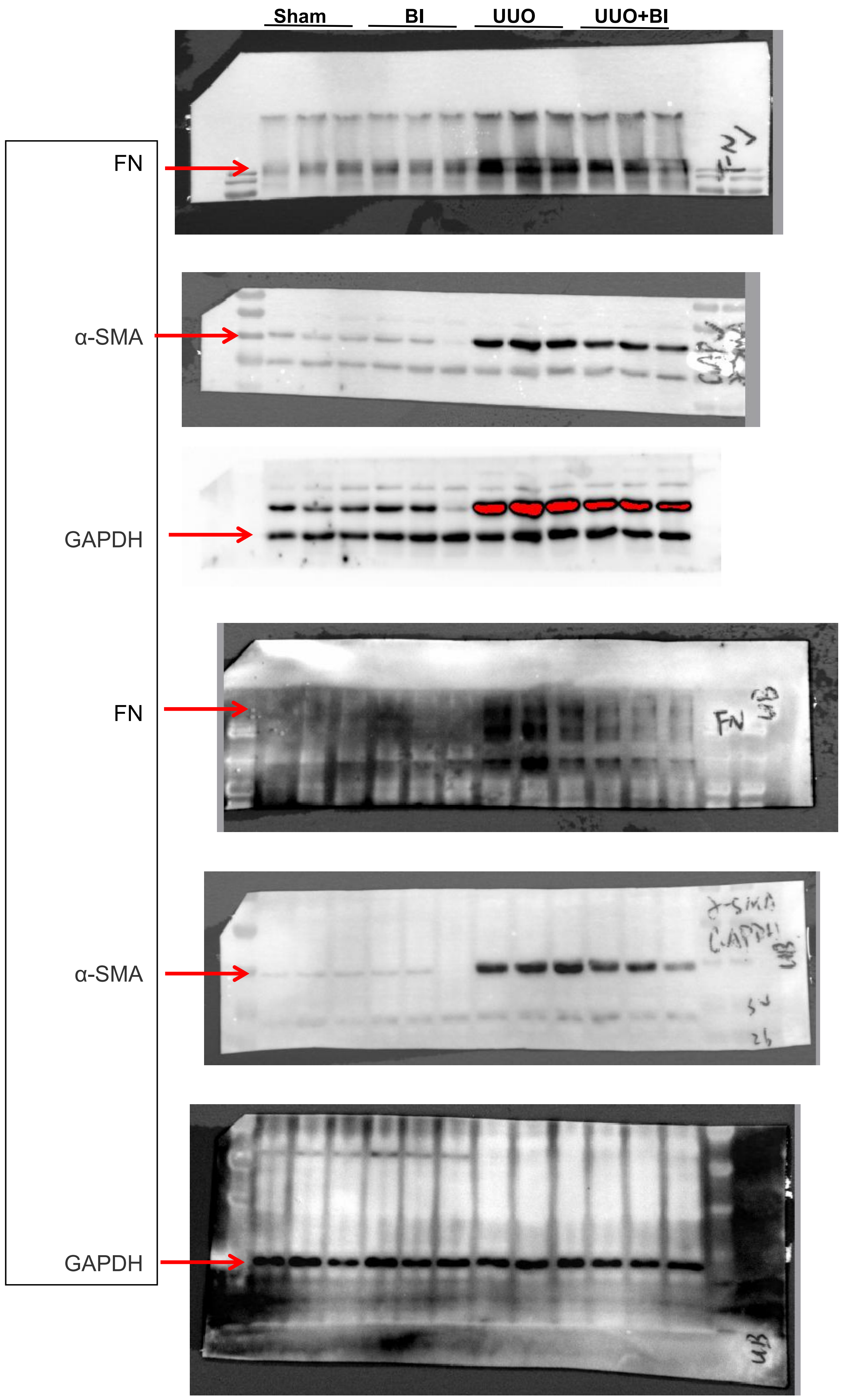

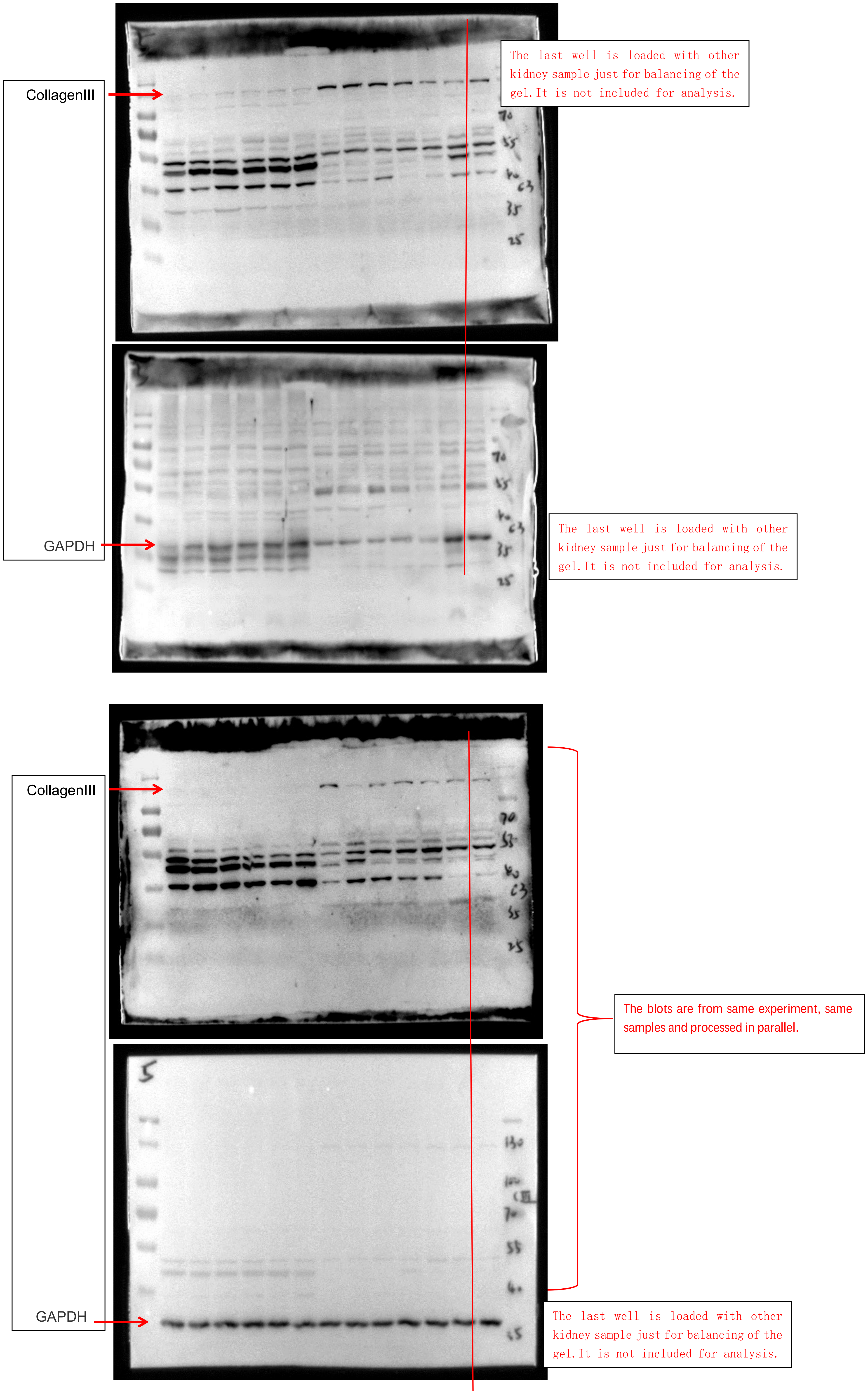
CollagenIII
GAPDH
The last well is loaded with other kidney sample just for balancing of the gel. It is not included for analysis.
The last well is loaded with other kidney sample just for balancing of the gel. It is not included for analysis.
CollagenIII
GAPDH
The blots are from same experiment, same samples and processed in parallel.
The last well is loaded with other kidney sample just for balancing of the gel. It is not included for analysis.

Figure4: C

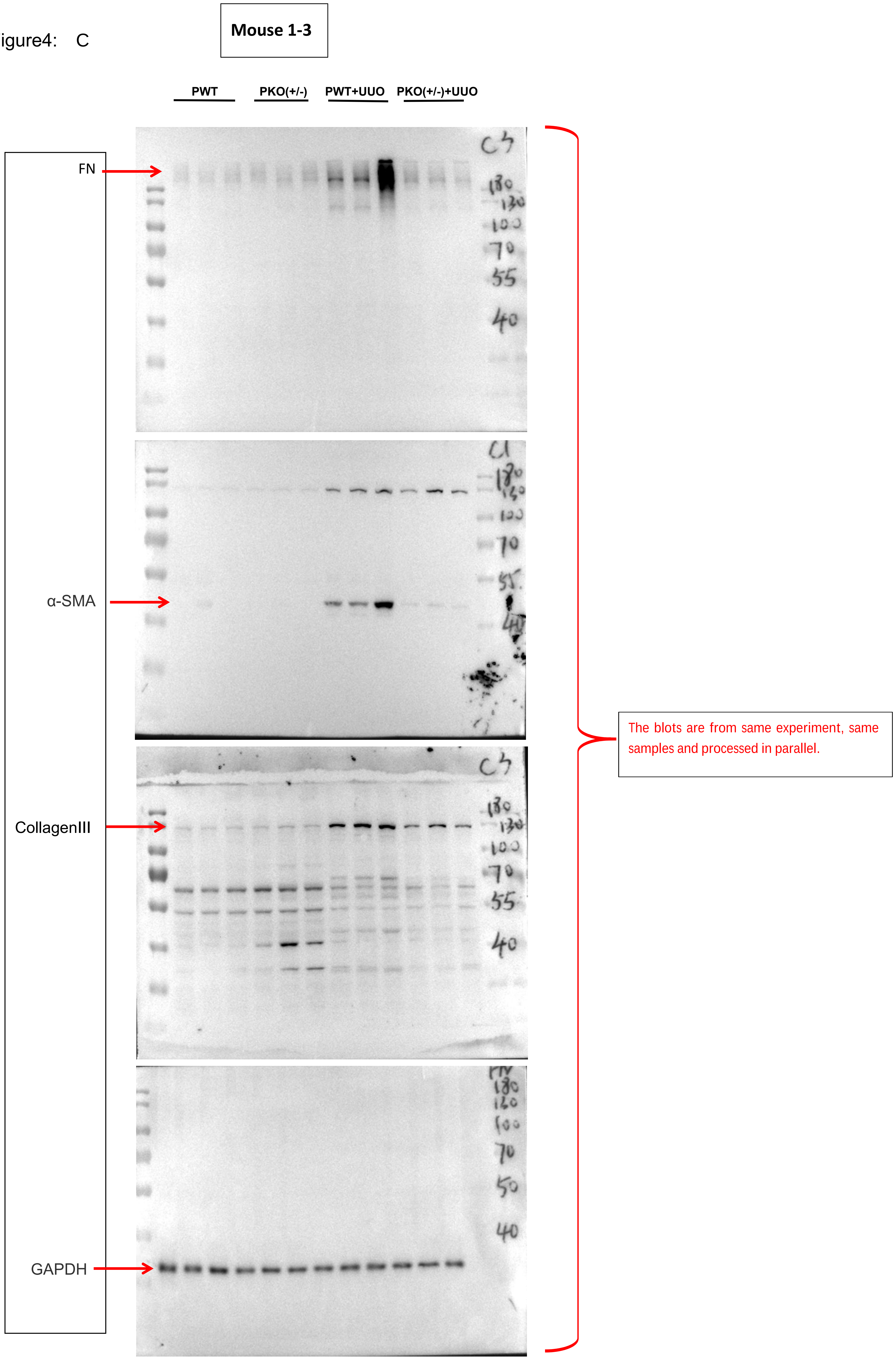
Mouse 1-3
PWT
PKO(+/-)
PWT+UUO
PKO(+/-)+UUO
FN
α-SMA
CollagenIII
GAPDH
180
130
100
70
55
40
The blots are from same experiment, same samples and processed in parallel.

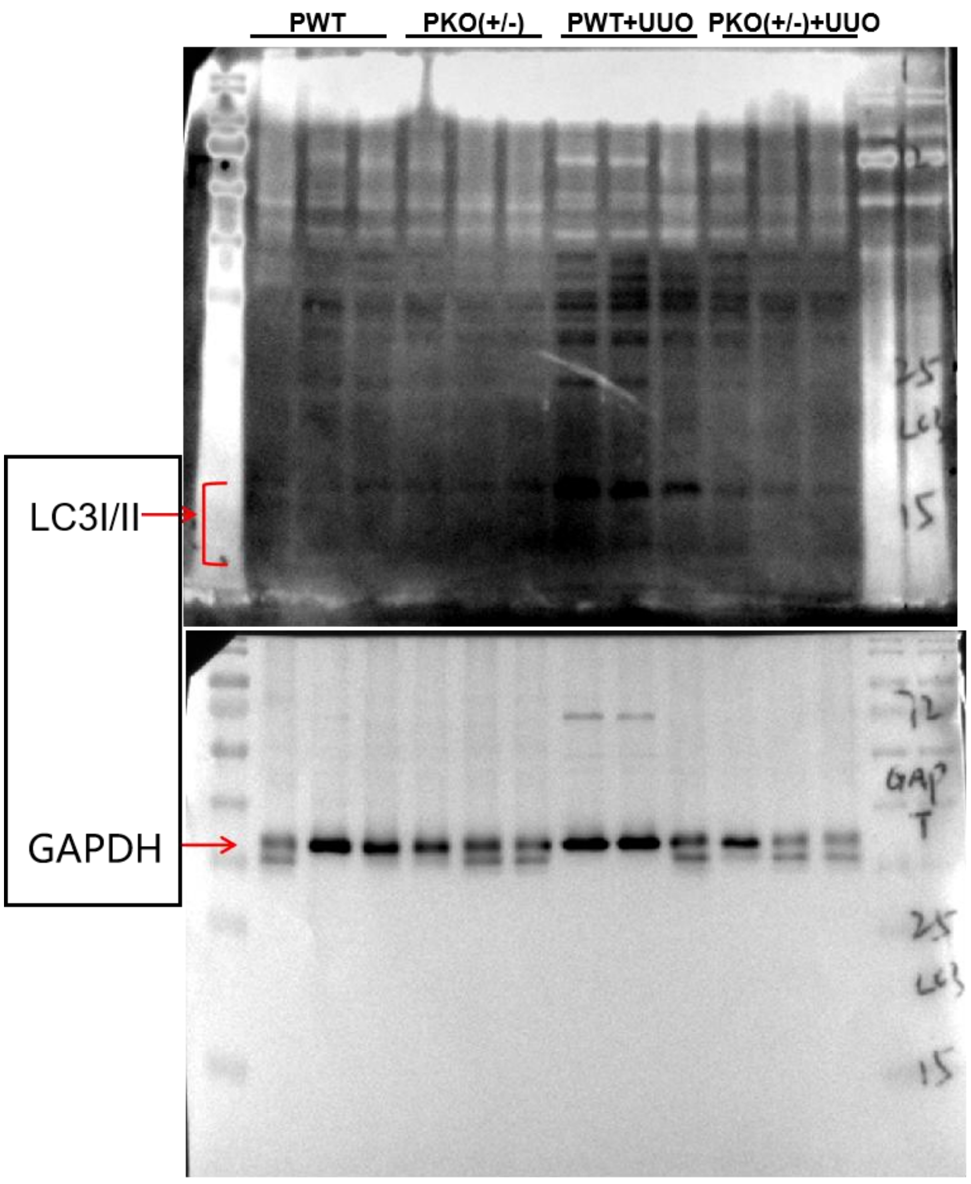
PWT
PKO(+/-)
PWT+UUO
PKO(+/-)+UUO
LC3I/II
GAPDH

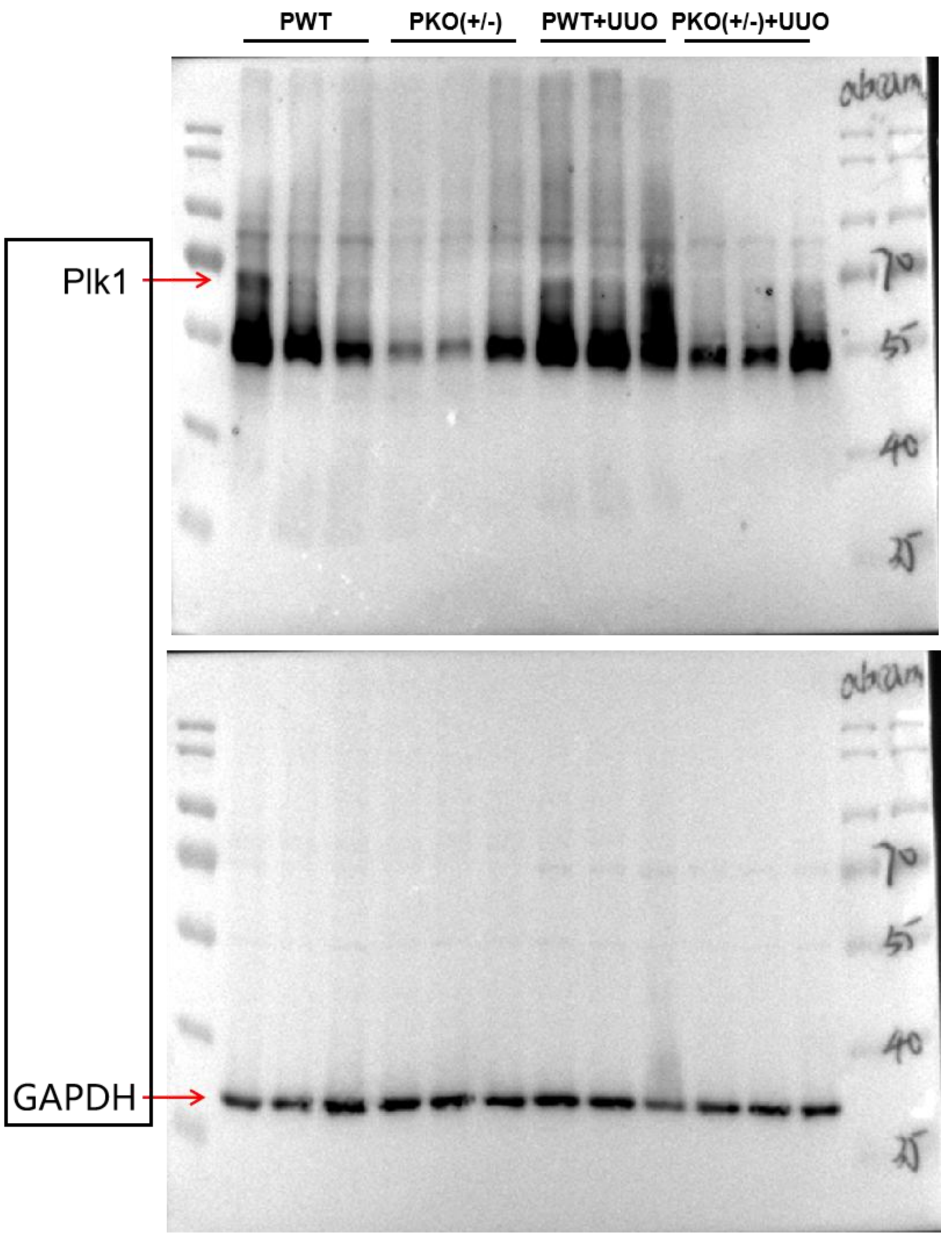
PWT
PKO(+/-)
PWT+UUO
PKO(+/-)+UUO
Plk1
GAPDH

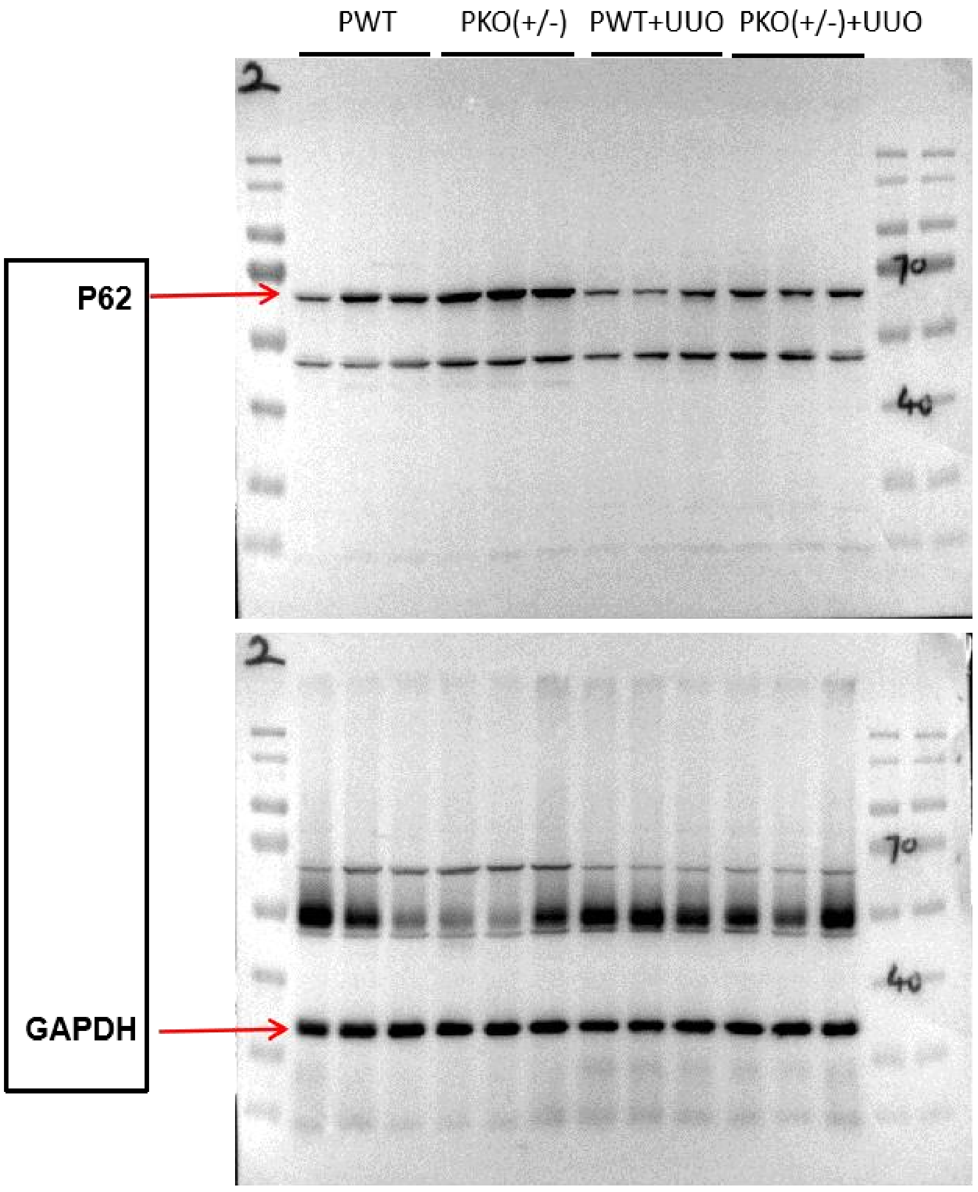
PWT
PKO(+/-)
PWT+UUO
PKO(+/-)+UUO
P62
GAPDH
70
40

**Mouse 4-6**

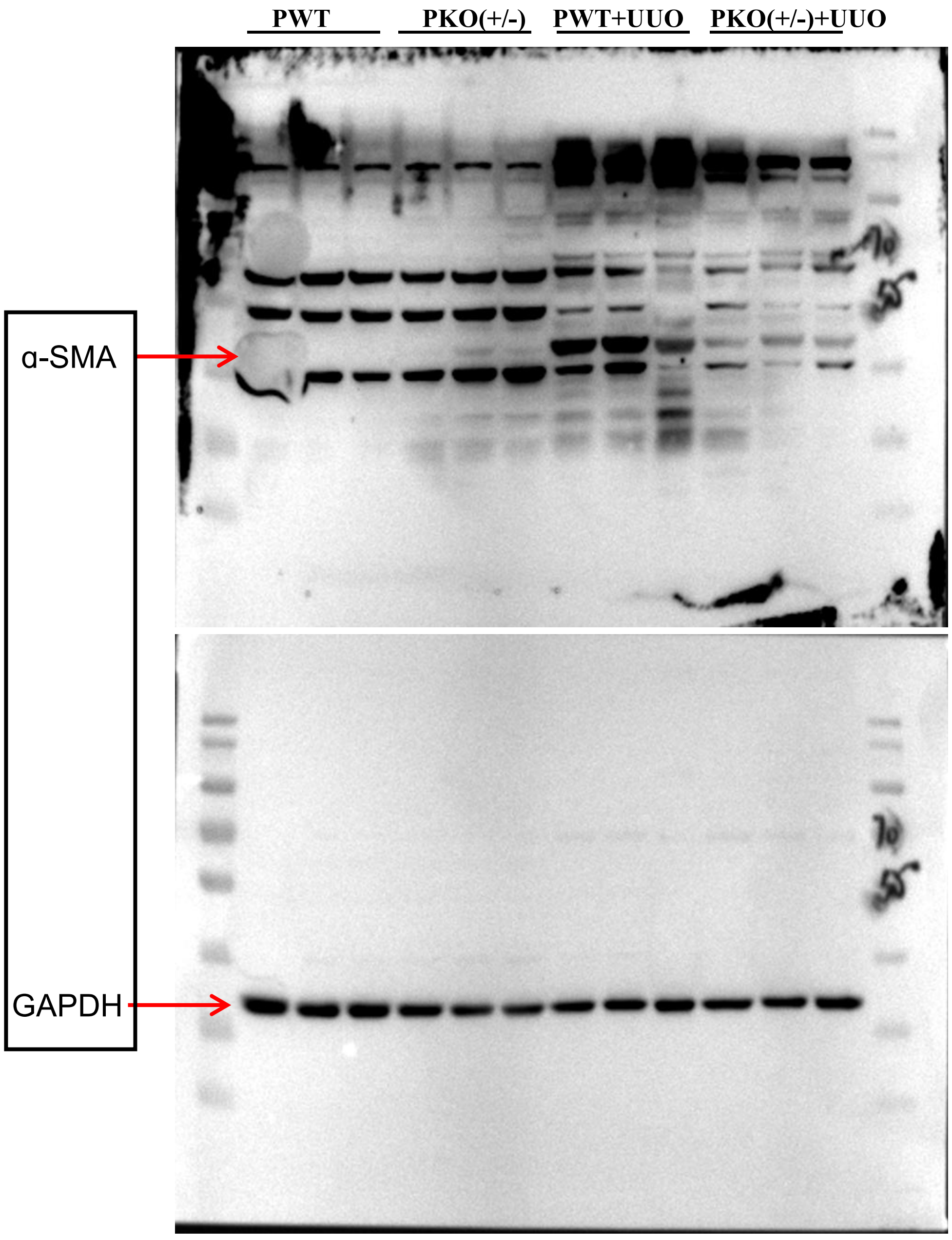
PWT
PKO(+/-)
PWT+UUO
PKO(+/-)+UUO
α-SMA
GAPDH

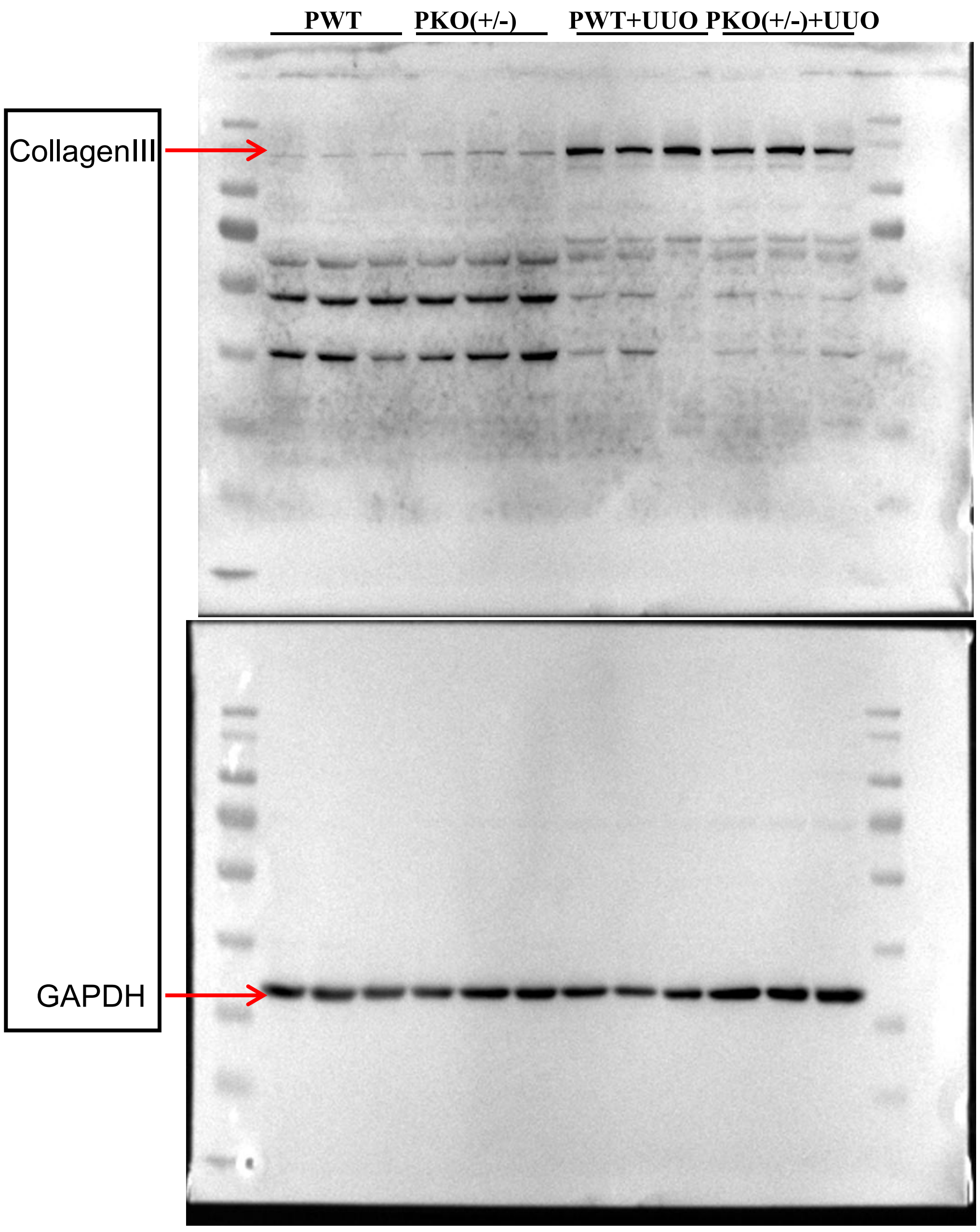
PWT
PKO(+/-)
PWT+UUO
PKO(+/-)+UUO
CollagenIII
GAPDH

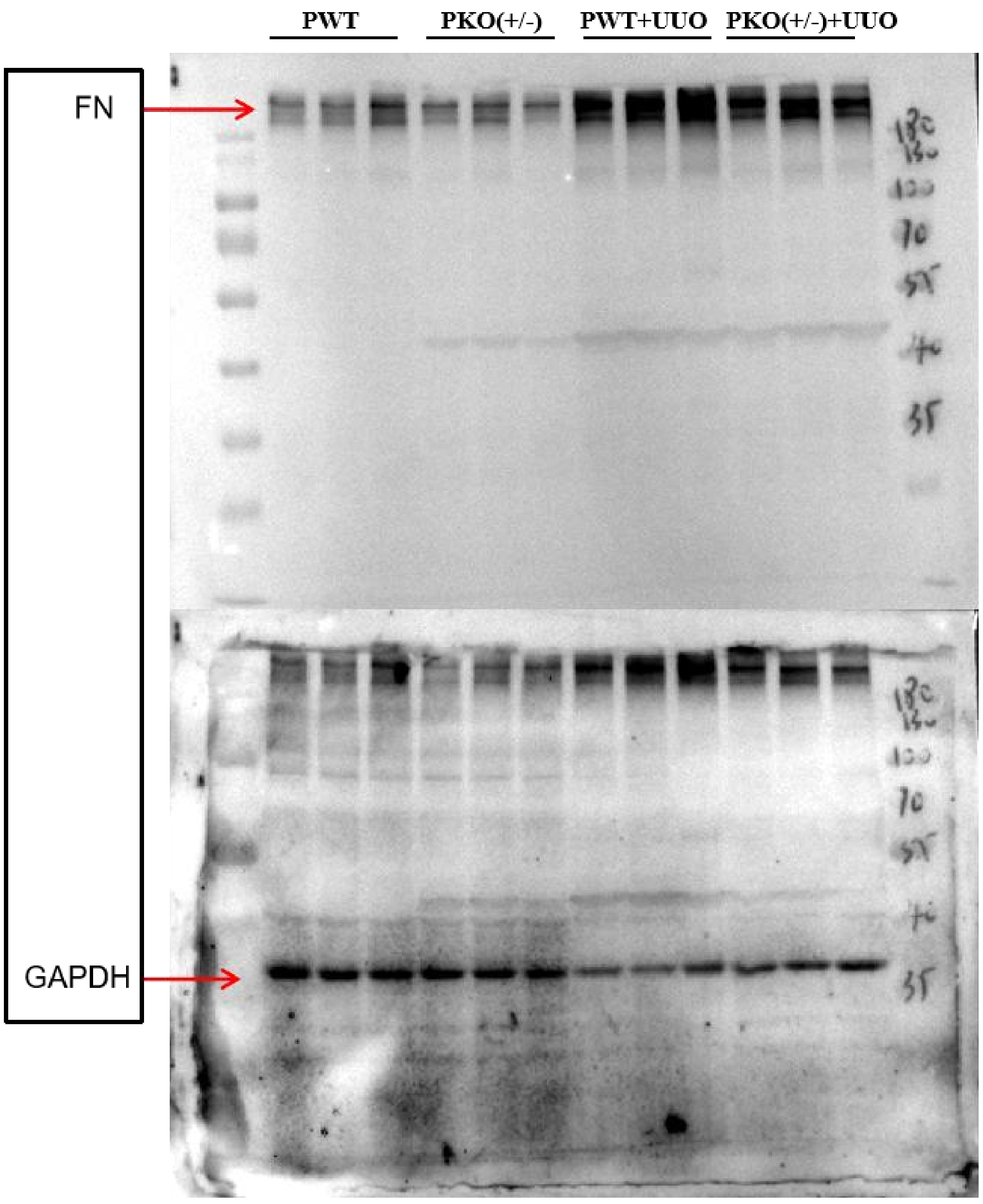
PWT
PKO(+/-)
PWT+UUO
PKO(+/-)+UUO
FN
GAPDH

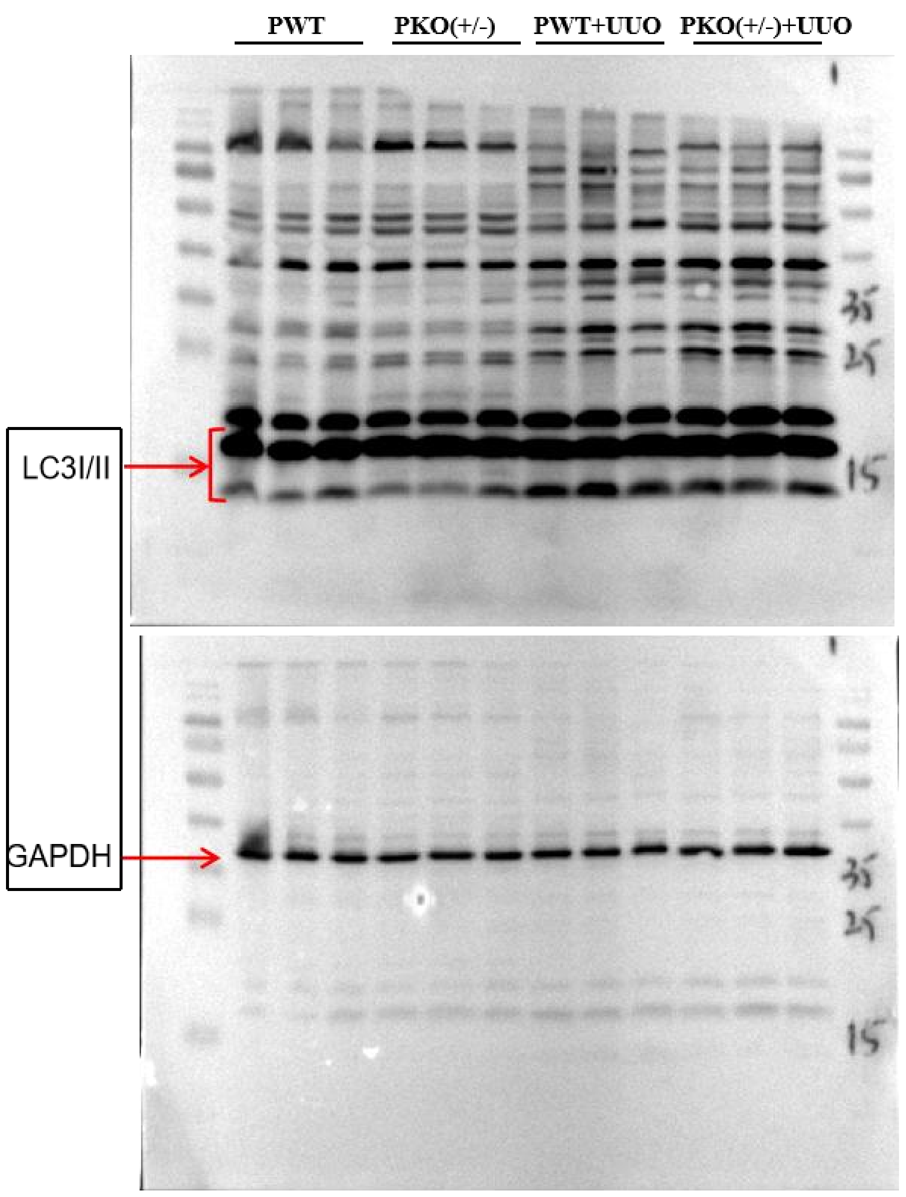
PWT
PKO(+/-)
PWT+UUO
PKO(+/-)+UUO
LC3I/II
GAPDH
35
25
15
35
25
15

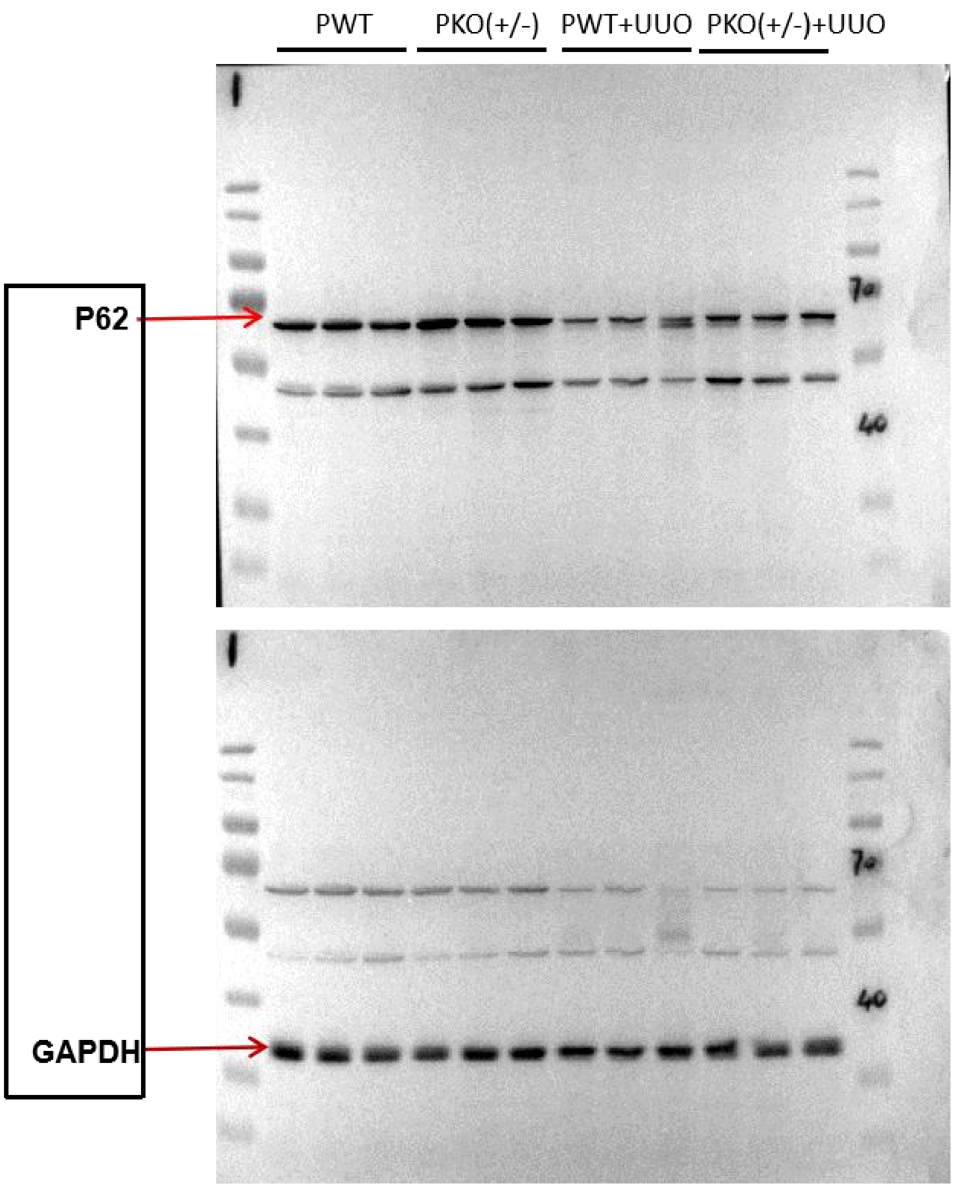
PWT
PKO(+/-)
PWT+UUO
PKO(+/-)+UUO
P62
70
40
GAPDH
70
40

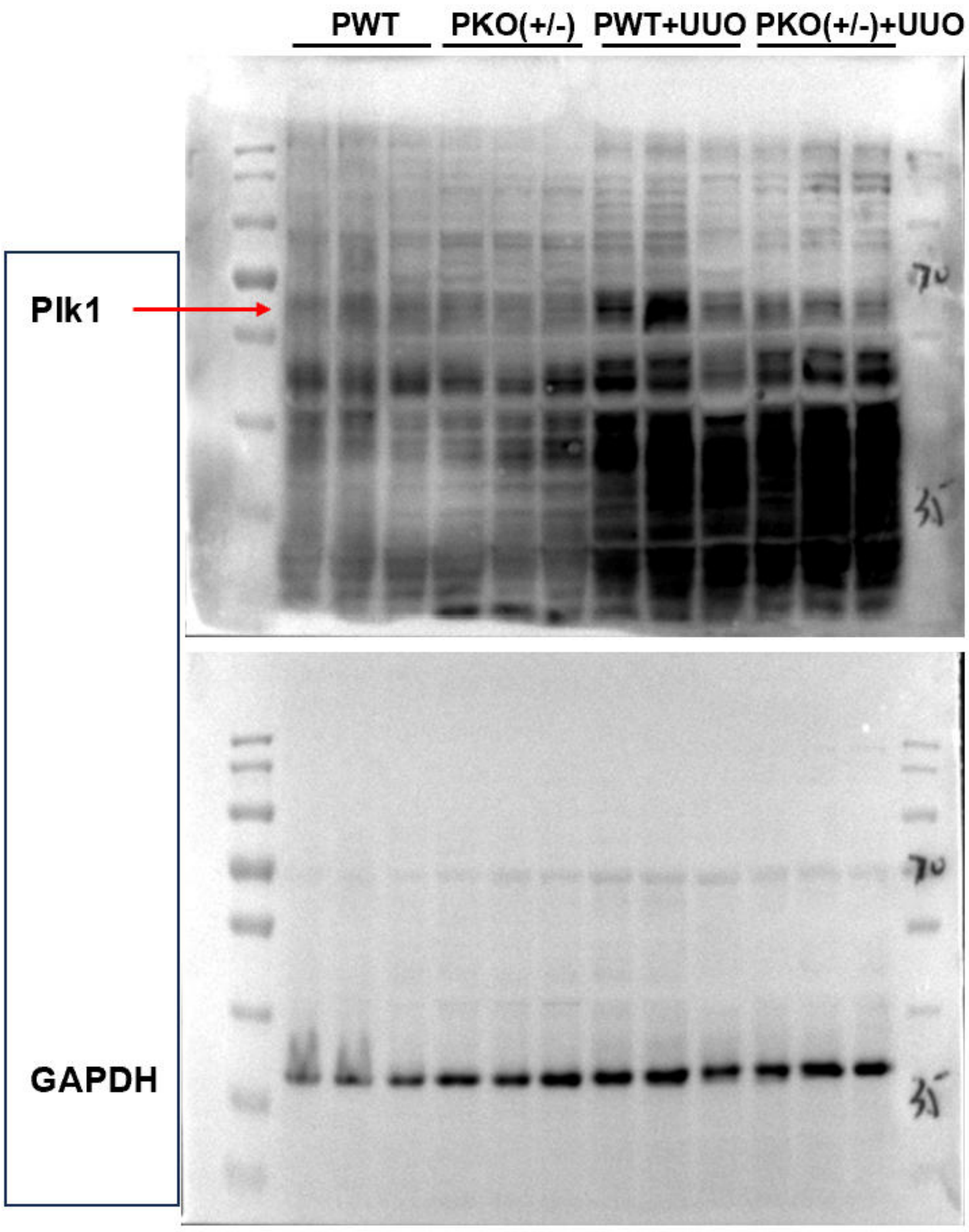
PWT
PKO(+/-)
PWT+UUO
PKO(+/-)+UUO
Plk1
GAPDH
70
35

Figure5:

**A**

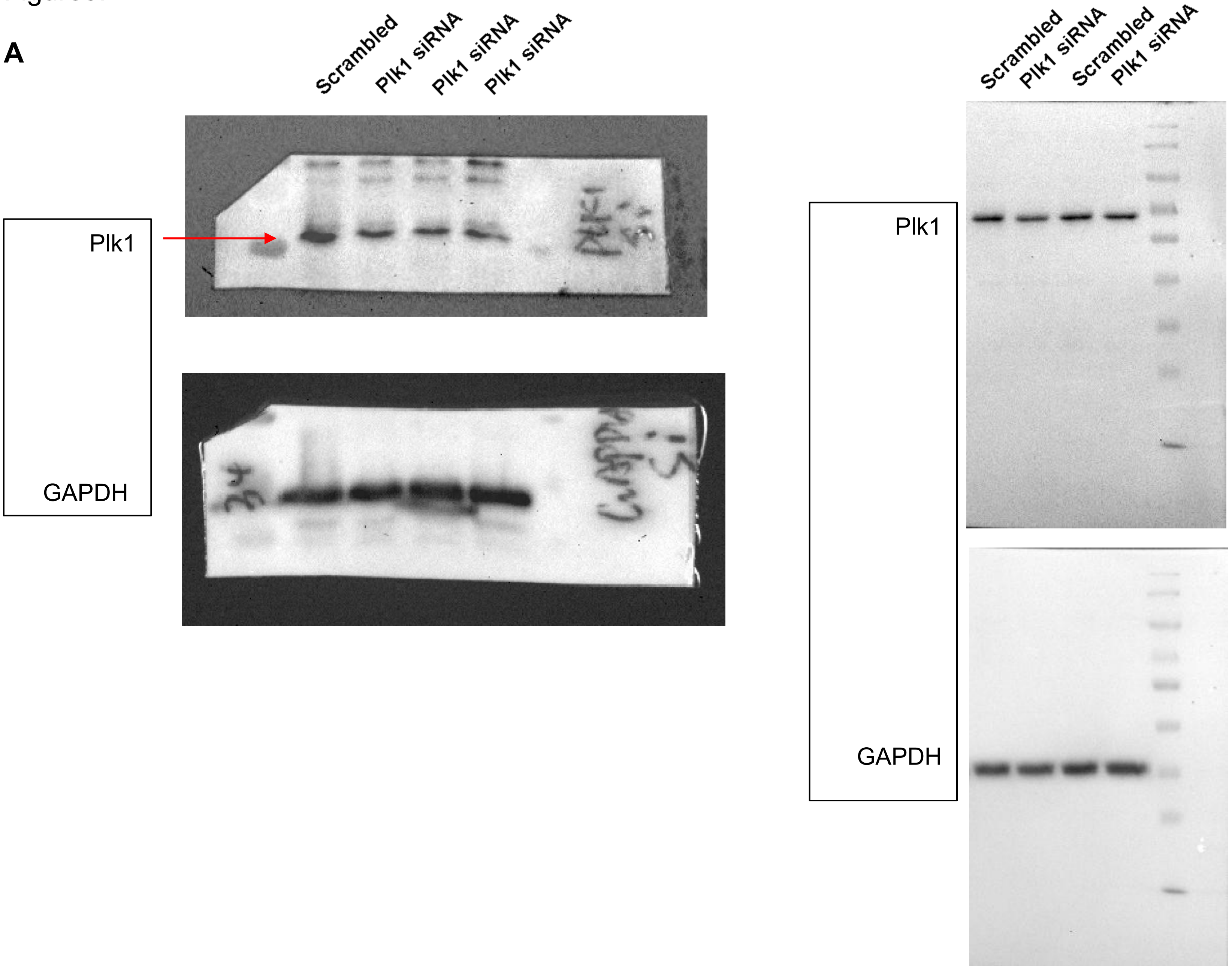
Scrambled
Plk1 siRNA
Plk1 siRNA
Plk1 siRNA
Plk1
GAPDH
Scrambled
Plk1 siRNA
Scrambled
Plk1 siRNA
Plk1
GAPDH

**D**

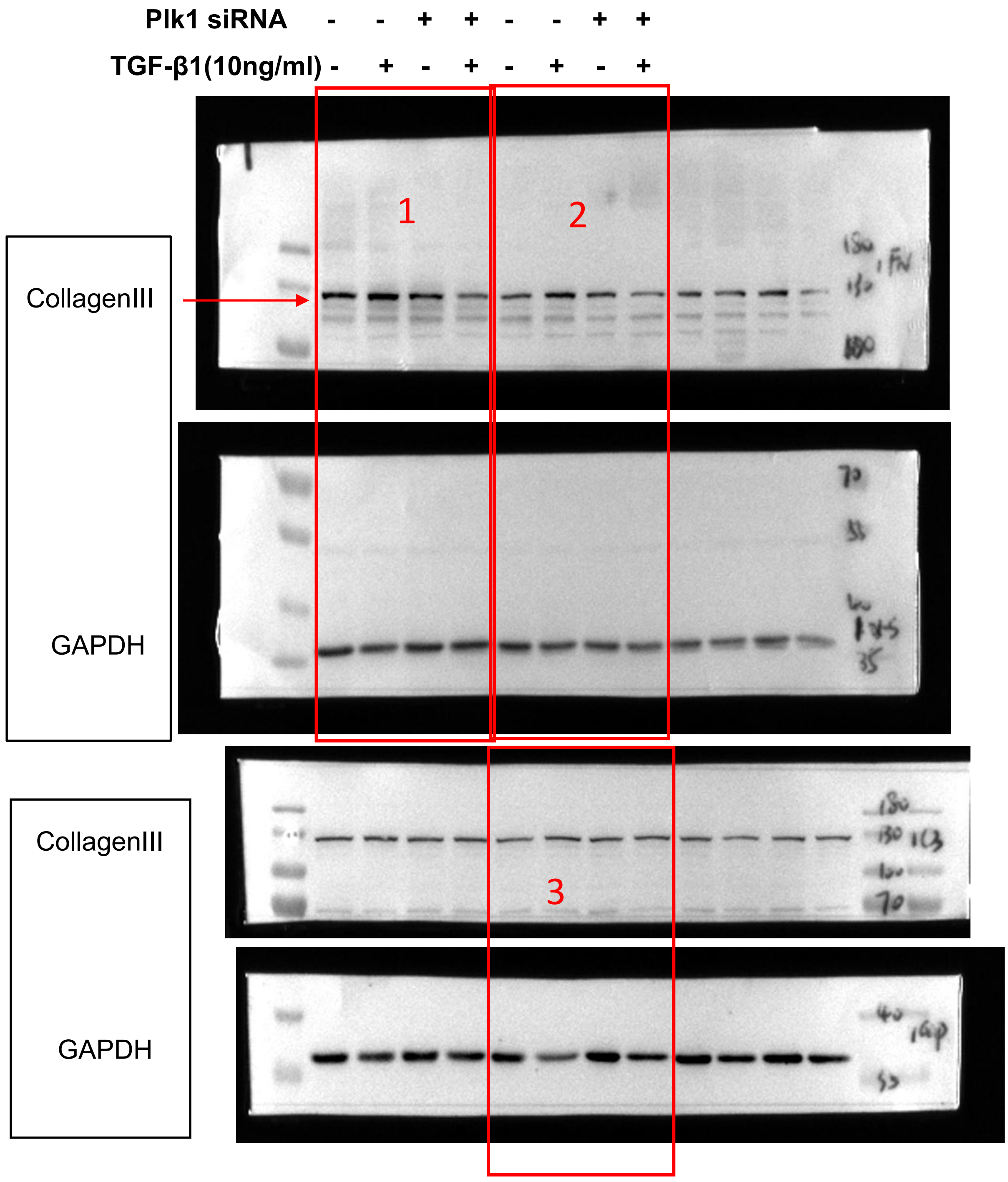
Plk1 siRNA - - + + - - + +
TGF-β1(10ng/ml) - + - + - + - +
1
2
3
CollagenIII
GAPDH
CollagenIII
GAPDH

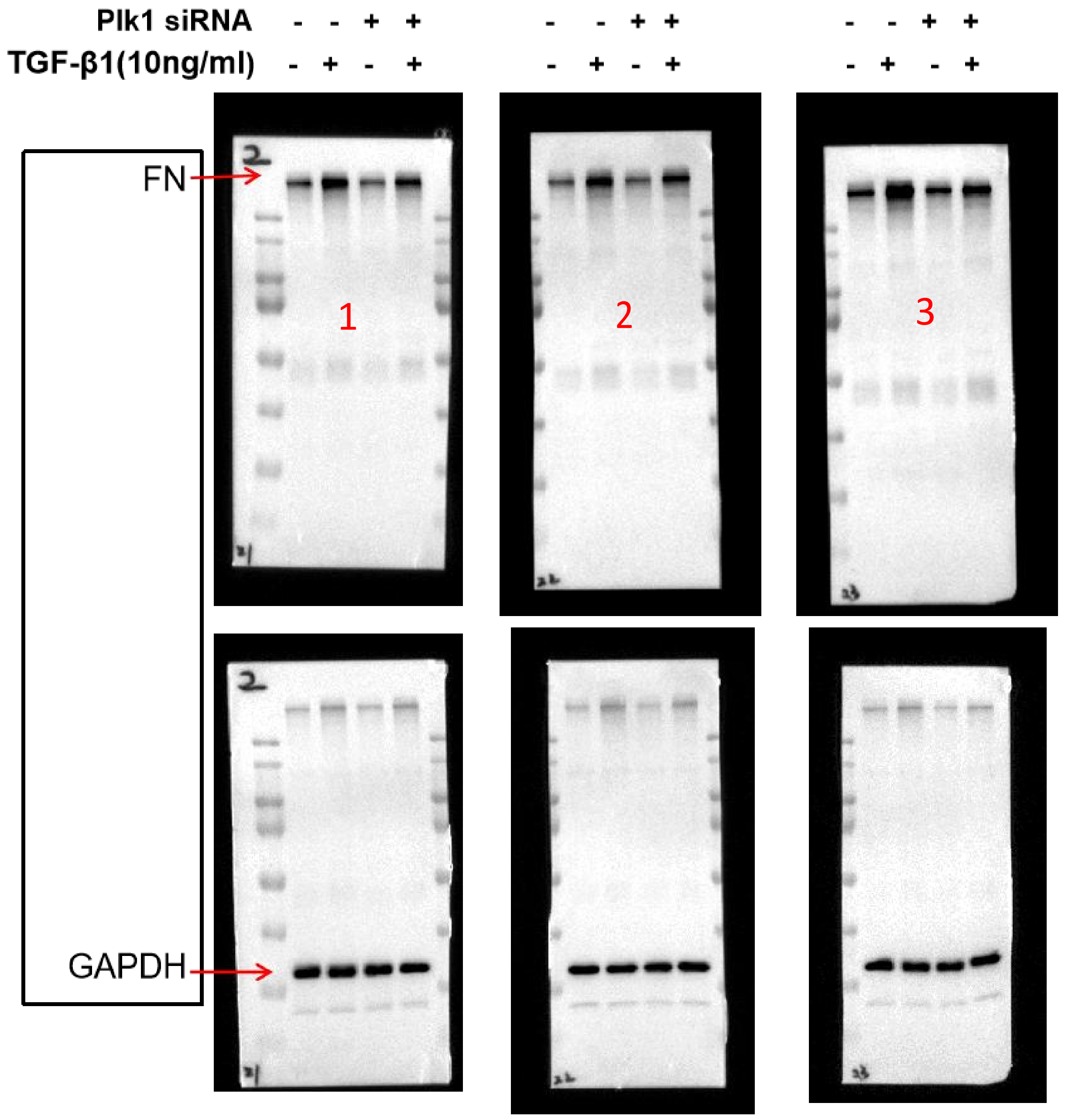
Plk1 siRNA - - + + - - + + - - + +
TGF-β1(10ng/ml) - + - + - + - + - + - +
FN
GAPDH
1
2
3

H

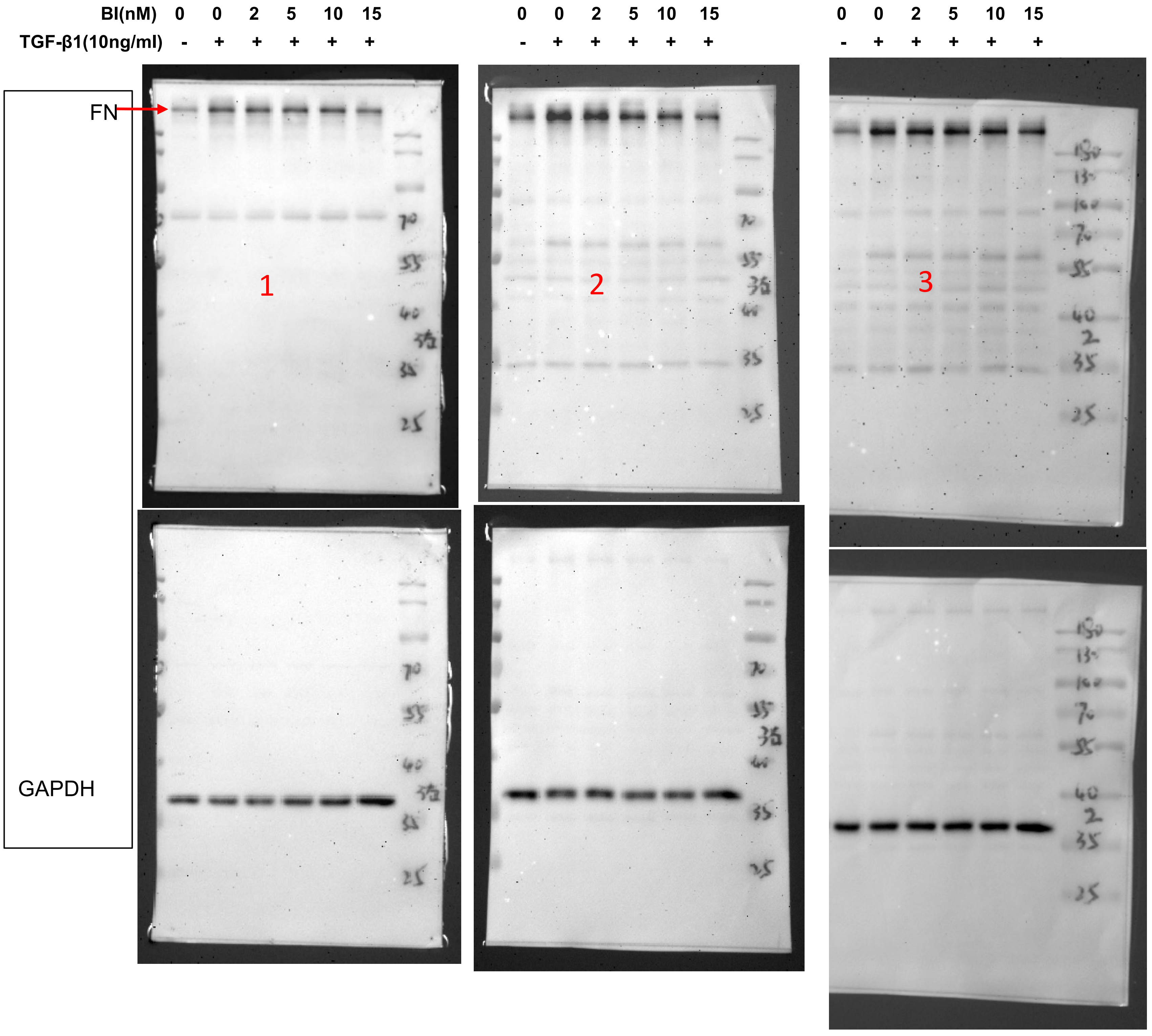
BI(nM) 0 0 2 5 10 15 0 0 2 5 10 15 0 0 2 5 10 15
TGF-β1(10ng/ml) - + + + + + - + + + + + - + + + + +
FN
GAPDH
1
2
3

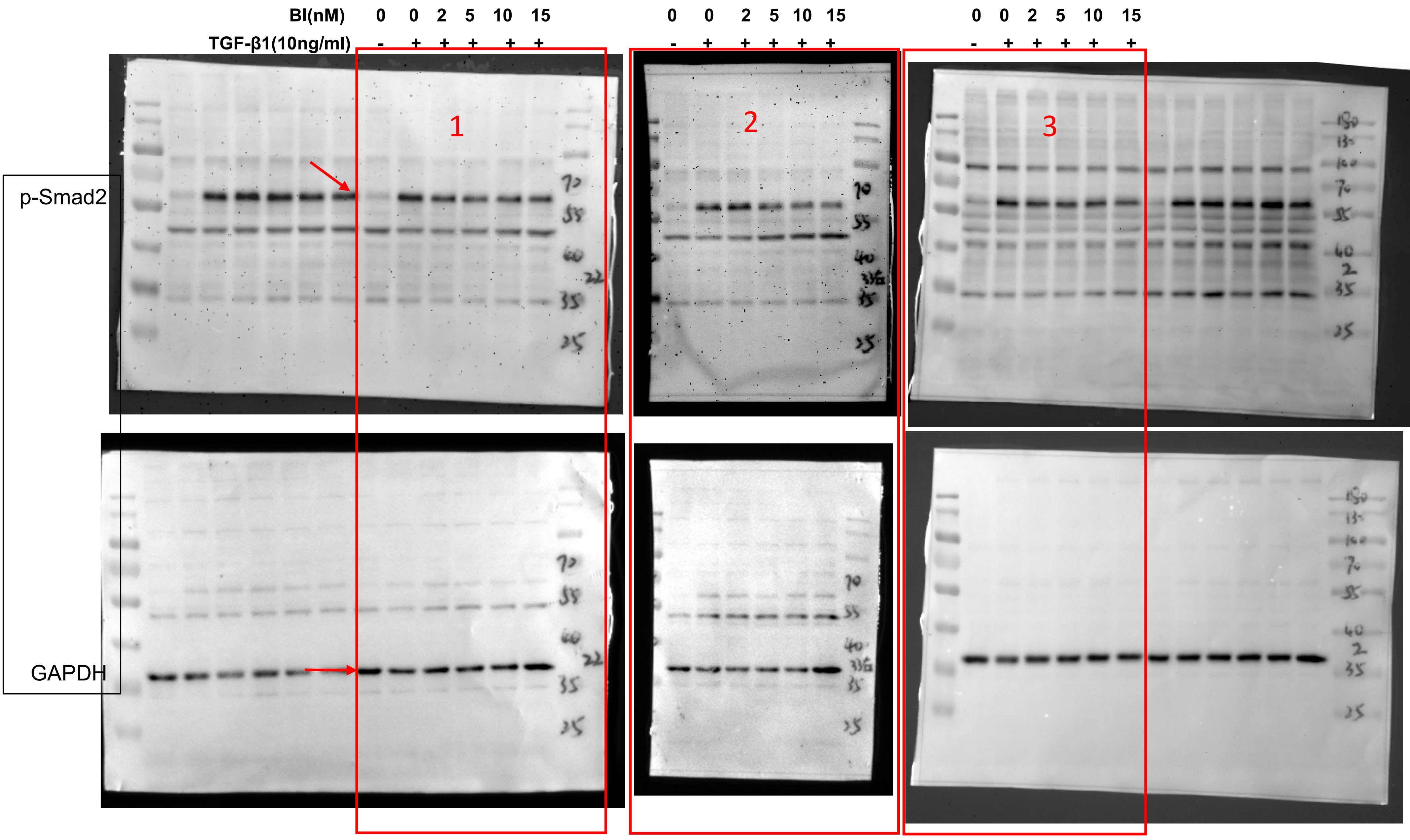
BI(nM) 0 0 2 5 10 15
TGF-β1(10ng/ml) - + + + + +
0 0 2 5 10 15
- + + + + +
0 0 2 5 10 15
- + + + + +
1
2
3
p-Smad2
GAPDH

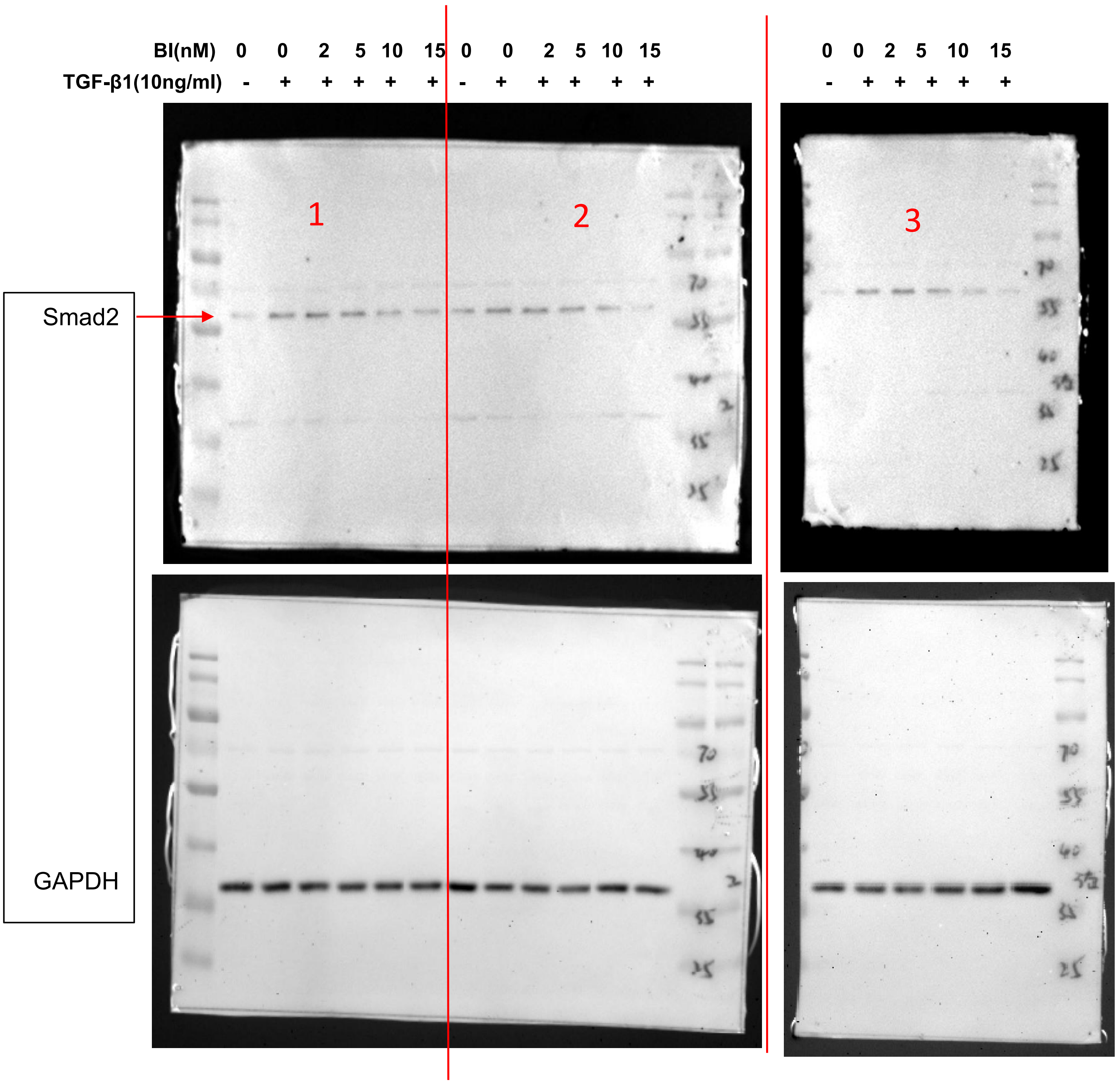
BI(nM) 0 0 2 5 10 15 0 0 2 5 10 15
TGF-β1(10ng/ml) - + + + + + - + + + + +
0 0 2 5 10 15
- + + + + +
1
2
3
Smad2
GAPDH

Figure6:

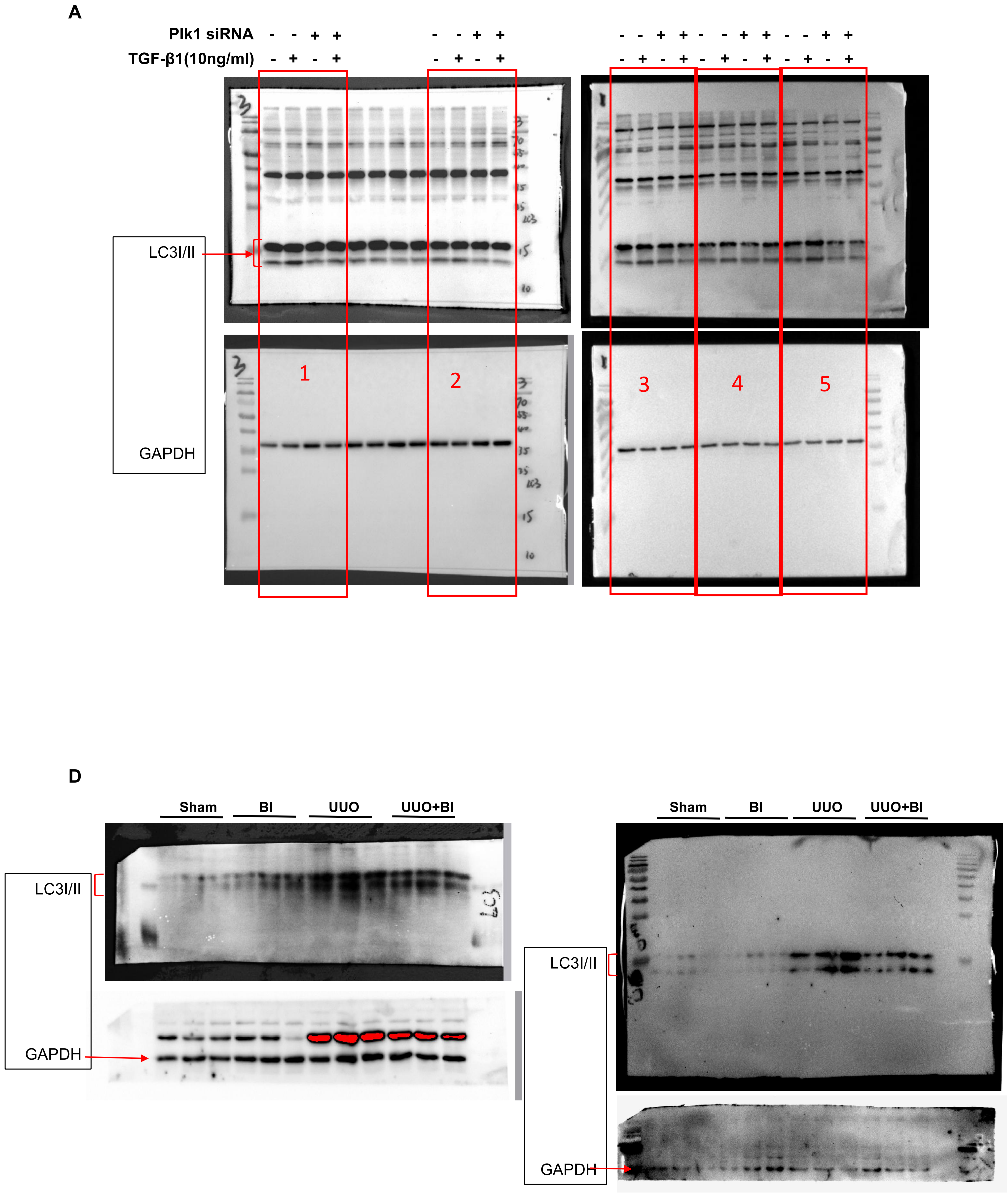
A
Plk1 siRNA
TGF-β1(10ng/ml)
LC3I/II
GAPDH
1
2
3
4
5
D
Sham
BI
UUO
UUO+BI
LC3I/II
GAPDH
Sham
BI
UUO
UUO+BI
LC3I/II
GAPDH

**E**

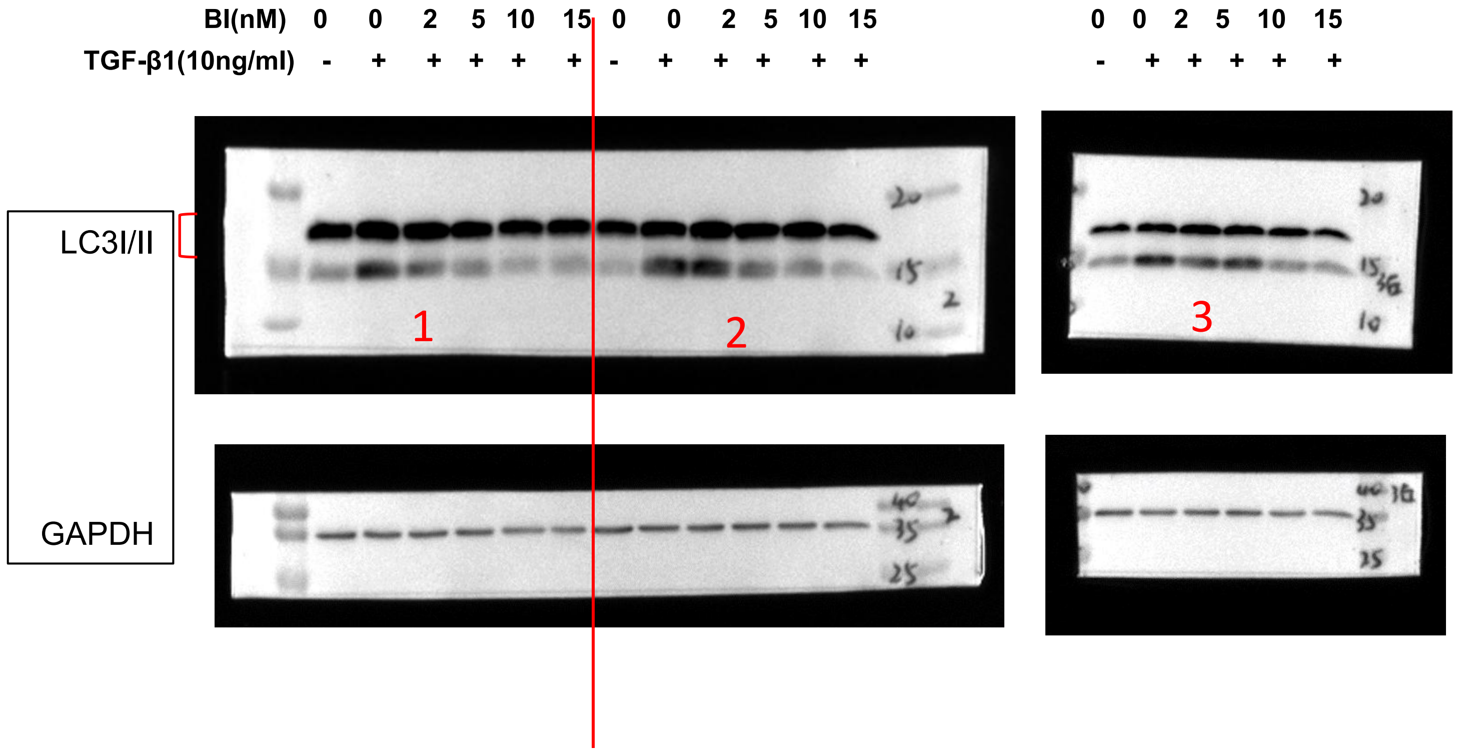
BI(nM) 0 0 2 5 10 15 0 0 2 5 10 15
TGF-β1(10ng/ml) - + + + + + - + + + + +
0 0 2 5 10 15
- + + + + +
LC3I/II
GAPDH
1
2
3

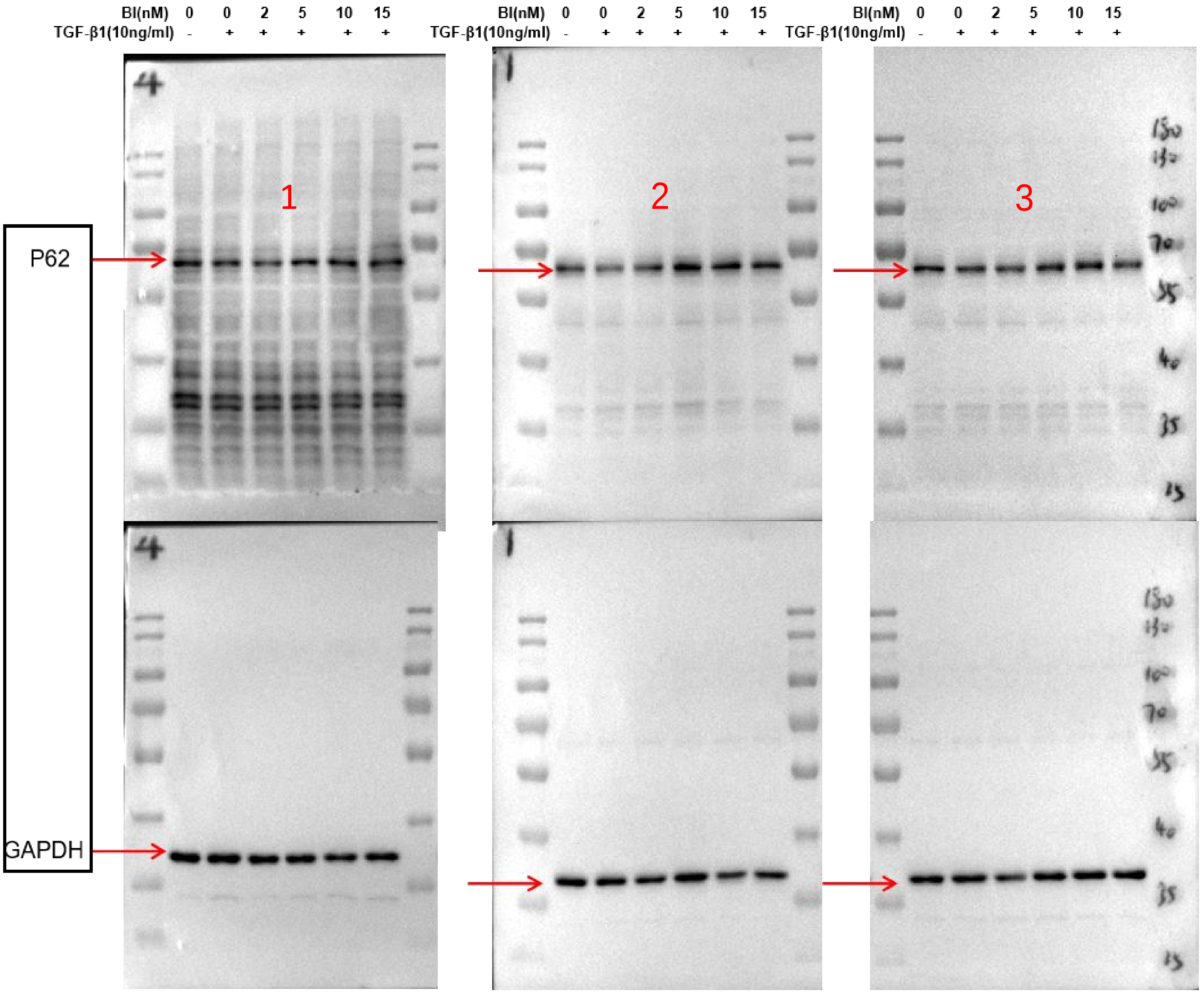
BI(nM) 0 0 2 5 10 15
TGF-β1(10ng/ml) - + + + + +
BI(nM) 0 0 2 5 10 15
TGF-β1(10ng/ml) - + + + + +
BI(nM) 0 0 2 5 10 15
TGF-β1(10ng/ml) - + + + + +
P62
GAPDH
1
2
3

Figure7:

A

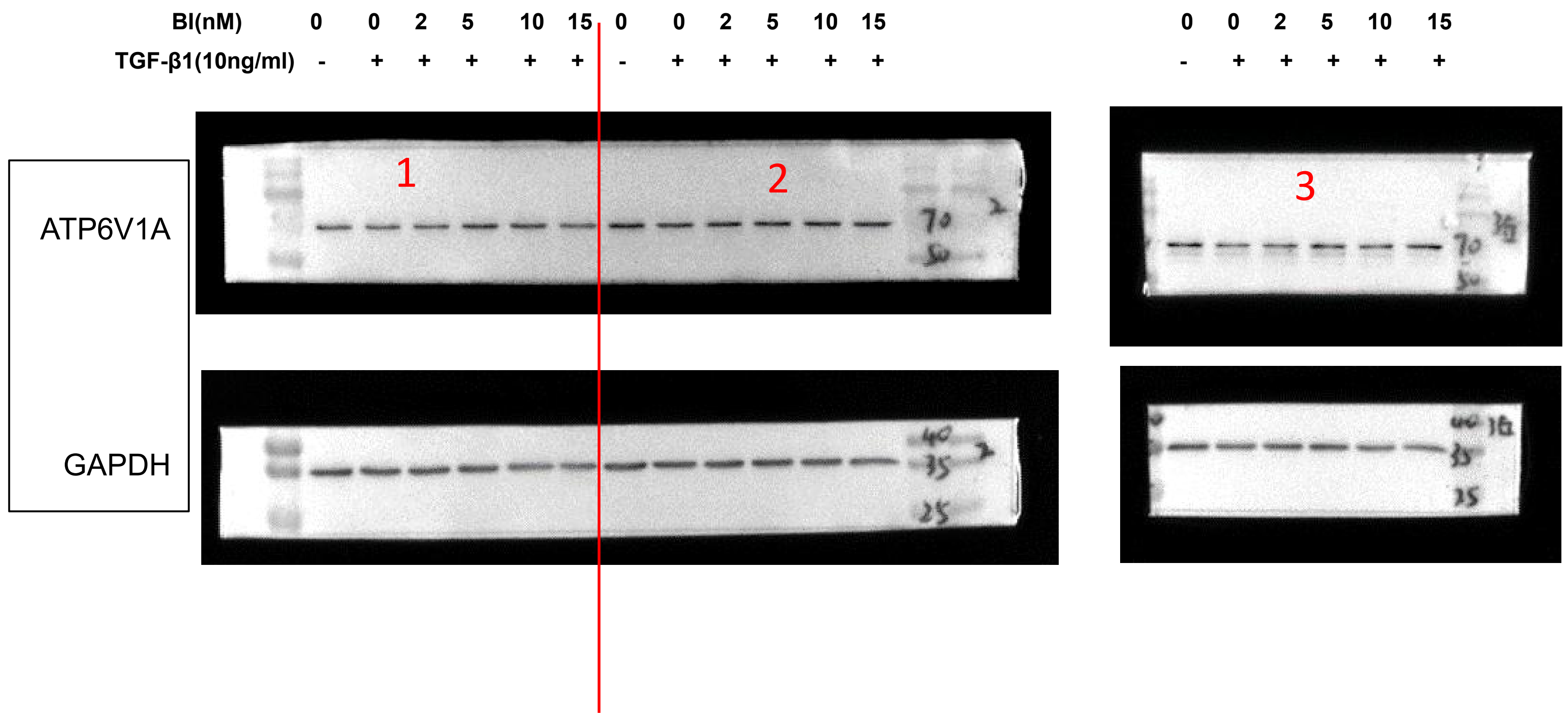


B

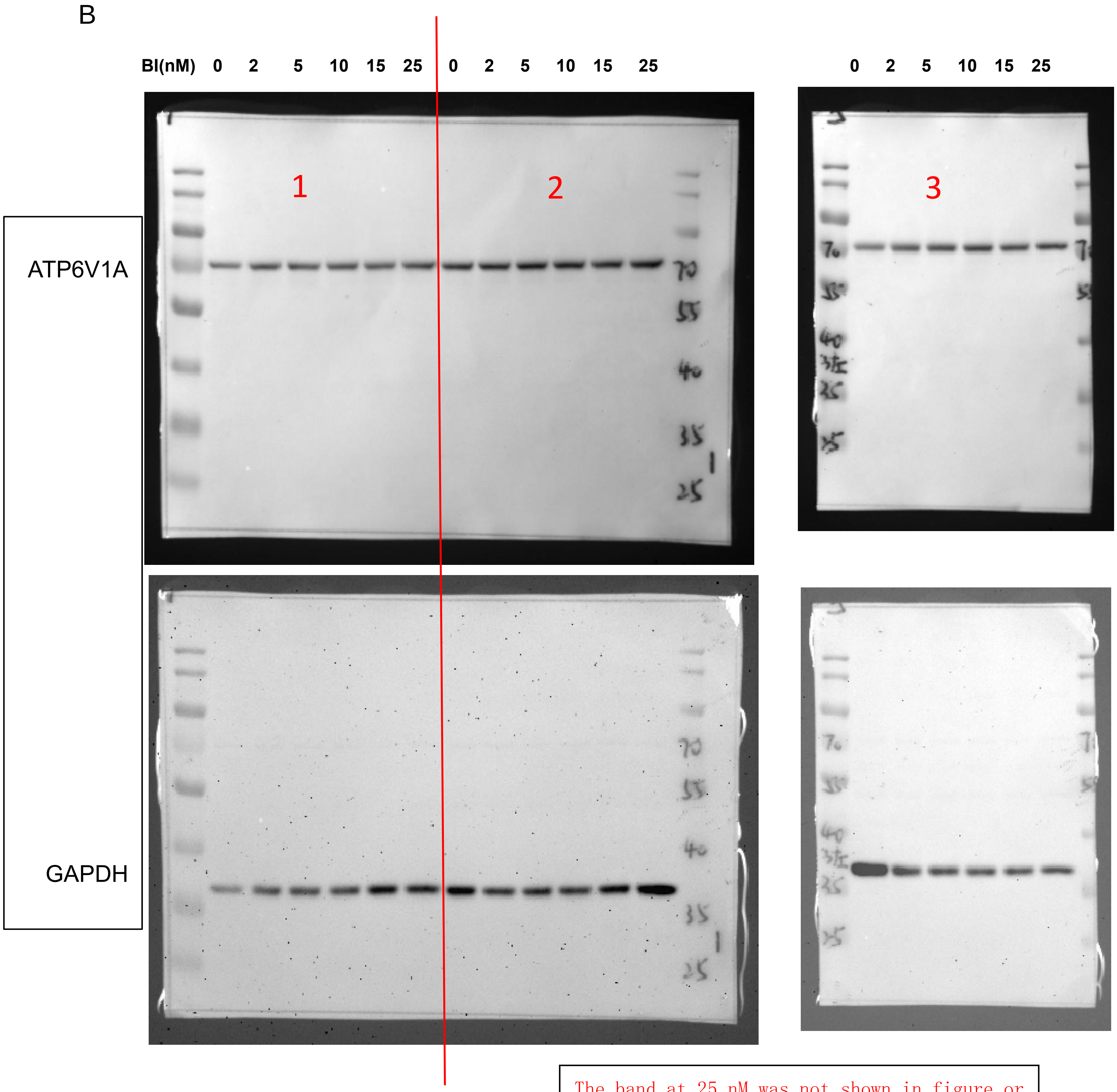


The band at 25 nM was not shown in figure or included for analysis because this concentration is toxic for cells.

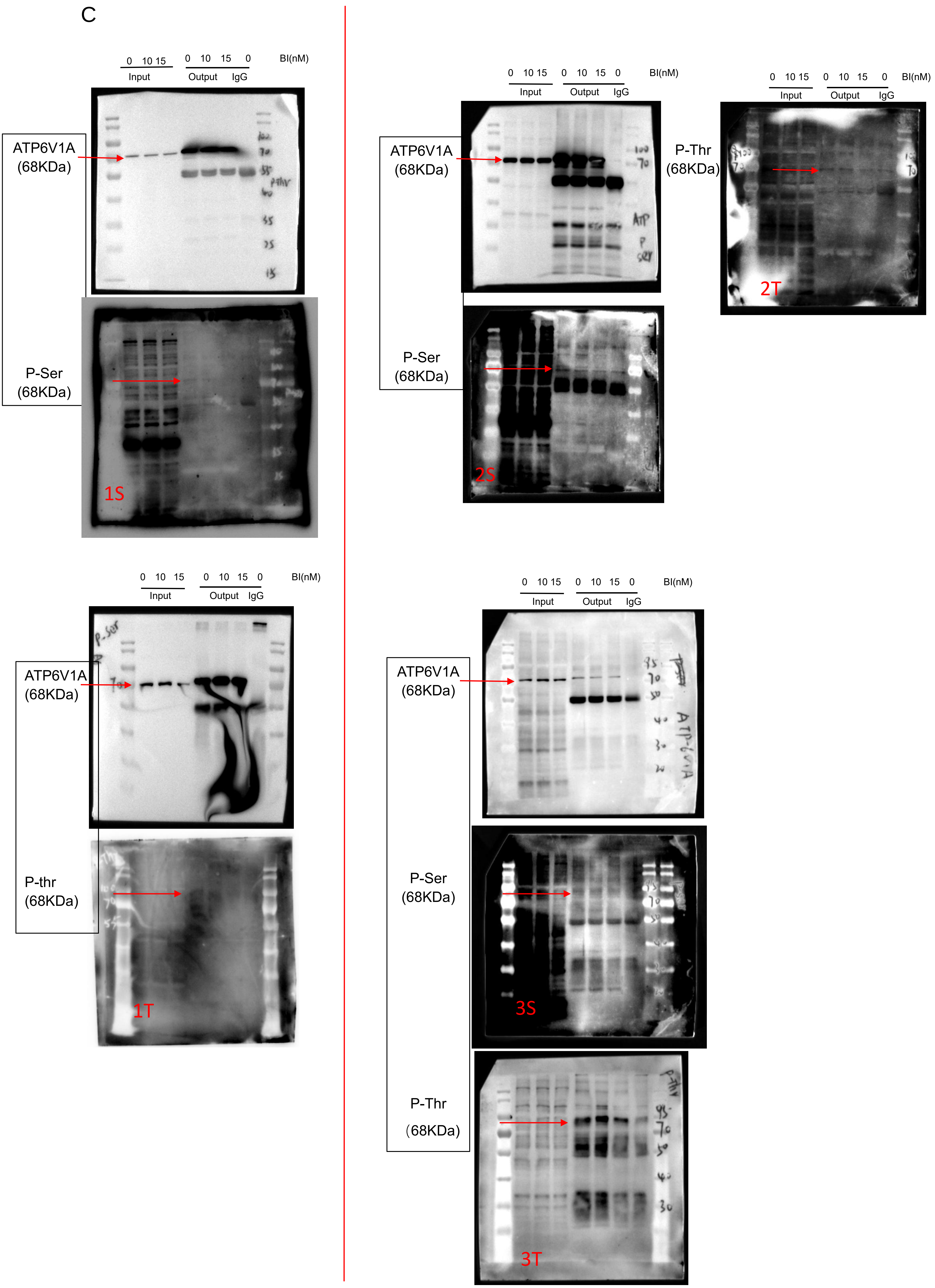
C
0 10 15
Input
0 10 15 0
Output
IgG
BI(nM)
ATP6V1A
(68KDa)
P-Ser
(68KDa)
P-thr
(68KDa)
P-Thr
(68KDa)
1S
2S
2T
1T
3S
3T

D

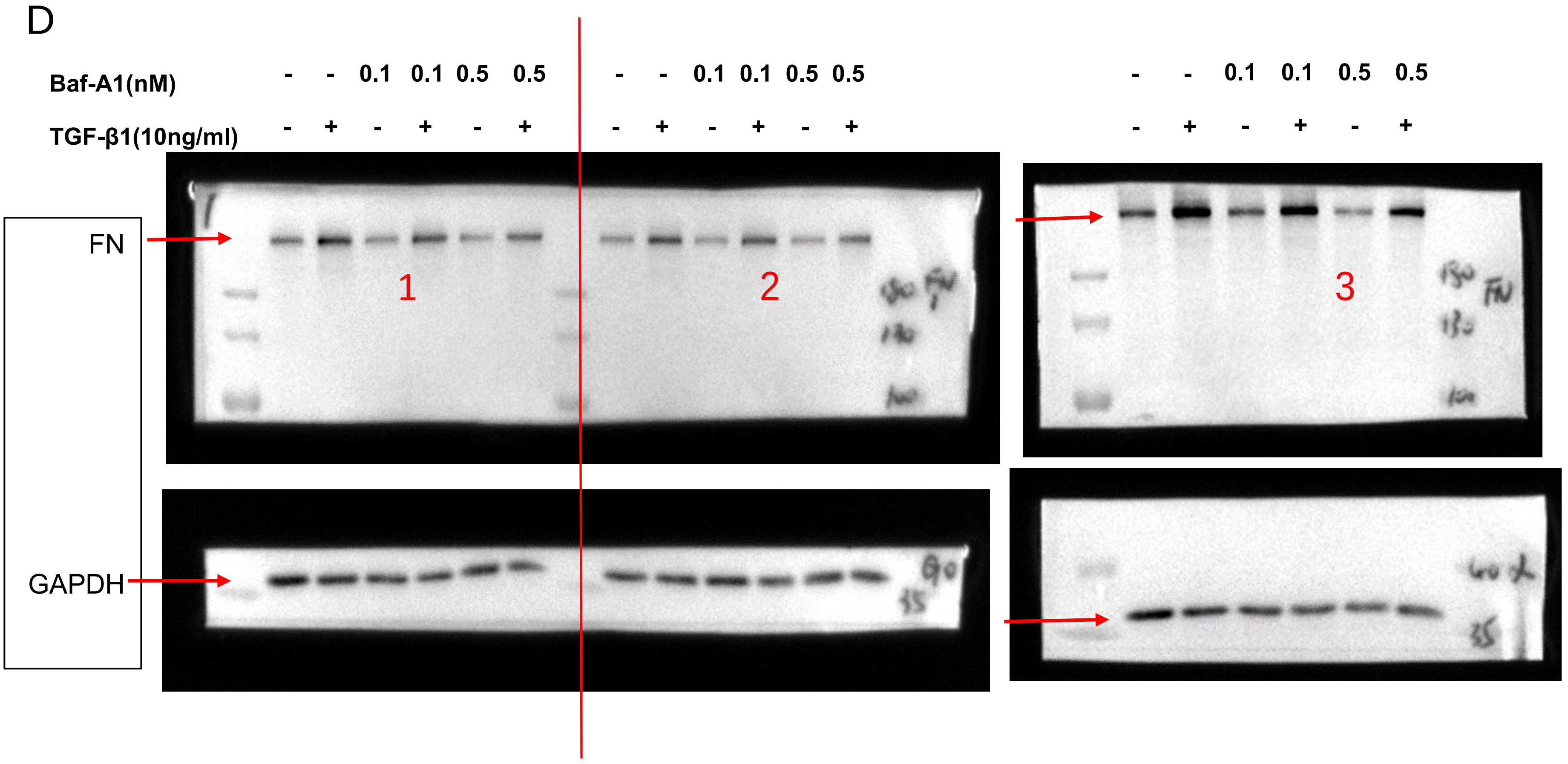
Baf-A1(nM) - - 0.1 0.1 0.5 0.5 - - 0.1 0.1 0.5 0.5 - - 0.1 0.1 0.5 0.5
TGF-β1(10ng/ml) - + - + - + - + - + - + - + - + - +
FN
GAPDH
1
2
3

E

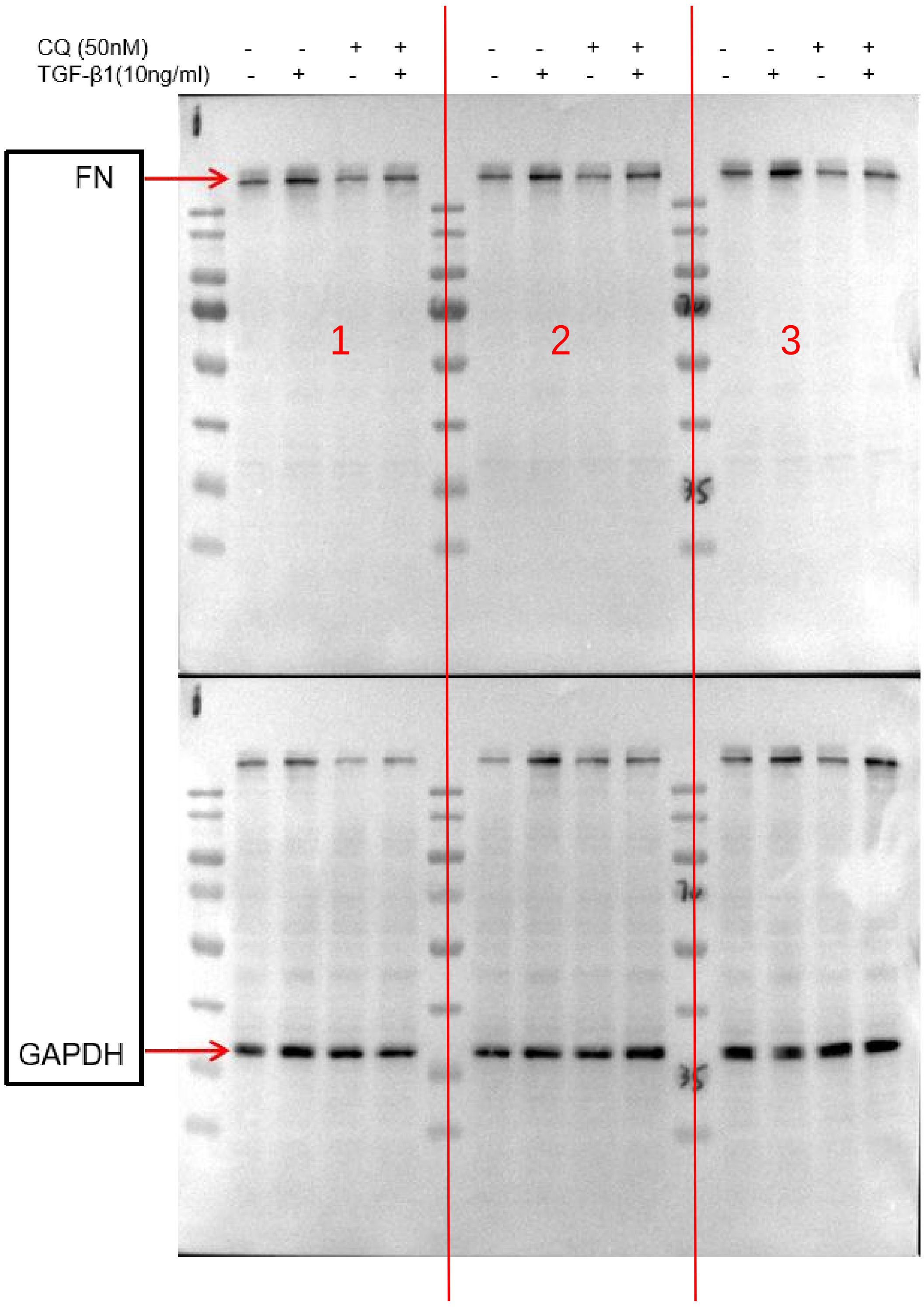
CQ (50nM) - - + + - - + + - - + +
TGF-β1(10ng/ml) - + - + - + - + - + - +
FN
GAPDH
1
2
3

F

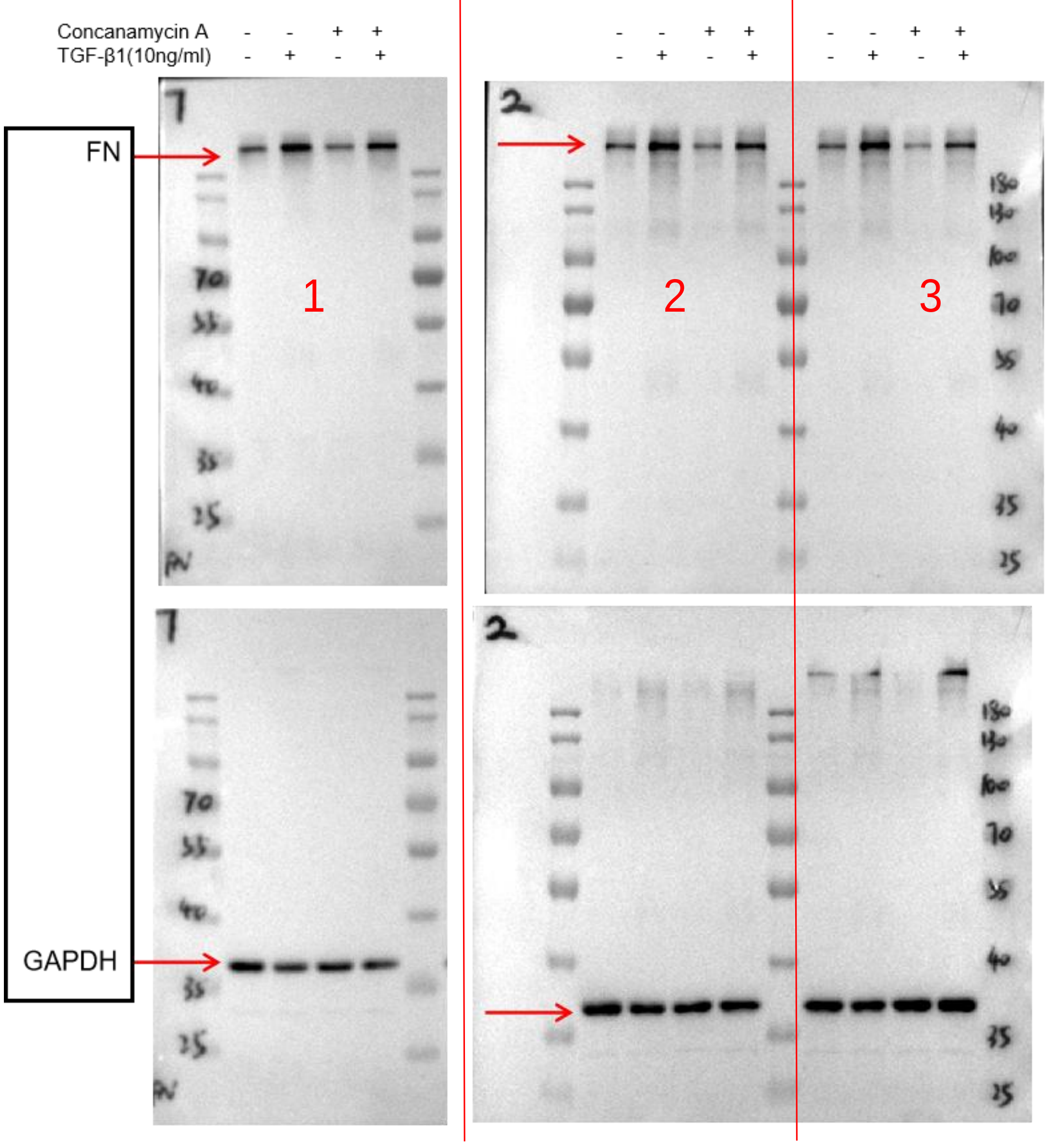
Concanamycin A - - + +
TGF-β1(10ng/ml) - + - +
- - + +
- + - +
- - + +
- + - +
FN
GAPDH
1
2
3

G

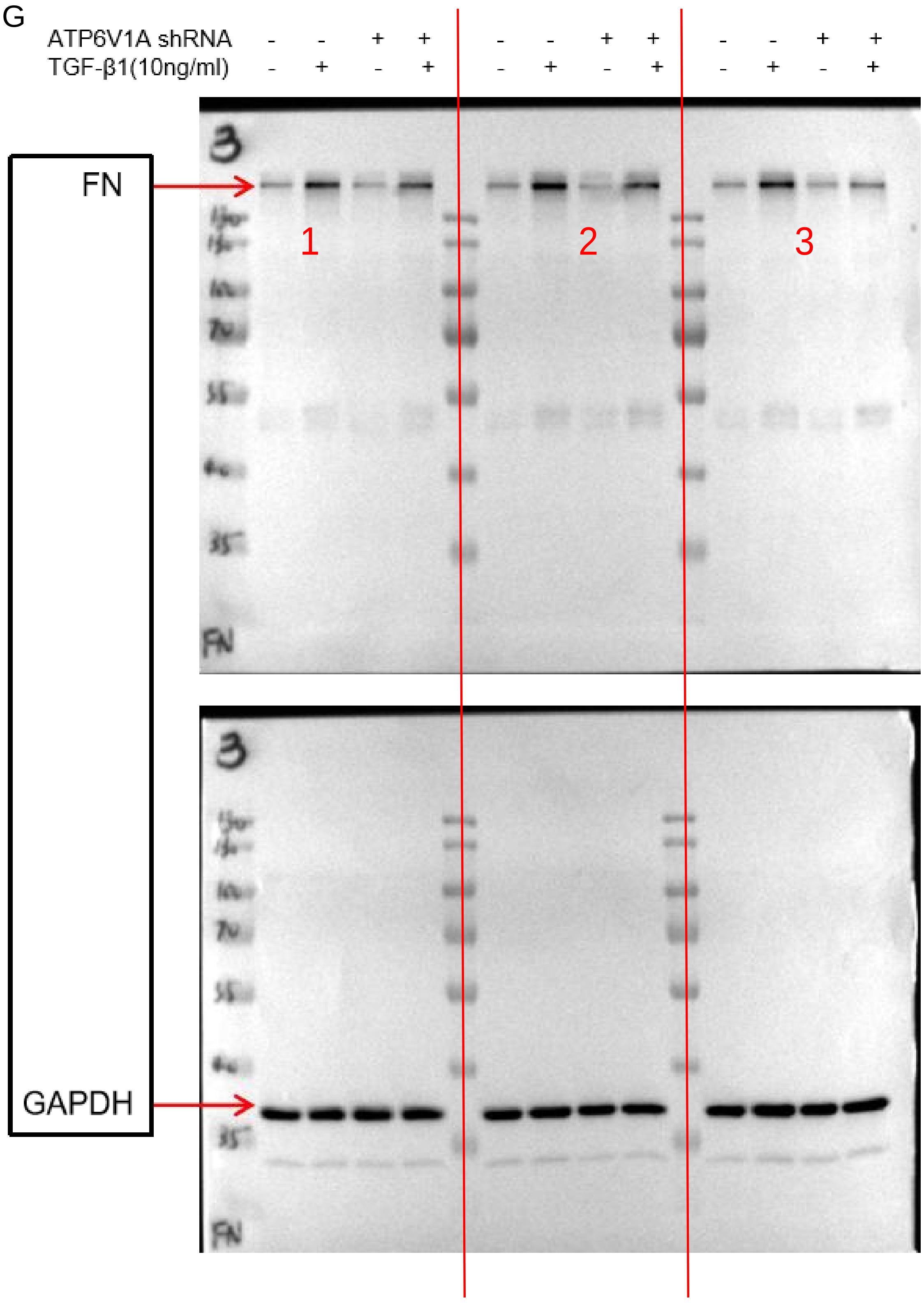
ATP6V1A shRNA - - + + - - + + - - + +
TGF-β1(10ng/ml) - + - + - + - + - + - +
FN
GAPDH
1
2
3

Figure8:

C

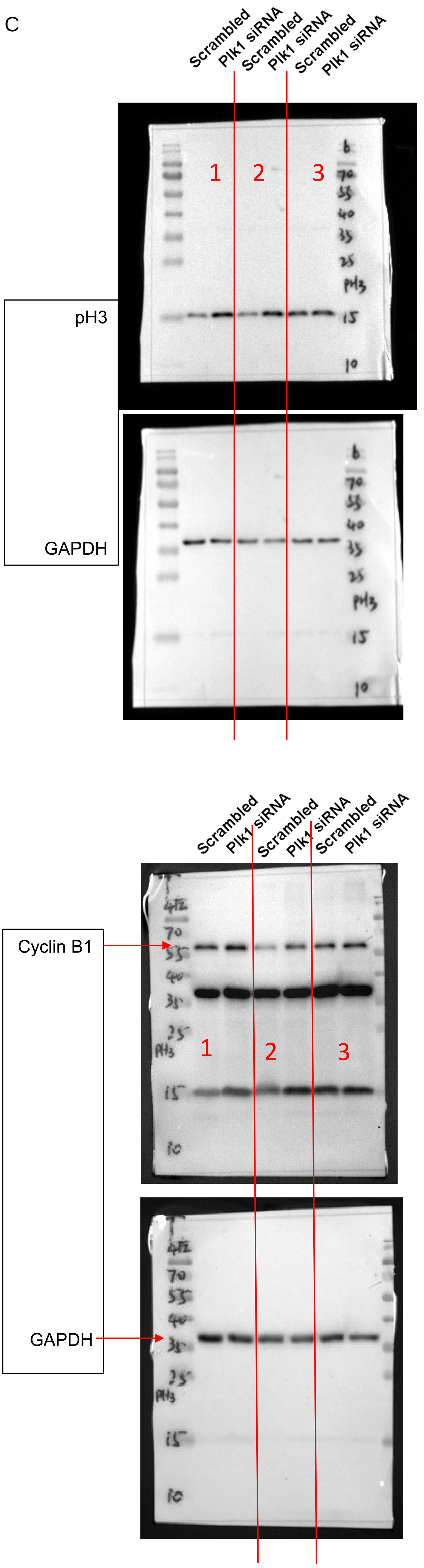
Scrambled
Plk1 siRNA
Scrambled
Plk1 siRNA
Scrambled
Plk1 siRNA
1
2
3
pH3
GAPDH
Scrambled
Plk1 siRNA
Scrambled
Plk1 siRNA
Scrambled
Plk1 siRNA
Cyclin B1
1
2
3
GAPDH

E

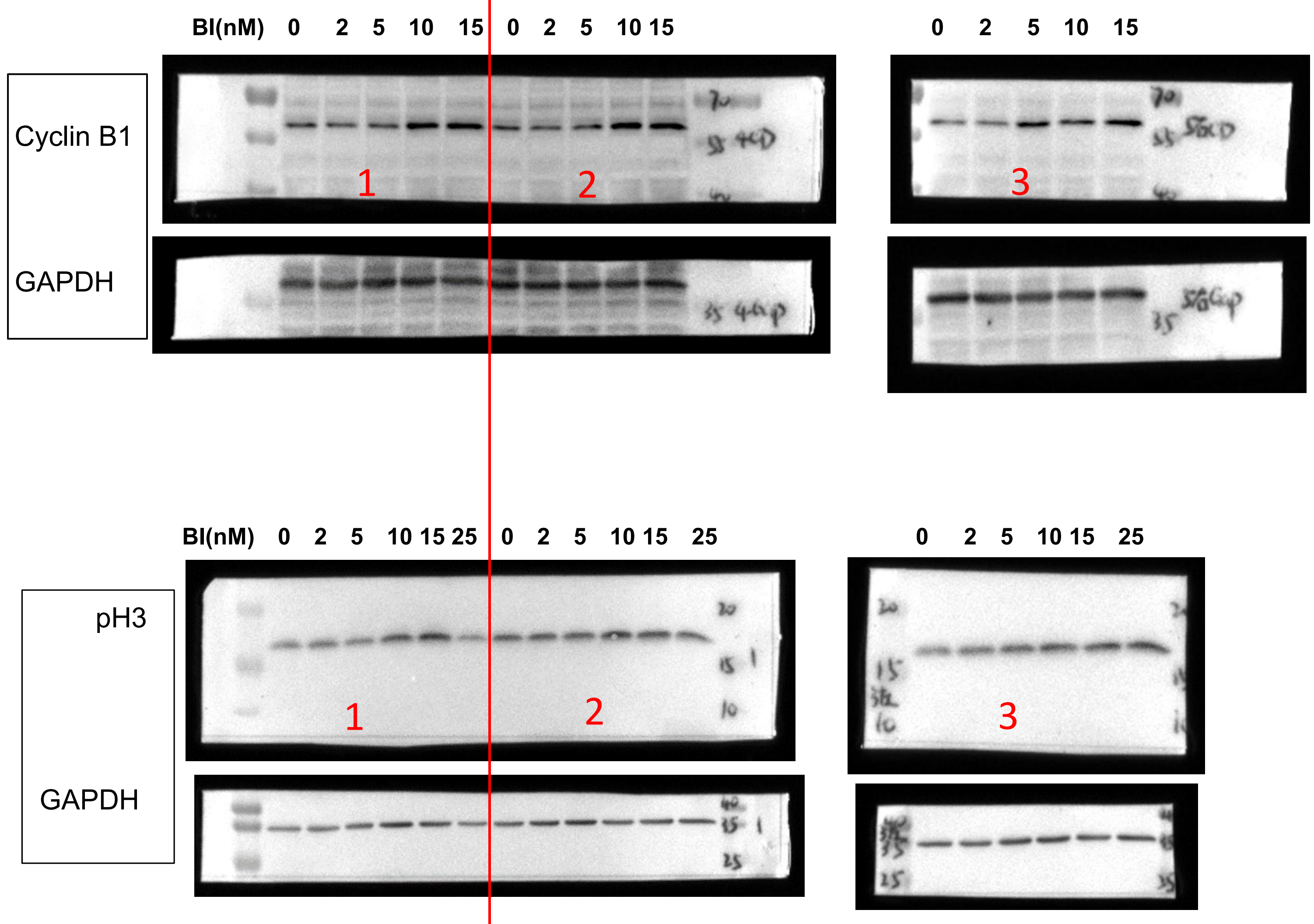


The band at 25 nM was not shown in figure or included for analysis because BI at this concentration is toxic for cells.

F

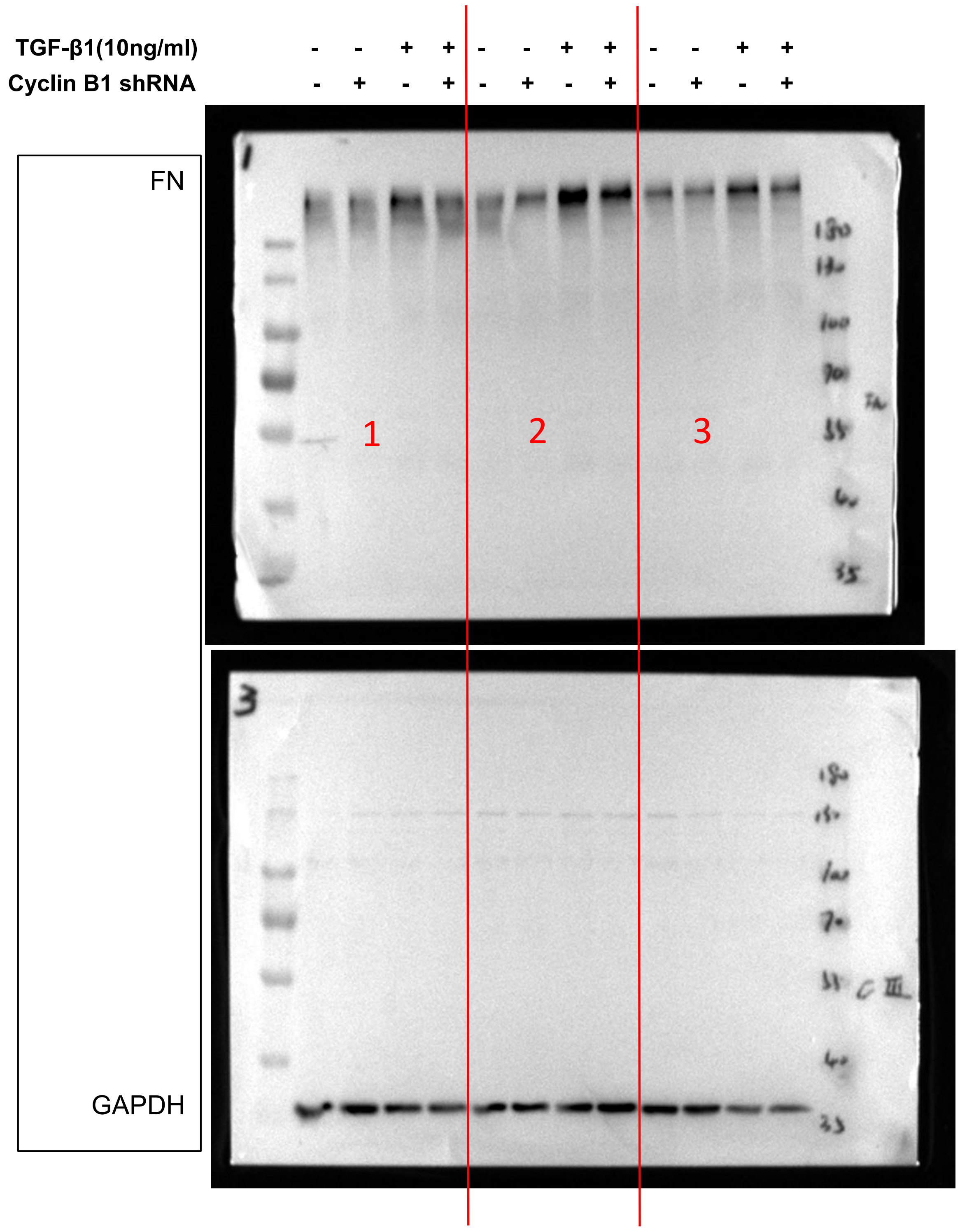

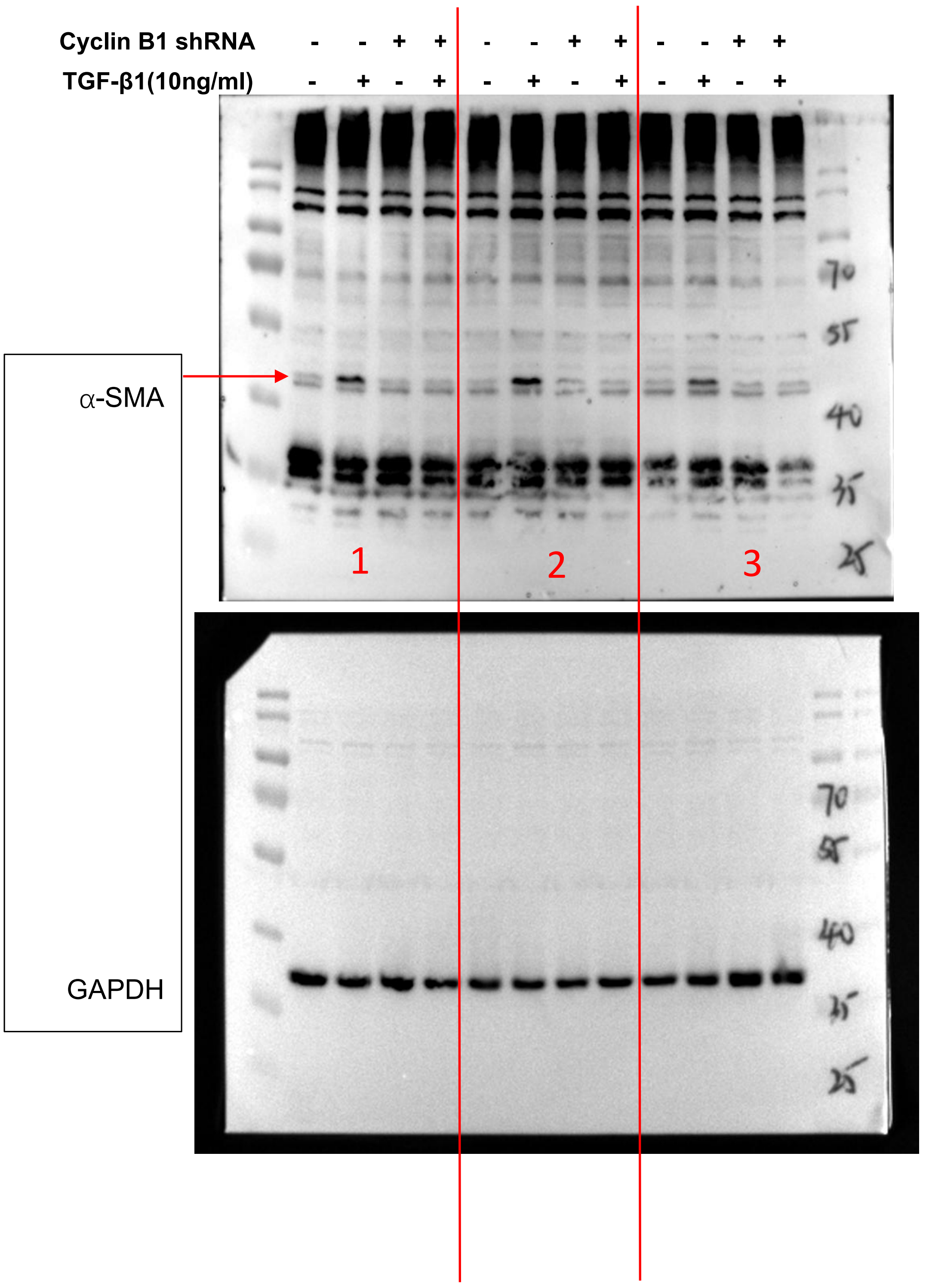
Cyclin B1 shRNA - - + + - - + + - - + +
TGF-β1(10ng/ml) - + - + - + - + - + - +
α-SMA
GAPDH
1
2
3
70
55
40
35
25

G

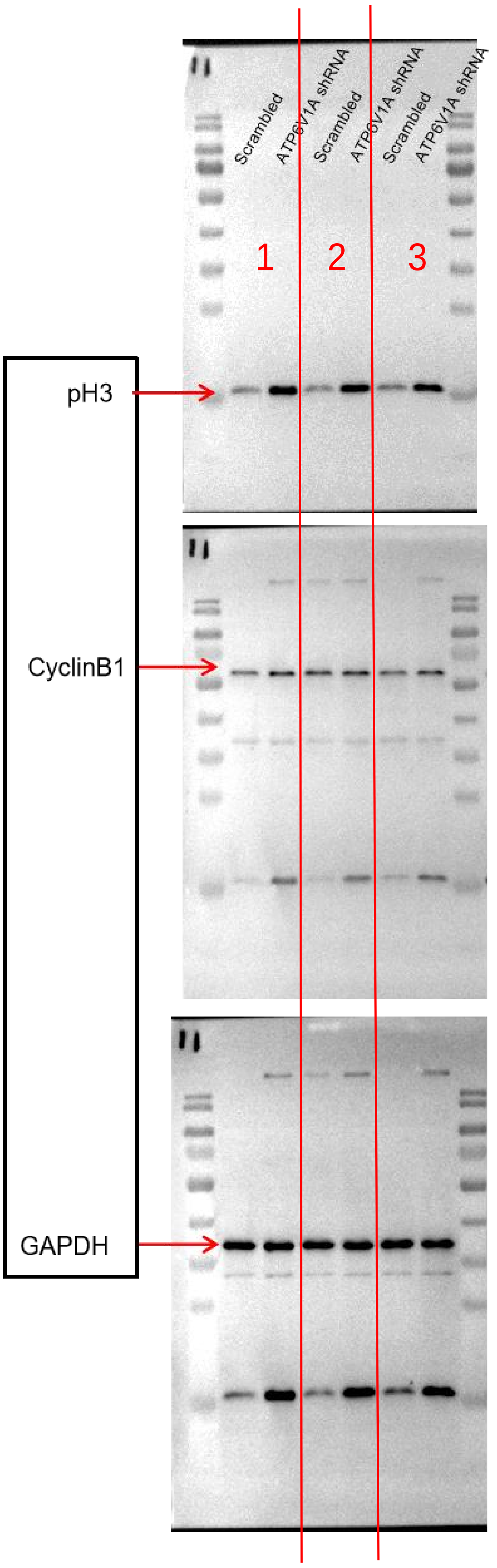

Scrambled
ATP6V1A shRNA
Scrambled
ATP6V1A shRNA
Scrambled
ATP6V1A shRNA
1
2
3
pH3
CyclinB1
GAPDH

Figure9:

**A**

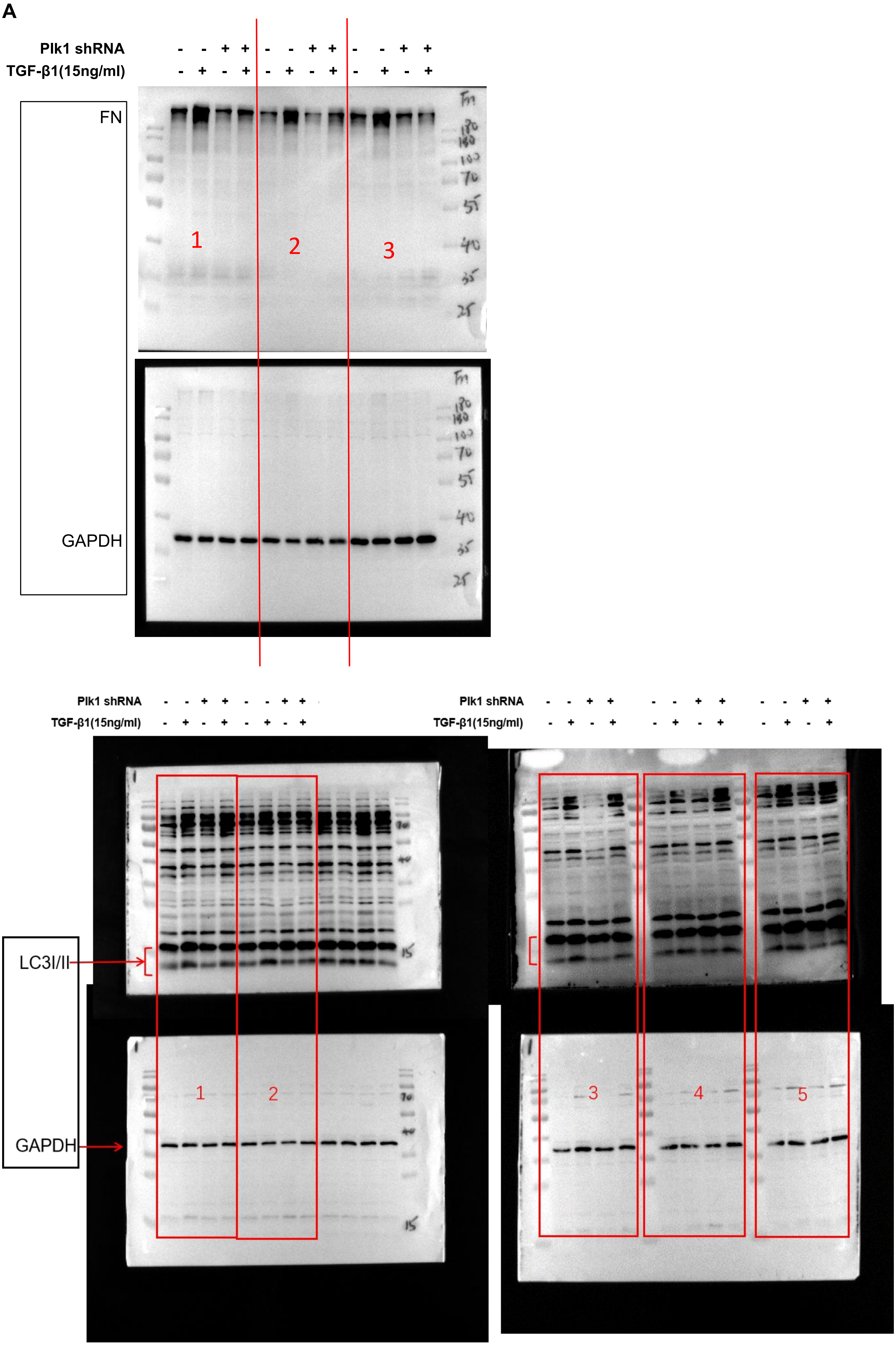
Plk1 shRNA
TGF-β1(15ng/ml)
FN
GAPDH
1
2
3
Plk1 shRNA
TGF-β1(15ng/ml)
Plk1 shRNA
TGF-β1(15ng/ml)
LC3I/II
GAPDH
1
2
3
4
5

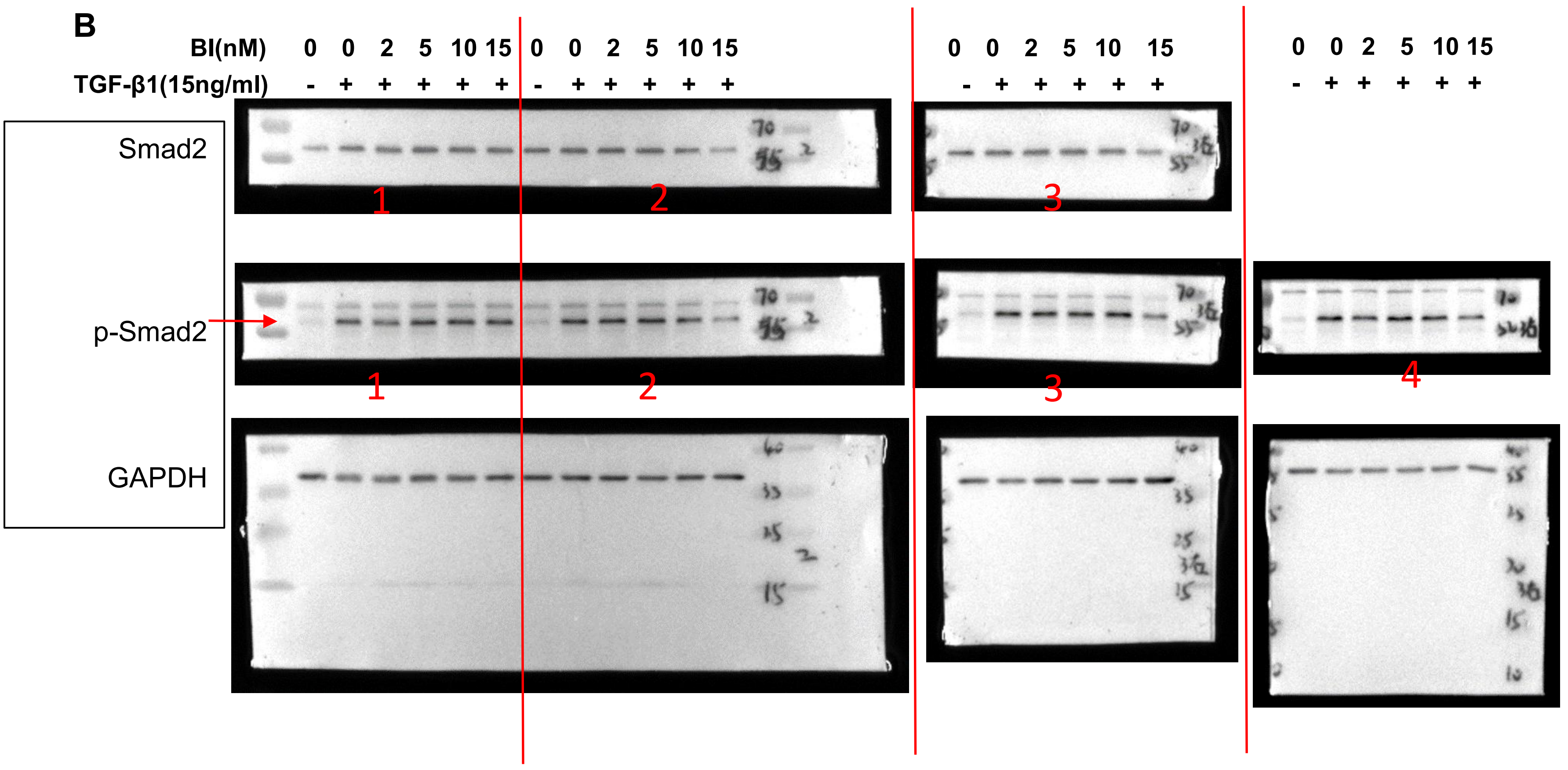
B
BI(nM) 0 0 2 5 10 15 0 0 2 5 10 15
TGF-β1(15ng/ml) - + + + + + - + + + + +
0 0 2 5 10 15
- + + + + +
0 0 2 5 10 15
- + + + + +
Smad2
p-Smad2
GAPDH
1
2
3
4

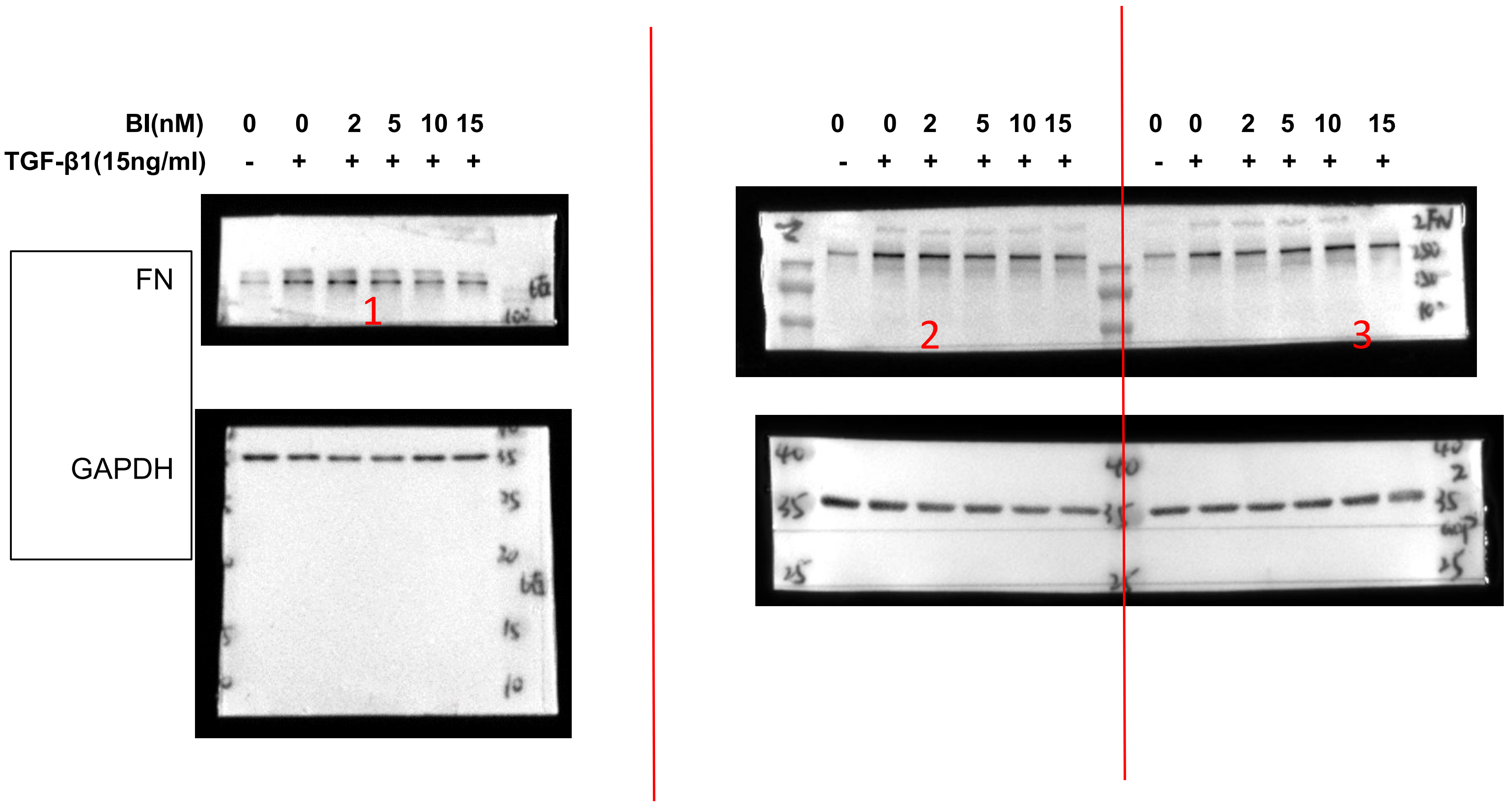
BI(nM) 0 0 2 5 10 15
TGF-β1(15ng/ml) - + + + + +
0 0 2 5 10 15
- + + + + +
0 0 2 5 10 15
- + + + + +
FN
GAPDH
1
2
3

**C**

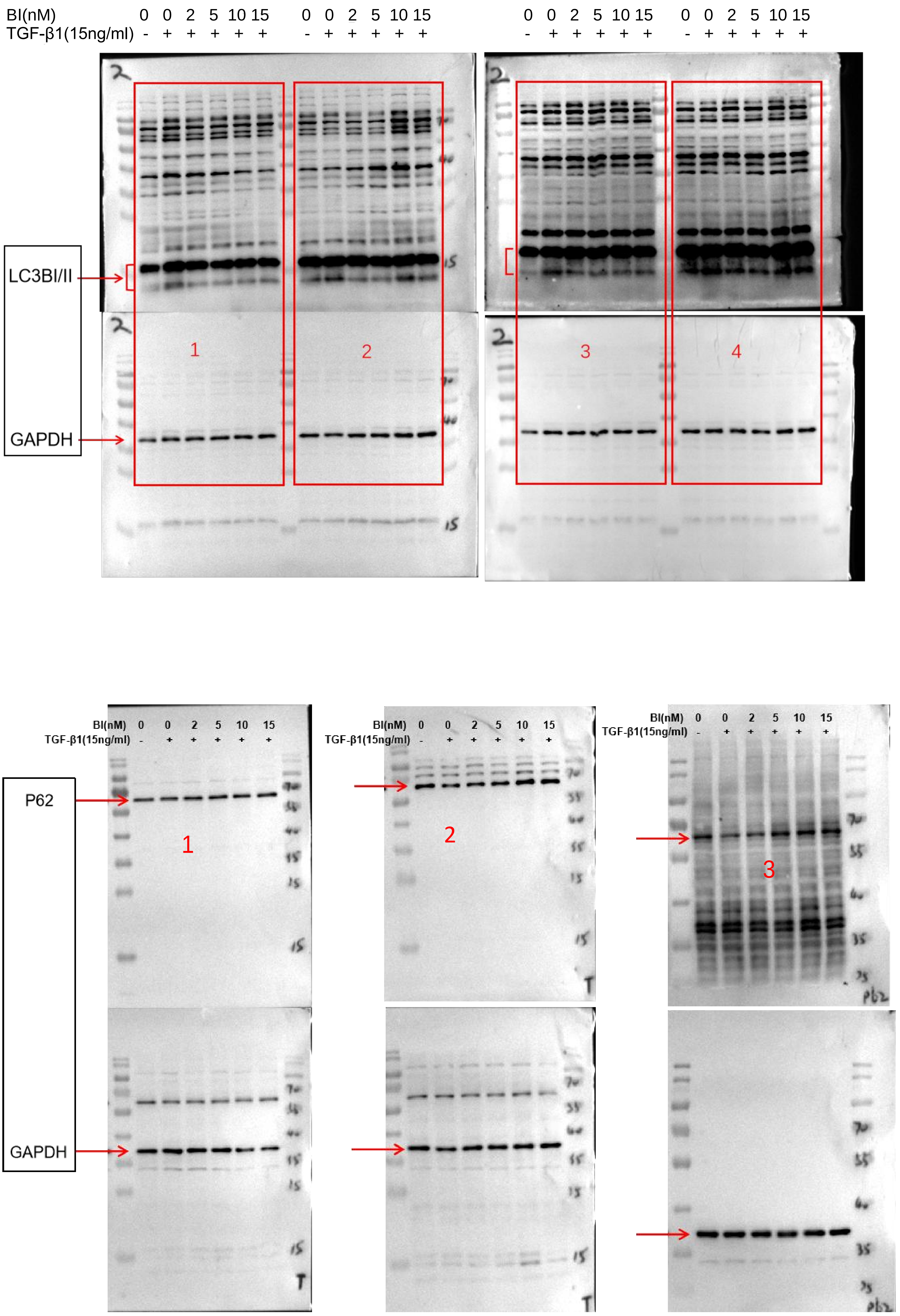
BI(nM) 0 0 2 5 10 15 0 0 2 5 10 15 0 0 2 5 10 15 0 0 2 5 10 15
TGF-β1(15ng/ml) - + + + + + - + + + + + - + + + + + - + + + + +
LC3BI/II
GAPDH
1
2
3
4
BI(nM) 0 0 2 5 10 15
TGF-β1(15ng/ml) - + + + + +
P62
GAPDH

E

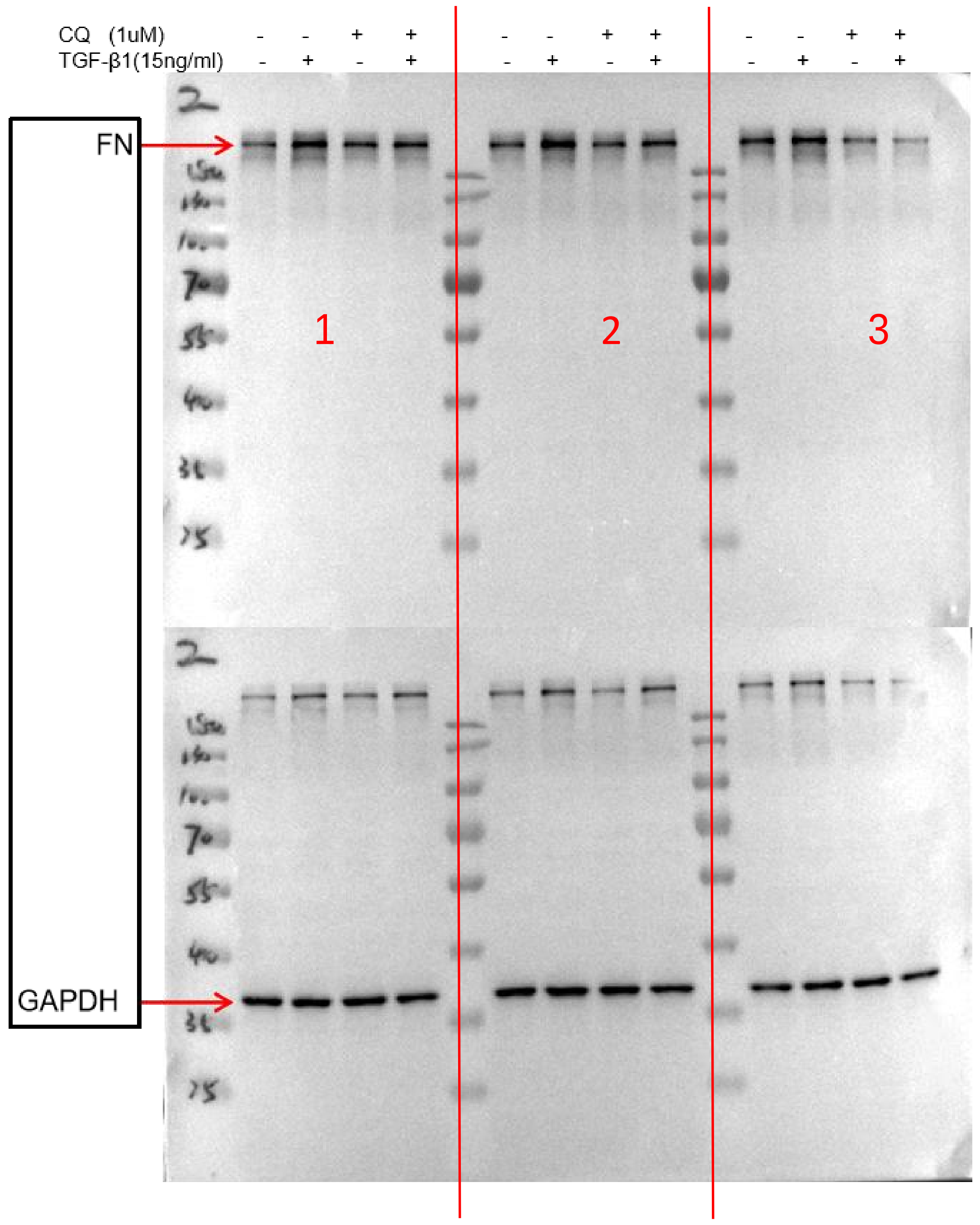

CQ (1uM)
TGF-β1(15ng/ml)
FN
GAPDH
1
2
3


F

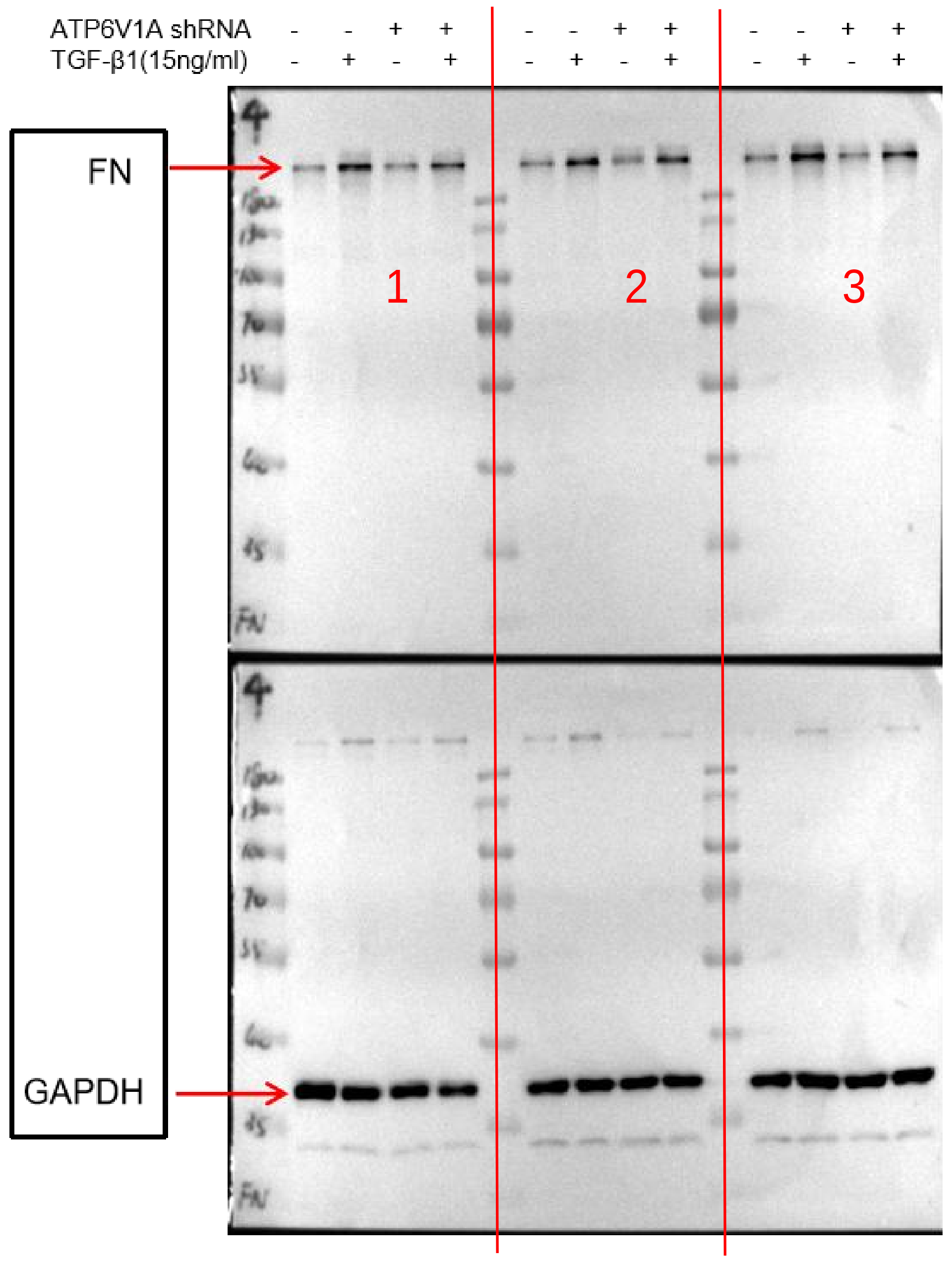

ATP6V1A shRNA
TGF-β1(15ng/ml)
FN
GAPDH
1
2
3

G

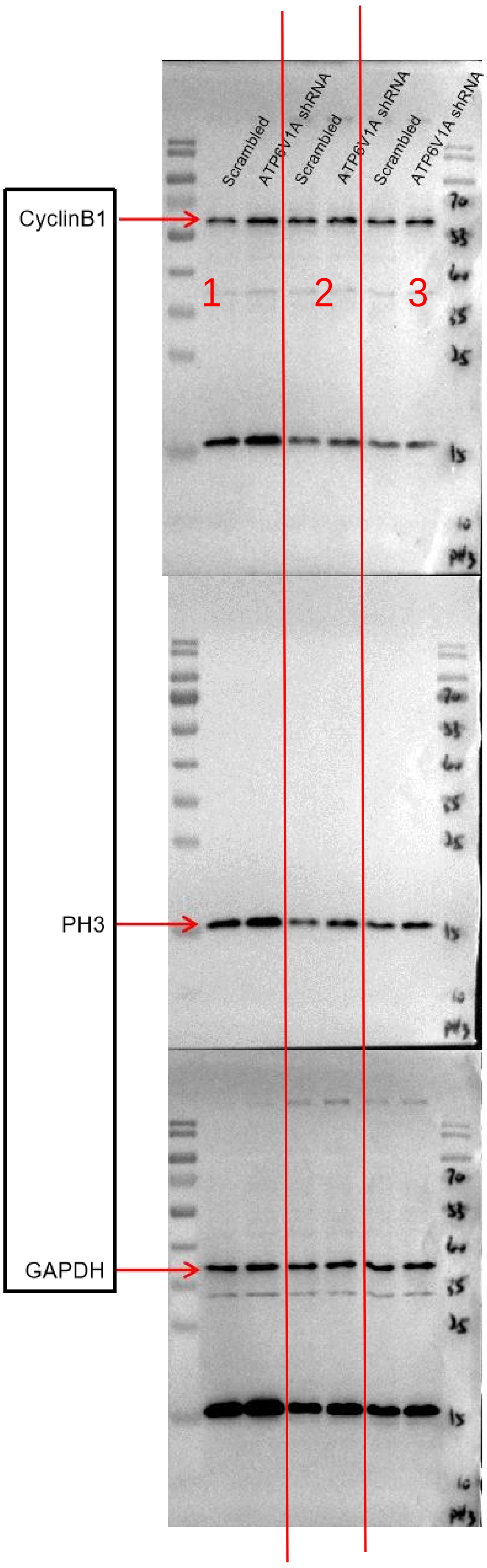
CyclinB1
PH3
GAPDH
Scrambled
ATP6V1A shRNA
Scrambled
ATP6V1A shRNA
Scrambled
ATP6V1A shRNA
1
2
3

Figure S1

A

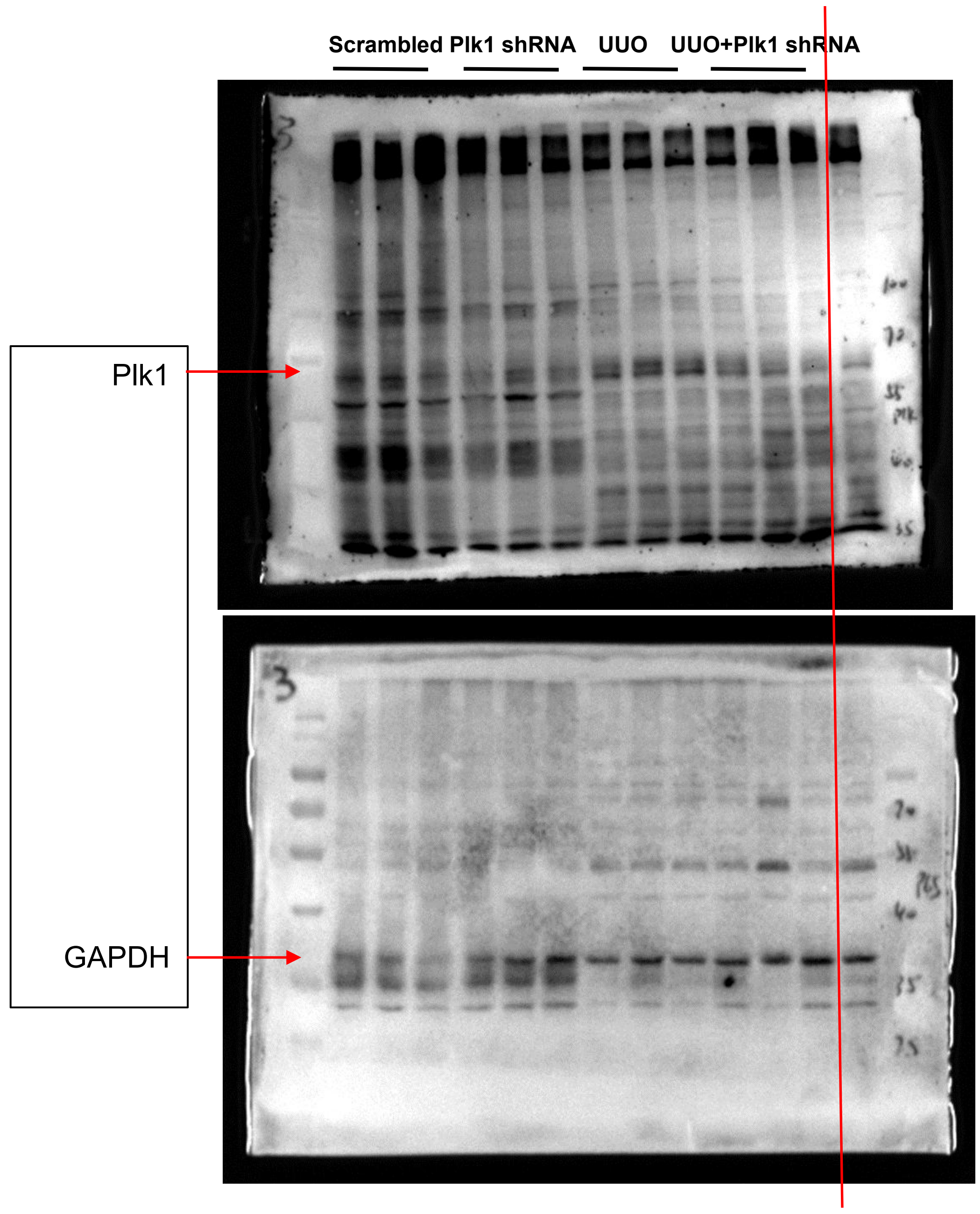


The last well is loaded with other kidney sample just for balancing of the gel. It is not included for analysis.

C

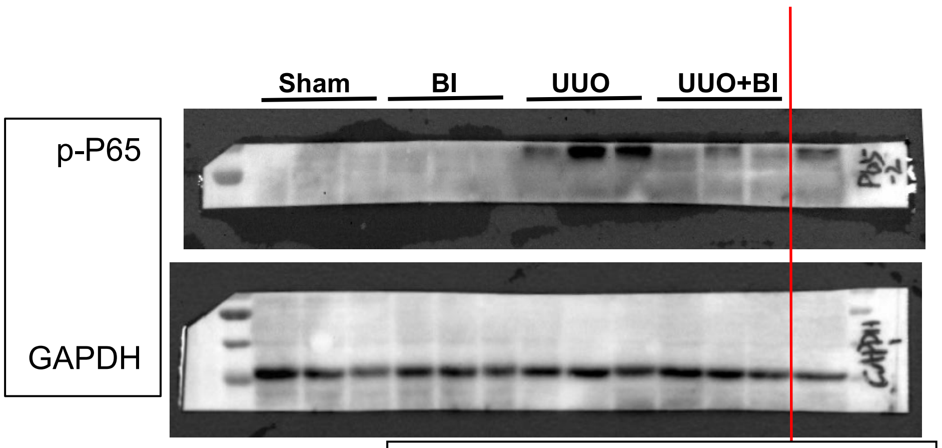


The last well is loaded with other kidney sample just for balancing of the gel. It is not included for analysis.

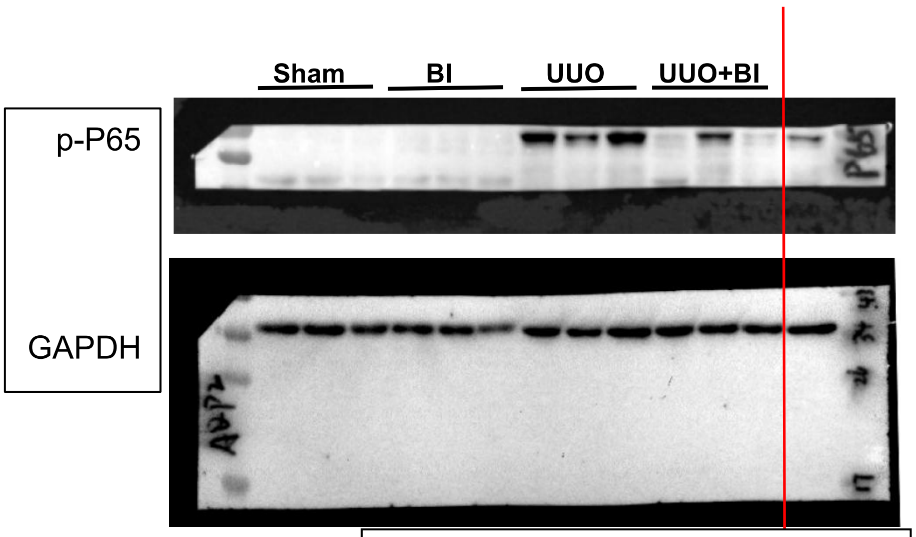


The last well is loaded with other kidney sample just for balancing of the gel. It is not included for analysis.

Fig.S5

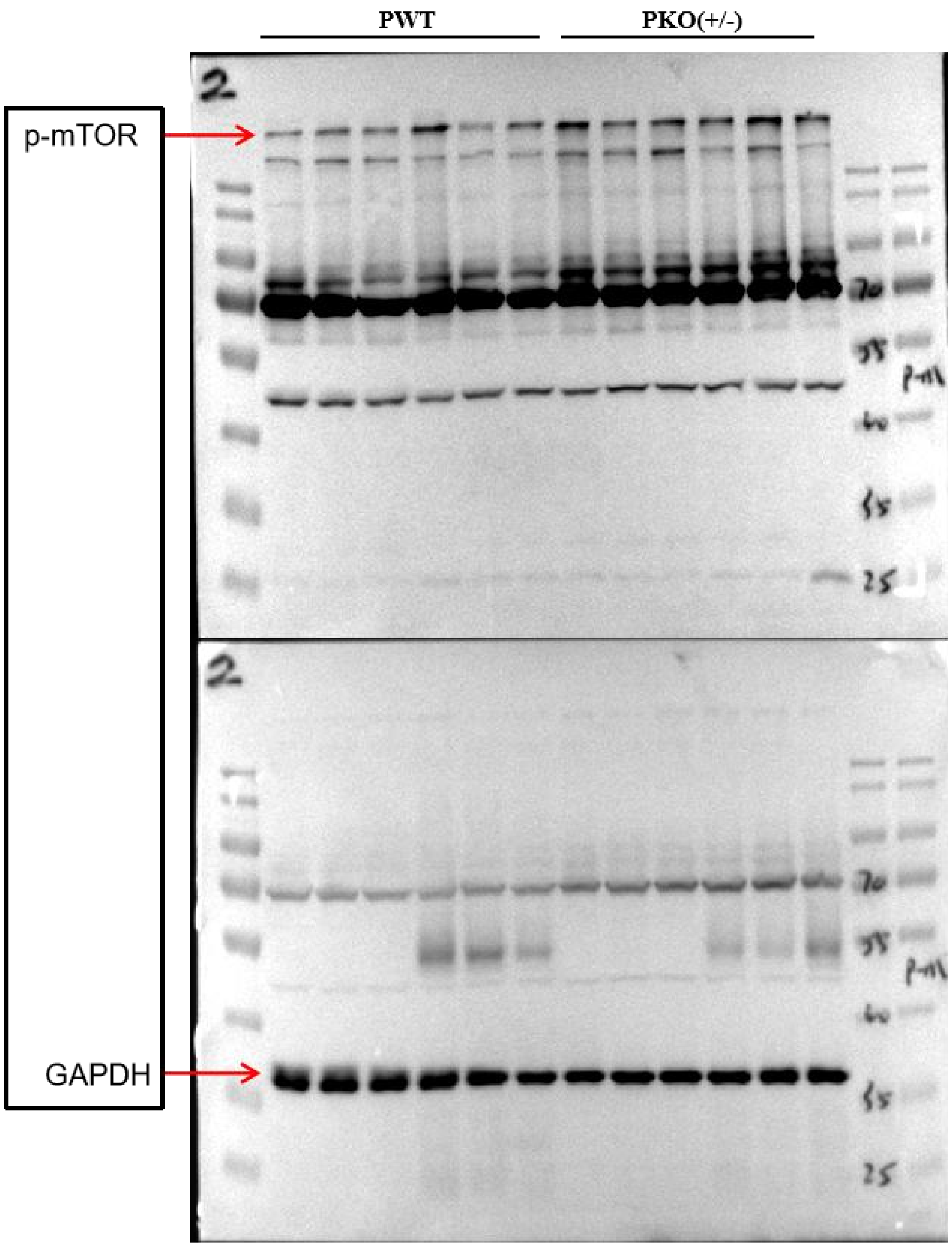


Fig.3C top FN, α-SMA and Fig.6D left LC3 are from same membrane by striping and reincubation with different antibodies, so they shared same GAPDH.

Fig.6E LC3 and Fig.7A ATP6V1A are from same membrane , so they shared same GAPDH.
